# The Association Between Disordered Eating and Sleep in Non‐Clinical Populations—A Systematic Review and Meta‐Analysis

**DOI:** 10.1111/jsr.70117

**Published:** 2025-06-30

**Authors:** Marie‐Christine Opitz, Nora Trompeter, Francisco Diego Rabelo‐da‐Ponte, Michelle Carroll, Kyle Buchan, Giulia Gaggioni, Sarah Moody, Sylvane Desrivières, Nadia Micali, Ulrike Schmidt, Helen Sharpe, EDIFY Consortium, Ulrike Schmidt, Helen Sharpe, Karina Allen, Heike Bartel, Iain Campbell, Sylvane Desrivières, Richard Dobson, Amos Folarin, Tara French, John Kelly, Umairah Malik, Nadia Micali, Sneha Raman, Zulqarnain Rashid, Janet Treasure, Callum Bryson, Daire Douglas, Amelia Hemmings, Başak İnce Çağlar, Carina Kuehne, Sarah Moody, Marie‐Christine Opitz, Tamsin Parnell, Fiona Stephens, Nora Trompeter, Jessica Wilkins, Alice Bromell, Grace Davis, Cameron Eadie, Beck Heslop, Isabella Malvisi, Katie Mckenzie, Emma Scott, Chris Sims, Tallulah Street, Andreia Tavares‐Semedo, Eleanor Wilkinson, Lucz Zocek

**Affiliations:** ^1^ Department of Clinical Psychology, School of Health in Social Sciences University of Edinburgh Scotland UK; ^2^ Great Ormond Street Institute of Child Health University College London UK; ^3^ Social Genetic and Developmental Psychiatry Centre Institute of Psychiatry, Psychology and Neuroscience, King's College London UK; ^4^ Division of Psychiatry Centre for Clinical Brain Sciences, University of Edinburgh UK; ^5^ Centre for Research in Eating and Weight Disorders Institute of Psychiatry, Psychology and Neuroscience, King's College London UK; ^6^ South London and Maudsley NHS Foundation Trust London UK

**Keywords:** disordered eating, meta‐analysis, sleep

## Abstract

Sleep and disordered eating behaviours may be linked through physiological and psychological mechanisms; yet, no review has systematically investigated the relationship between different sleep indicators and disordered eating behaviours and cognitions outside a clinical context. The present systematic review and meta‐analysis addressed this research gap to gain a better understanding of associations in non‐clinical populations to potentially inform future prevention and early intervention approaches in the context of both sleep and disordered eating. All studies published from 2003 onwards were included if they assessed a relationship between disordered eating and sleep in a non‐clinical population. In total, 89 studies were included, of which 33 met eligibility criteria for the meta‐analyses. General eating pathology, loss of control eating, and excessive exercise were most consistently significantly associated with poorer sleep quality and higher insomnia symptoms, while evening chronotypes were most consistently associated with bulimia symptoms, night eating, and body image concerns. Likely due to the limited evidence available, findings relating to restrictive eating behaviours and bulimia symptoms were largely mixed. Primarily small and non‐significant effects were found for associations between disordered eating and sleep duration measures. Overall, this review identified a need for more longitudinal research, the use of validated assessment methods, and studies focusing on restrictive eating, bulimia‐related behaviours, and excessive exercise. Despite the heterogeneity of study populations and designs, this review highlights sleep problems (e.g., insomnia symptoms, impaired sleep quality) as a transdiagnostic correlate of disordered eating concerns.

## 1 Introduction

Although eating disorder (ED) symptoms impact both the metabolic and circadian systems, links between disordered eating and sleep and circadian rhythm disturbances are still not sufficiently well understood (Christensen and Short 2021). The metabolic and circadian systems are interdependent in maintaining a normal physiological state, with circadian processes impacting metabolism in terms of digestion, appetite, and thermogenesis, while feeding behaviours contribute to synchronising circadian rhythms (Potter et al. 2016; Han et al. 2022). In the context of metabolic disorders, the interaction between metabolism and circadian rhythms has been extensively studied (Han et al. 2022). For instance, type‐2 diabetes has been associated with a desynchronization of the internal circadian system and external zeitgeber disruptions, with the most common causes of circadian misalignment being chronic sleep deprivation, altered eating patterns, and shift work (Sinturel et al. 2020).

Next to bodily internal processes, food intake is viewed as an external signalling system that provides timing cues for the synchronisation of biological rhythms (Han et al. 2022). It is thus not surprising that disordered eating behaviours may impact circadian rhythms of sleep and wakefulness through, for example, bingeing‐related suspended sleep timings, malnutrition‐related fatigue, or hunger‐related impaired sleep onset (Christensen and Short 2021). Thereby, cognitive–behavioural factors might interplay with physiological mechanisms, considering both disordered eating and disrupted, short sleep have been associated with altered hormone levels (Atalayer et al. 2013; Lin, Jiang, et al. 2020). Interestingly, ghrelin (the ‘appetite hormone’) is involved in the regulation of appetite *and* the sleep–wake cycle, with its secretion being influenced by sleep disruptions (García‐García et al. 2014). The secretion of ghrelin, in turn, increases the activity of the orexin system (neuropeptides released by the hypothalamus to regulate arousal states and energy balance), which has been shown to lead to elevated food seeking behaviours and heightened wakefulness in animal research (García‐García et al. 2014; Yamanaka et al. 2003). Consequently, both ghrelin and orexins are currently discussed as potential targets of therapeutic interventions for EDs to regulate food intake (Nunez‐Salces et al. 2021; Caldiroli et al. 2024; Mehr et al. 2021).

Considering these physiological links between the metabolic and circadian systems, a better understanding of the associations between disordered eating and sleep could provide novel insights into potential modifiable pathways to prevent the reciprocal exacerbation of both sleep and disordered eating concerns. To propose further research on the links between sleep and disordered eating specifically, De Young and Bottera (2022) recently argued for the investigation of a biobehavioural circadian model of restrictive eating and binge eating as transdiagnostic ED behaviours. As part of this model, De Young and Bottera illustrate various pathways through which disordered eating and circadian disruptions may be linked and reciprocally maintained. For example, in the case of binge eating, sleep restriction might impact individuals' appetite and desire for high‐fat foods, while disruptions in diurnal appetite rhythms may maintain circadian disruptions. Poor sleep may thereby not only maintain disordered eating symptoms, but also potentially cause and maintain other mental health difficulties (Zou et al. 2020; Scott et al. 2021). Thus, studying the relationship between different sleep and disordered eating outcomes could provide novel insights into innovative prevention and intervention approaches for EDs (Christensen and Short 2021; De Young and Bottera 2022) as well as co‐occurring mental health concerns.

When investigating the relationship between sleep and disordered eating, it is crucial to differentiate between symptom‐specific associations, as the particular mechanisms linking these constructs might differ for different behaviours. For instance, while Kim et al. (2010) identified sleep disturbances (such as difficulties falling asleep and midnight awakenings) primarily in patients with binge eating/purging‐subtype EDs, other research found sleep problems (mainly frequent and lengthy night awakenings) to be particularly pronounced in patients with anorexia nervosa (Allison et al. 2016). Likewise, research has specifically highlighted maladaptive sleep outcomes, such as longer waking times, fragmented nocturnal sleep, and altered sleep quality, from sustained fasting periods (cf. Lauer and Krieg 2004). Consequently, research investigating the association between sleep and disordered eating needs to consider differences between both subtype‐specific disordered eating behaviours and cognitions (e.g., restrictive eating behaviours, binge eating, and body image concerns) as well as different sleep indicators.

Similarly, sleep is a wide‐ranging construct, with different facets being explored across different studies. Sleep is commonly measured through a variety of methodologies that can be viewed as complementary, considering assessments of subjective self‐reported perceptions (e.g., questionnaires, sleep diaries) can differ from objective (polysomnographic or actigraphy‐based) measurements (Aili et al. 2017). Furthermore, sleep assessments can target a variety of sleep‐related experiences, with ‘sleep quality’ commonly being referred to as an overarching term that captures both (habitual) sleep behaviours (e.g., sleep duration) as well as individual nocturnal (e.g., sleep onset latency) or diurnal (e.g., excessive sleepiness) sleep‐related issues (Krystal and Edinger 2008). The absence of insomnia symptoms are thereby commonly seen as *part* of an individual's sleep quality (Fabbri et al. 2021). Thus, insomnia is generally being conceptualised as specific sleep‐related mental health difficulties, where the focus is on the emotional impact symptoms have on the individual (Bastien et al. 2001). Moreover, beyond sleep behaviours and their evaluation, individual preferences for circadian timings (e.g., people's chronotype) have received increasing attention as a potential transdiagnostic correlate of mental health issues (Taylor and Hasler 2018), including EDs (Menculini et al. 2019).

While previous reviews in this area have identified significant associations between an individuals' chronotype and their eating behaviours (Rodríguez‐Cortés et al. 2022; Teixeira et al. 2023), the prevalence of circadian disruptions and food intake‐related hormonal abnormalities in EDs (Menculini et al. 2019), sleep quality, circadian preferences, and sleep disorder symptoms in EDs (Degasperi et al. 2024), as well as associations between eating behaviours and poor sleep outcomes (Zerón‐Rugerio et al. 2023), no review has systematically explored associations between sleep and maladaptive eating behaviours in non‐clinical populations.

## 2 Research Aims

Overall, the present systematic review and meta‐analysis aimed to explore the relationship between disordered eating and sleep in non‐clinical populations, across all ages, considering symptom‐specific associations. To address knowledge gaps in our understanding of the mechanisms involved in this association (Christensen and Short 2021), the review additionally narratively explored moderators and mediators that have been identified for the association between disordered eating and sleep.

The current review will build on and extend previous reviews that have exclusively looked at clinical populations, by providing unique insights into the early stages of symptom overlap across specific behaviours and concerns. Focusing on non‐clinical populations provides the opportunity to investigate potential avenues for prevention. In doing so, findings from this review could inform psychoeducation and early intervention initiatives targeting the association between sleep problems and disordered eating, to increase awareness and health promotion strategies for those experiencing first signs of either symptom presentation.

## 3 Methods

### 3.1 Protocol and Registration

A protocol for this review was uploaded on PROSPERO (CRD42023427095) and the Open Science Framework (https://doi.org/10.17605/OSF.IO/JA9CN). An update to the original registration was uploaded in August 2023 to clarify exclusion criteria for articles with specialist populations.

### 3.2 Eligibility Criteria

To be included, studies needed to assess the association between sleep and disordered eating. The types of outcomes relevant for this review were defined broadly to allow for a better overview of available study findings. For sleep, this included objective and subjective measurements of sleep patterns (e.g., sleep duration, sleep timings), sleep disturbances (e.g., problems with falling asleep, waking up at night), sleep quality (both scales and single items assessing sleep quality), chronotype (individual differences in circadian timing), and sleep disorder symptoms (e.g., insomnia, hypersomnia). For disordered eating, this included general ED symptom assessments (other than diagnostic tools), restrictive eating behaviours (including intentions to lose weight), body image concerns, excessive exercise, bingeing/loss of control eating, purging behaviours, and night eating symptoms.

Both published and grey literature (theses, pre‐prints, and conference abstracts) were included. Studies with exclusively qualitative research designs or studies exclusively assessing clinical populations were excluded. Specialist populations that were recruited for their particular characteristics were excluded if these characteristics could impact either participants' eating or sleeping behaviours (e.g., those living with diabetes, cancer survivors, people with fatigue, and pregnant women). All types of reviews and case studies were excluded.

Only studies published in or after 2003 were included to ensure a focus on contemporary research. In line with the authors' language capabilities, studies published in English, German, Dutch, French, Spanish, and Portuguese were included.

### 3.3 Information Sources

Ovid (EMBASE, MEDLINE, and PsycINFO), ProQuest (ASSIA, Dissertations and Theses Global), EBSCO (CINAHL), PsyArXiv, Scopus, and Web of Science were searched for relevant articles on May 25, 2023 and updated on May 21, 2024.

### 3.4 Search Strategy

The final search strategy was developed in collaboration with a specialist local librarian and trialled in a pilot screening of titles and abstracts to optimise the search procedure. The final query string was defined as:

“disordered eating” or “eating disorder symptom*” or “eating patholog*” or “eating disorder psychopathology” or “eating disturbance*” or “eating disorder risk” or “ED risk” or “eating disorder prodrome” or “body image” or “body dissatisfaction” or “shape concern*” or “weight concern*” or “excessive exercis*” or dieting or “dietary restraint” or “restrictive eating” or “food restrict*” or “intention to lose weight” or bulimi* or binge* or overeating or “night* eating” or purg* or “emotional eat*” or “loss of control eat*”.

AND

“sleep* problem*” or “sleep* disturb*” or “sleep* efficiency” or “sleep* disorder*” or “sleep* wak* pattern*” or “sleep* onset” or insomnia* or parasomnia* or hypersomnia* or chronotyp* or circadian or “cyclic alternating pattern*” or “rapid eye movement*” or “social jetlag” or “sleep* fragmentation”.

In cases of missing or ambiguous information, study authors were contacted where possible up to two times via email or ResearchGate. Of all the contacted authors (*n* = 9), one got back to the research team with additional information.

### 3.5 Screening and Data Extraction

Covidence was used to conduct and coordinate the screening processes between reviewers. MCO screened all abstracts and titles, while a second reviewer (N.T.) screened a random sub‐sample (20%). The same procedure was applied for the full text review (M.C.O. reviewed 100%, N.T. 20%). Any discrepancies in screening decisions were discussed and resolved between M.C.O. and N.T. in collaboration with H.S. The data extraction and quality assessment of studies was conducted by M.C.O. (100%), and independently by M.C. (92%) and K.B. (8%) to ensure accuracy. For the updated literature search, M.C.O. reviewed all titles and abstracts, as well as full texts, while M.C. reviewed 20%. Finally, M.C.O. and S.M. extracted information from all additionally added studies and evaluated their quality according to the specified guidelines.

The following information was extracted: study characteristics (title, author(s), year of publication, country, study design, and publication type), sample characteristics (sample size, age, gender distribution, and ethnicity distribution), included outcome measures for sleep and disordered eating, included moderators/mediators (if applicable), reported statistical analyses and outcomes (including effect sizes) for the association between sleep and disordered eating, and authors' conclusion. The National Health, Lung, and Blood Institute's (NHLBI) quality assessment tool for observational cohort and cross‐sectional studies was applied to evaluate studies' quality. An overview of the quality assessment can be found in Table S3.

### 3.6 Data Synthesis

Given the wide range of study designs, concepts, and measures, both a meta‐analysis and narrative review were completed. Studies were included in the meta‐analysis if they (1) provided information on cross‐sectional associations between sleep and disordered eating, (2) measured predictors and outcomes with comparable assessment measures and study designs (i.e., cross‐sectional assessments measuring the same broad concept, which at times included different assessment formats, such as objective vs. self‐reported data), and if they (3) reported correlation coefficients/standardised regression coefficients, odds ratios, or standardised mean differences. The meta‐analyses were restricted to cross‐sectional findings, as there was not a sufficient number of longitudinal studies available to conduct meta‐analyses separately from cross‐sectional findings. Finally, meta‐analyses were only conducted if (4) at least three studies met all the above criteria (if one study explored associations in two different relevant populations, such as separate analyses and outcomes for adolescent and adult participants, they were considered separate analyses). A narrative synthesis was then conducted to summarise findings from any studies ineligible for inclusion in the meta‐analyses.

A meta‐analysis was conducted with all studies fulfilling the outlined eligibility criteria. The package ‘metafor’ (version 4.4‐0) from R (version 4.3.1) was used to specify random‐effects models for the respective constructs being explored (i.e., different types of sleep assessments, different disordered eating behaviours and cognitions). Cochrane's *Q* and *I* ^2^‐values were reported to assess study heterogeneity. *I* ^2^ values of 25%, 50%, and 70% were considered to represent small, moderate, and high levels of heterogeneity, respectively (Higgins et al. 2003). We considered a *p* value < 0.05 significant in all statistical analysis.

Due to the small number of studies included in all meta‐analyses models, publication bias and sensitivity analyses could not be conducted (Tang and Liu 2000). As most studies reported on correlation coefficients and regression coefficients, all meta‐analyses reported here were based on these values. Fisher's *z*‐transformed coefficients were included in meta‐analyses models and then back‐transformed to report pooled values.

## 4 Results

**FIGURE 1:**
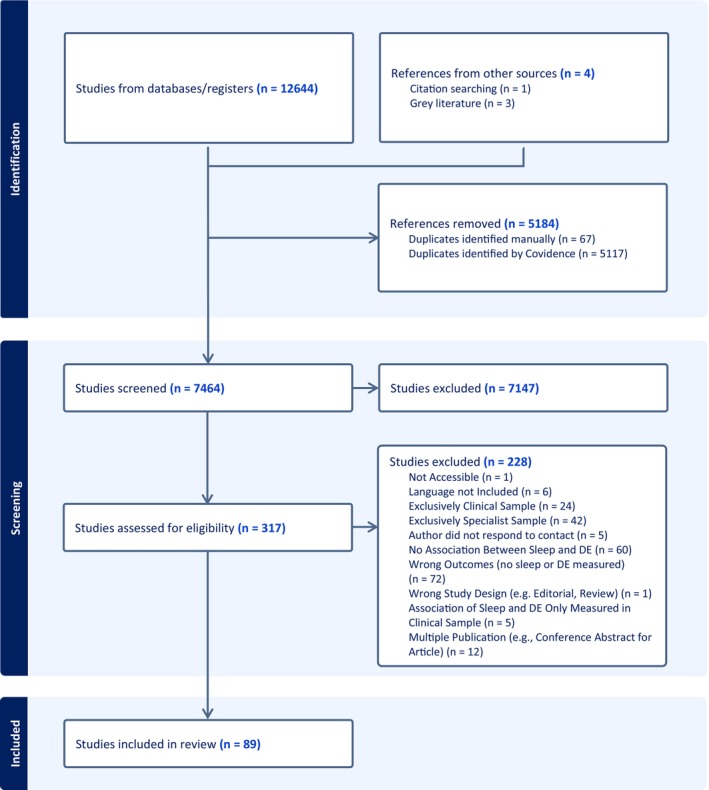
Flowchart illustrating screening process.

In total, 12,648 references were imported to Covidence for screening, and three further pre‐prints were identified on PsyArXiv. Of those, 5184 were duplicates. Thus, 7464 titles and abstracts were screened. This reviewing stage identified 317 studies as potentially eligible. After excluding *k* = 228 studies based on the reasons listed in Figure 1, *k* = 89 studies were included in the present systematic review, of which *k* = 33 were eligible to be included in the meta‐analyses. Agreement between reviewers was substantial to excellent for the abstract and title screening (agreement = 0.95, Cohen's Kappa = 0.64) and the full‐text review (agreement = 0.92, Cohen's Kappa = 0.84). Figure 1 depicts a flowchart of the screening process.

### 4.1 Study Characteristics

Table 1 outlines the study characteristics of all included studies. The final review included primarily published journal articles (*k* = 80, 90%) and studies that provided cross‐sectional findings of associations (*k* = 79, 88%). Most studies were conducted in the USA (*k* = 24, 27%), Turkey (*k =* 11, 12%), Italy (*k* = 7, 8%), and Iran (*k* = 7, 8%). Taken together, this review included information on *N* = 367,906 participants, most of which included children and adolescents at the baseline assessment (*k* = 30, 34%) or exclusively student populations (*k* = 29, 33%).

**TABLE 1:** Overview of study characteristics.

Author(s)	Country	Study design	Participant description	Number of participants (relevant for analysis)	Participants' ages (*M*, SD, range, if available)	Participants' gender distribution	Participants' ethnicity distribution	Sleep outcome	Disordered eating outcome
Ahorsu et al. (2023)	Iran	Cross‐sectional study	Adolescents	2088	*M* = 15.3, SD = 3.7, 13–18 years	*n* = 989 (47.4%) male, *n* = 1099 (52.6%) female	Not reported	Insomnia Severity Index (ISI): seven items, Pittsburgh Sleep Quality Index (PSQI): 19 items	Exercise Addiction Inventory—Youth Version (EAI‐Y): six items, Body Image Concern Inventory (BICI): 19 items, Eating Attitudes Tests (EAT‐26): 26 items
Akbari et al. (2022)	Iran	Cross‐sectional study	Adolescents 13–18 years and young adults	Adolescents: 562 Young adults: 745	Adolescents: *M* = 14.95, SD = 1.70; Adults: *M* = 26.19, SD = 7.42	Adolescents: 62.5% female; Adults: 60.5% female	Not reported	Insomnia Severity Index (ISI), Persian version: seven items	Exercise Addiction Inventory (EAI), Persian version: six items, Exercise Addiction Inventory Youth Version (EAI‐Y), Persian version: six items, Compulsive Eating Scale (CES), Persian version: eight items, Body Image Concern Inventory (BICI), Persian version: 19 items
Akram et al. (2021)	UK	Cross‐sectional study	Student sample and general population recruitment	728	*M* = 28.81, SD = 12.59, 19–75 years	89% female	Not reported	Sleep Condition Indicator (SCI): eight items, Sleep‐Associated Monitoring Index (SAMI): 10 items	Assessment of Body Image Cognitive Distortions Form A (ABCD): 18 items, Body Image Disturbance Questionnaire (BIDQ): seven items, Coping with Body Image Challenges Inventory (BICSI): 29 items
al Balushi and Carciofo (2023)	UK	Cross‐sectional study	Primarily student sample	272	*M* = 24.58, SD = 6.80, 18–55 years	*n* = 215 (79.04%) female; *n* = 55 (20.22%) male; *n* = 2 (0.01%) “other”	Not reported	Composite Scale of Morningness (CSM): 13 items	Binge Eating Scale (BES): 16 items
Aleksic et al. (2023)	Kosovo	Cross‐sectional study	Student sample	534	*M* = 21.7, SD = 2.6	*n* = 319 (59.7%) female; *n* = 215 (40.3%) male	Not reported	Socio‐epidemiologic questionnaire: time at which participants went to sleep, sleep quality (quite poor, poor, average, good, and quite good)	Night Eating Questionnaire: 14 items
Aloi et al. (2017)	Italy	Cross‐sectional study	Student sample	574	*M* = 21.4, SD = 2.3	*n* = 327 (57%) female; *n* = 247 (43%) male	Not reported	Pittsburgh Sleep Quality Index (PSQI)	Night Eating Questionnaire (NEQ): 14 items Binge Eating Scale (BES): 16 items Eating Disorder Examination Questionnaire (EDE‐Q): 22 items
Altan et al. (2018)	Turkey	Cross‐sectional study	High school students (9th, 10th, 11th, and 12th grade)	334	*M* = 15.72, SD = 0.62	Not reported	Not reported	Paediatric Daytime Sleepiness Scale: eight items	One question on body image: “What do you think about your body image?”
Arslan and Aydemir (2019)	Turkey	Cross‐sectional study	Healthcare professionals	535	*M* & SD not reported, range 18–65 years	*n* = 323 (60.4%) female; *n* = 212 (39.6%) male	Not reported	Pittsburgh Sleep Quality Index (PSQI)	Eating Attitudes Test (EAT): 40 items
Aspen et al. (2014)	United States	Cross‐sectional study	General population and those at high risk for an eating disorder	442	Overall: 18–25 years Control: *M* = 20.3, SD = 2.0 High risk: *M* = 20.6, SD = 2.0	100% female	Control: 56.3% White, 7.3% African American, 3.1% Hispanic/Latina, 29.2% Asian American, and 4.2% other High risk: 53.8% White, 9.8% African American, 10.4% Hispanic/Latino, 20.2% Asian American, and 4.2% other	Insomnia Severity Index (ISI): five‐items	Eating Disorder Examination‐Questionnaire (EDE‐Q): 39‐items
Babayan et al. (2018)	Iran	Cross‐sectional study	Middle‐aged married women	330	*M* and SD not reported, range 45–60 years	100% female	Not reported	Sleeping problems (no validated metric used), dichotomous variable—yes/no sleeping problem	Body Self‐Image Questionnaire: 23 items
Bahri et al. (2015)	Iran	Cross‐sectional study	Female high school students and their mothers	250	Not reported	100% female	Not reported	Insomnia questions from the General Health Questionnaire (GHQ‐28): one subscale on “distress and insomnia” (seven items)	Eating Attitudes Test (EAT‐26): 26 items Eating Disorder Inventory (EDI‐64): 64 items
Barnes et al. (2023)	United States	Cross‐sectional study	General population sample	648	*M* = 37.5, SD = 12.3, 18–80 years	*n* = 424 (65.4%) female	72.7% White non‐Hispanic, 9% Asian, 7.1% Black, 7.4% White Hispanic, 1.7% Native Alaskan/American Indian, 0.3% Native Hawaiian/Pacific Islander, 1.1% Bi‐/multiracial, and 0.8% not identifying with any of the categories	Pittsburgh Sleep Quality Index (PSQI): 19 items	Eating Loss of Control Scale (ELOCS): 18 items Eating Disorder Examination Questionnaire—Version 17 (EDE‐Q): reference to past 28 days
Bener et al. (2006)	Qatar	Cross‐sectional study	Adolescent boys	593	*M* & SD not reported, range 14–19 years	100% male	Not reported	Two items of the Self‐Reporting Questionnaire (SRQ‐20): sleeping badly and feeling tired all the time	Adolescent Dieting Scale (ADS): eight‐item scale, included were items on calorie counting, reducing food quantity and meal skipping
Berntzen et al. (2021)	Finland	Cross‐sectional study	Healthy young adult mono‐zygotic twins	111 (different sample sizes for different analyses)	*M* = 29.2, SD = 3.9, range not reported	50% male and female	Not reported	Actigraphy, worn for seven consecutive days (sleep duration, sleep efficiency, sleep onset latency, fragmentation index, sleep initiation, and waking) FIve sleep items: sleep duration, sleep need, sleep quality, morning tiredness, and daytime tiredness. Calculated sleep debt (sleep need—sleep duration) Basic Nordic Sleep Questionnaire (BNSQ): nine items Morningness–eveningness questionnaire (MEQ): 19 items	Binge Eating Scores (BES)
Birkeland et al. (2012)	Norway	Cohort study	School children to adults	1083	T1: *M* = 13.3; T2: *M* = 14.3; T3: *M* = 15.3; T4: *M* = 18.3; T5: *M* = 21.3; T6: *M* = 23.3; T7: *M* = 30.3	53% girls	Not reported	Bergen Insomnia Scale: six items	Body Image: four‐items (“I would like to change a good deal about my body”, “By and large, I am satisfied with my looks”, “I would like to change a good deal about my looks”, “By and large, I am satisfied with my body”)
Blouchou et al. (2024)	Greece and Cyprus	Cross‐sectional study	Greek and Cypriot adults	533	*M* = 26.6, SD = 9.3, range not reported	*n* = 356 (66.79%) female; *n* = 177 (33.21%) male	Not reported	Sleep, Circadian Rhythms, and Mood (SCRAM) questionnaire: 15 items	Night Eating Questionnaire (NEQ): 14 items
Borisenkov et al. (2020)	Russia (European North)	Cross‐sectional study	Secondary school and university students	2360	*M* = 17.9, SD = 4.6, 12–30 years	*n* = 1571 (66.6%) female; *n* = 789 (22.4%) male	Not reported	Munich Chronotype Questionnaire (MCTQ) Sleep onset on weekdays and free days, sleep onset latency, wake‐up time, sleep inertia, sleep duration, mean weekly sleep duration, and sleep efficiency	Yale Food Addiction Scale for Children (YFAS‐C) and YFAS, Russian version; symptom count (=sum of confirmed symptoms) and a dichotomous measure of food addiction
Bos et al. (2013)	Portugal	Longitudinal study	Student sample	T0: 870; T1: 592; T2: 305	*M* = 19.59, SD = 1.61, 17–25 years	*n* = 544 (62.53%) female; *n* = 326 (37.47%) male	Not reported	Two items: “I have difficulty falling asleep”, “I wake up many times during the night” Overall sleep disturbance score index (SDI): summing scores of both items (0–10 range). Four groups: good sleepers (“never”/“rarely” difficulties at baseline and follow‐up), persistent sleep difficulties (“often”/“very often”/“always” difficulties at baseline and follow‐up), onset sleep difficulties (not reporting sleep difficulties at baseline but either at T0 or T1 follow‐up), and remission sleep difficulties (sleep complaints at baseline but which decreased either at T0 or T1)	Eating Attitudes Test‐26 (EAT), Portuguese version: 33 items; High (1 SD above mean) versus low (1 SD below mean) EAT score group
Bruck and Astbury (2012)	Australia	Cohort study but only cross‐sectional used for analysis	Young adult women (aged 25–30)	5456	*M* = 27.14, SD = 1.45, 25–30 (range 24–30 years reported in abstract)	100% female	Not reported	One of the 21 “common problems” experienced during the past 12 months: “difficulty sleeping”; 4‐point scale (no, rarely, sometimes, and often); “often” defined as sleep problems. Dichotomous variable created as “often difficulty sleeping” versus “no/rarely difficulty sleeping”, anyone else omitted	Body weight dissatisfaction: One item: “How much would you to weight now?” (happy as I am, 1‐5 kg more, over 5 kg more, 1–5 kg less, 6–10 kg less, over 10 kg less), coded as “10 kg less” versus everything else (dichotomous)
Cakir et al. (2018)	Turkey	Cross‐sectional study	General population sample	3361	*M* = 38.7, SD = 12.7, range not reported	48.6% male, 51.4% female	Not reported	Pittsburgh Sleep Quality Index (PSQI)	Night Eating Disorder Scale (NEDS)
Çeçen and Guleken (2023)	Turkey	Cross‐sectional study	Females with and without obesity	54	*M* = 33.9, SD = 6.88, range not reported	100% female	Not reported	Biological Rhythms Interview of Assessment in Neuropsychiatry (BRIAN): 21 items	Yale Food Addiction Scale (YFAS): 27 items
Ceylan et al. (2024)	Turkey	Cross‐sectional study	Student sample	1035	*M* = 20.9, SD = 3.1, range not reported	65.3% female	Not reported	Morningness–Eveningness Questionnaire (MEQ): 19 items Social jetlag (calculated based on sleep timing)	Modified Yale Food Addiction Scale version 2.0 (mYFAS 2.0): 13 items, cut off for “moderate to severe food addiction” = at least four symptoms
Chardon et al. (2016)	United States	Cross‐sectional study	Children who attended paediatric appointment, aged 8–17 years	225	*M* = 12.39, SD = 2.7, 8–17 years	55% female	55% African American, 27% Caucasian, 10% bi‐racial, 3% Hispanic, 5% “other”	Sleep Disturbance Scale for Children (SDSC): 26 items (completed by parents) Paediatric Daytime Sleepiness Scale (PDSS): eight items (completed by young people)	Children's Eating Attitudes Test (ChEAT), 26‐items
Clark (2018)	United States	Cross‐sectional study (with four‐day sleep record data)	Student sample	639	*M* = 18.79, SD = 1.45, 16–21 years; Also reported: *M* = 18.77, SD = 1.39, 16–38 years	*n* = 352 (55.1%) male, *n* = 286 (44.8%) female; Later reported:*n* = 299 (46.7%) male, *n* = 340 (53.1%) female	Not reported	Sleep record (four consecutive days Thursday to Sunday) The Periodicity of Eating‐ Sleeping (POES‐FVS): bedtime, time arising, number of hours slept (POES Sleep sub‐score)	Binge Scale (BS): nine items, score < 8 = subclinical binge tendencies, score = 8–12 = potential binge problems, score > 12 = probable binge disorder; binge eaters defined as “yes” to the question “Do you binge eat?”
Cooper (2022)	United States	Longitudinal Study	Adolescents (aged 11–18 at beginning of study)	T0: 18,922; T1: 13,521; T2: 14,224	T1: *M* = 15.97, SD = 0.12, 11–18 years; T2: *M* = 16.47, SD = 0.11, 12–18 years; T3: *M* = 21.82, SD = 0.12, 18–26 years	49% female, 51% male	50.45% White (non‐Hispanic), 22.53% Black/African American (non‐Hispanic), 17.01% Hispanic/Latino, 7.08% Asian/Pacific Islander (non‐Hispanic), 1.84% American Indian/Native American, and 1.09% other	Sleep duration (average nightly hours of sleep for wave 1&2, sleep and wake times during week and non‐school days at wave 3, weighted by the number of week and weekend days and averaged over 7 days to calculate average hours per night) Single item for insomnia symptoms (ease of falling asleep and returning to sleep) at wave 1&2 (frequencies: never, rarely, occasionally, often, and every day); coded dichotomously (1 = insomnia symptoms occasionally, often, and every day) Single item for insufficient sleep at wave 1&2 (getting enough sleep); coded as 1 = sufficient sleep and 0 = insufficient sleep Single item for alertness at wave 3 (how often in past 7 days fell asleep when they should have been awake); frequencies (never, a few times, almost every day, and every day); coded dichotomously (1 = a few times, almost every day, and every day) Sleep health: composite sleep health (count of symptoms): insomnia symptoms, insufficient sleep, short sleep (< 7 h)	Those indicating trying to lose weight/stay the same weight were asked to identify engagement in particular restrictive and purging behaviours over past 7 days (wave 1–3): diet, exercise to lose weight, self‐induced vomiting, diet pill use, laxative use and/or other (each endorsed REPB = 1)
De Young et al. (2022)	United States	Cross‐sectional study (with actiwatch worn for 48 h)	Student sample	150	*M* = 19.55, SD = 2.5, 18–40 years	*n* = 116 (77.3%) female; *n* = 34 (22.7%) male	87.3% White, 4% Black, 1.3% Hispanic, 4% Asian, and 3.3% Native American	Morningness–Eveningness Questionnaire (MEQ): Scores 59–86 indicates morning sleep/waketime preference, scores 16–41 evening sleep/waketime preferences Pittsburgh Sleep Quality Index (PSQI) Naturalistic light exposure and sleep efficiency: actigraphy assessment (measuring sleep duration and sleep efficiency)	Eating Disorder Examination Questionnaire (EDE‐Q) Night Eating Questionnaire (NEQ): only subscales “Morning Anorexia” and “Evening Hyperphagia” used
Ee and Gan (2022)	Malaysia	Cross‐sectional study	Student sample	377	*M* = 21.85, SD = 1.59, range not reported	*n* = 264 (78.6%) female; *n* = 81 (21.4%) male	29.9% Malay, 70.1% non‐Malay; in total 63.2% Chinese	Pittsburgh Sleep Quality Index (PSQI): 19 items Morningness and Eveningness Questionnaire (MEQ): 19 items	Night Eating Questionnaire (NEQ): 14 items
Eid et al. (2022)	Australia	Longitudinal study	Normal‐, overweight and obese adults	T0: 161; T1: 155	*M* = 26.8, SD = 9.45, 18–65 years	*n* = 67 (42%) male; *n* = 94 (58%) female	Not reported	Pittsburgh Sleep Quality Index (PSQI): 19 items	Night Eating Questionnaire (NEQ): 14 items
Farhangi (2019)	Iran	Cross‐sectional study	Adolescent boys	80	*M* and SD not reported for whole population: 12–16 years	100% male	Not reported	Pittsburgh Sleep Quality Index (PSQI), Persian version: 19 items	Night Eating Questionnaire (NEQ): 14 items
Fernández Argüelles et al. (2022)	Spain	Cross‐sectional study	High school students	246	*M* = 13.28, SD = 0.57, 13–15 years	*n* = 130 (53%) boys; *n* = 116 (47%) girls	Not reported	Sleep Activity: ActiGraph accelerometers (seven complete days) and sleep diary: sleep efficiency (total sleep time divided by total time in bed, in %)	Body Image Discrepancy: Figure Rating Scale, adapted for Spanish context
Figueroa et al. (2024)	United States	Cross‐sectional study	General population sample	1993	*M* = 47.22, SD = 17.29, range not reported	*n* = 1023 (51.3%) female; *n* = 965 (48.4%) male; *n* = 5 (0.3%) non‐binary	63% White, 13.2% Black/African‐American, 1.3% Indigenous, Alaskan Native, Aleut, 15.5% Hispanic/Latina/o, 5.2% Asian/Asian American, 0.1% Native Hawaiian/Pacific Islander, 1.4% Biracial/Multiracial, and 0.4% other ethnicity	Sleep Disturbance bank form the PROMIS: six items (sleep quality and frequency of sleep disturbances over past week)	Eating Disorders Examination Questionnaire Short (EDE‐QS): 12 items
Fordsham et al. (2019)	United States	Cross‐sectional study	American adults recruited on Mechanical Turk	315	*M* = 34.8, SD = 10.55, 19–74 years	*n* = 183 (58.1%) male; *n* = 132 (41.9%) female	73.9% White, 8.6% Black, 6.7% Asian, 6.4% Hispanic/Latino, and 4.5% other	Morningness–Eveningness Questionnaire (MEQ): 19 items	Eating Attitudes Test—Short Version (EAT‐8): eight items
Gallant et al. (2013)	Canada	Cross‐sectional study	Student sample	3069	*M* = 27.9, SD = 10.2, range not reported	*n* = 2296 (74.8%) female; *n* = 773 (25.2%) male	Not reported	Sleep duration: “How many hours of sleep do you usually get per night?” (< 7 h = short sleeper)	Currently trying to lose weight (TLW): “Are you currently trying to lose weight?” Previously lost weight (PWL): “Have you ever deliberately lost weight (> 10 Ibs)?” Affirmative answers to both (RWL) Restrictive eating (from TFEQ): “Do you voluntarily stop eating before have emptied your plate to restrain your caloric intake?” often/always vs. never/rarely Overeating (from TFEQ): “Do you have trouble to stop eating before you have emptied your plate, even if you are no longer hungry?” > often/always vs. never/rarely
Gundogdu and Erdogdu Yildirim (2023)	Turkey	Cross‐sectional study	Adolescents aged 12–18 years	167	12–18 years; girls: *M* = 14.85, SD = 1.54; boys: *M* = 16.05, SD = 1.70	*n* = 93 (55.7%) female; *n* = 74 (44.3%) male	Not reported	Scale for outcomes in Parkinson's Disease (SCOPA) Sleep Scale: 12 items	The Night Eating Questionnaire (NEQ): 14 items
Hafstad et al. (2013)	Norway	Cohort study	Norwegian children and their mothers	T0: 913; T1: 777; T2: 727; T3: 373	Children's ages: T0: 1.5 years; T1: 2.5 years; T2: 4.5 years; T3: 16 years	100% female (parents); 58.7% female (children)	> 95% ethnic Norwegians	Sleep pattern in childhood (three items from the BCL = Behaviour Checklist): difficulties falling asleep at bedtime, nightly awakenings, unwillingness to sleep alone	Eating Attitudes Test (EAT‐12)
Hao et al. (2023)	China	Cross‐sectional study	Student sample	1258	*M* and SD not reported, 18–23 years	*n* = 630 (50.1%) male; *n* = 628 (49.9%) female	Not reported	Pittsburgh Sleep Quality Index (PSQI): 19 items	Stunkard visual graph to measure body dissatisfaction
Hasan et al. (2023)	United Arab Emirates	Cross‐sectional study	Student sample	552	*M* = 21.2, SD = 5.1, range not reported	*n* = 439 (79.5%) female; *n* = 113 (20.5%) male	*n* = 272 (49.3%) UAE nationals and GCC (Gulf Corporation Council) countries, *n* = 69 (12.5%) Arab non‐GCC, *n* = 211 (38.2%) non‐Arab	Pittsburgh Sleep Quality Index (PSQI): 19 items Morningness–Eveningness Questionnaire (MEQ): 19 items; score < =30 definite evening, score 31–41 moderate evening, score 59–69 moderate morning, score > =70 definite morning, categorised into three categories (definite and moderate merged)	Eating Attitude Test (EAT‐26): 26 items, score > 20 disturbed eating patterns
Hirai et al. (2022)	Japan	Cross‐sectional study	Highschool students under 18	398	Non‐ED: *M* = 14.9, SD = 1.7, 12–18 years ED symptoms: *M* = 16, SD = 1.9, 12–18 years	Non‐ED: *n* = 199 (52.2%) male, *n* = 182 (47.8%) female ED symptoms: *n* = 6 (35.3%) male, *n* = 11 (64.7%) female	Not reported	Athens Insomnia Scale (AIS), Japanese version: eight items	Eating Attitudes Test‐16: 26 items, cut‐off score > =20 indicates potential ED
Johnson (2020)	United States	Randomised controlled trial	Adult females	40	*M* = 25.08, SD = 8.42, range not reported	100% female	67.5% Asian, 12.5% White/Caucasian, 12.5% Black/African American, and 7.5% Hispanic/Latin	Sleep intervention: sleep deprived (50% of their typical reported sleep duration) vs. habitual sleep (100% of their reported sleep duration) Pittsburgh Sleep Quality Index (PSQI): 19 items Consensus Sleep Diary (CSD): 9 items	Eating Pathology Symptom Inventory (EPSI): 45 items Night Eating Questionnaire (NEQ): 14 items Eating Disorder Behaviour Form (EDBF): binge eating, overeating, loss of control eating, self‐induced vomiting, misuse of diuretics, exercise, skipping meals, fasting, urges to engage in these behaviours
Kandeger et al. (2018)	Turkey	Cross‐sectional study	Student sample	383	*M* = 21.1, SD = 0.1, 17–37 years	*n* = 230 (60.1%) female; *n* = 153 (39.9%) male	Not reported	Morningness–Eveningness Questionnaire (MEQ): 19 items; classification as morning type (score 59–86), evening type (16–41), and neither (42–58) Insomnia Severity Index (ISI): seven items	Night Eating Questionnaire (NEQ): 14 items, items 13, 15 and 16 not used for total score Eating Attitudes Test (EAT): 40 items, cut‐off = 30
Kandeger et al. (2019)	Turkey	Cross‐sectional study	Student sample	1323	*M* = 20.8, SD = 1.9, 16–33 years	*n* = 870 (65.8%) female; *n* = 453 (34.2%) male	Not reported	Morningness–Eveningness Questionnaire (MEQ): 19 items; classified into 3 categories (16–41 = evening, 42–58 = neither, 59–86 = morning) Insomnia Severity Index (ISI)	Yale Food Addiction Scale (YFAS): 25 items; in order for food addiction to be specified, participant had to meet = > 3 symptoms and indicate clinically significant impairment
Kiltie et al. (2024)	UK	Cross‐sectional study	Student sample	235	*M* = 21.45, SD = 5.02, range not reported	*n* = 178 female (75.7%)	Not reported	PROMIS sleep disturbance—short form: eight items (authors mentioned error in response scale) Number of hours spent out of bed (time to bed—time of waking)	Eating Pathology Symptoms Inventory (EPSI): 45 items
Krawitz (2011)	United States	Longitudinal study	Student sample	107	*M* = 22.36, SD = 6.73, 18–53 years	*n* = 90 (84.3%) female; *n* = 16 (14.8%) male; *n* = 1 (0.9%) other	*n* = 41 (34.5%) Caucasian, *n* = 27 (22.7%) African American, *n* = 26 (21.8%) Hispanic, *n* = 9 (7.6%) Asian, *n* = 1 (0.8%) Native Hawaiian/other pacific Islander, and missing = 12.6% (not reported)	Pittsburgh Sleep Quality Index (PSQI)	Bulimic Investigatory Test, Edinburgh (BITE): 33 items The Eating Disorders Examination Questionnaire (EDE‐Q)
Lauer et al. (2021)	United States	Cross‐sectional study	Female adolescents attending one of six middle schools in suburban school district in the southwestern USA	323	*M* = 12.33, SD = 0.04, range not reported	100% female	72% White	Insomnia Severity Index (ISI): seven items	Weight control behaviours: Intention about weight (“I am trying to lose weight”, “I am trying to gain weight”, “I am trying to stay the same weight”, “I am not trying to do anything about my weight in any way”)
Lee and Suh (2018)	South Korea	Cross‐sectional study	Student sample	172	*M* = 21.70, SD = 1.76, 18–29 years	100% female	Not reported	Disturbing Dream Nightmare Severity Index Questionnaire (DDNSI): five items Insomnia Severity Index (ISI): seven items	Night Eating Questionnaire (NEQ): 17 items
Lee et al. (2021)	United States	Cross‐sectional study	General population sample	1327	*M* = 47.7, SD = 17.2, range not reported	*n* = 669 (50.4%) female; *n* = 656 (49.4%) male; *n* = 2 (0.2%) non‐binary/other	64.1% White, 12.2% Black/African American, 1.4% Native American/Eskimo/Aleut, 15.1% Hispanic/Latino, 5.4% Asian/Asian American, 0.1% Native Hawaiian/Pacific Islander, 1.3% Biracial/Multiracial, and 0.4% Other	Six‐item sleep disturbance short form from the Patient‐Reported Outcomes Measurement Information System item bank (sleep quality over past week from very poor to very good and frequency of sleep disturbance in the past week from not at all to very much)	Eating Disorder Examination Questionnaire Short (EDE‐QS): 12 items (reference past 7 days)
Lew et al. (2020)	United States	Cross‐sectional study	Adolescents from the 2015 National Youth Risk Behaviour Survey	7130	Not reported	Matched sample: Short sleep duration (*n* = 3565): *n* = 1999 (56.1%) male, and *n* = 1566 (43.9%) female Sufficient sleep (*n* = 3565): *n* = 1978 (55.5%) male; *n* = 1587 (44.5%) female	Full sample: Short sleep: 46% non‐Hispanic White, 10.1% non‐Hispanic Black, 14.4% Hispanic, 23.4% non‐Hispanic mixed race, and 6.2% non‐Hispanic other race Sufficient sleep: 49.7% non‐Hispanic White, 7.7% non‐Hispanic Black, 16.4% Hispanic, 21.4% non‐Hispanic mixed race, and 4.7% non‐Hispanic other race Matched sample: Short sleep: 49.5% non‐Hispanic White, 7.7% non‐Hispanic Black, 16.2% Hispanic, 22% non‐Hispanic mixed race, and 4.7% non‐Hispanic other race Sufficient sleep: 49.7% non‐Hispanic White, 7.7% non‐Hispanic Black, 16.4% Hispanic, 21.4% non‐Hispanic mixed race, and 4.7% non‐Hispanic other race	Sleep duration, binary (< 8 h or > =8 h): “On an average school night, how many hours of sleep do you get?”	Body image: two dichotomous variables (whether adolescents felt they were the right weight and whether they were trying to either gain or lose weight)
Lin, Jiang, et al. (2020), Lin, Cheung, et al. (2020)	Iran	Longitudinal study but only cross‐sectional used for analysis	Adolescents	861	*M* = 15.9, SD = 3.2, 13–18 years	*n* = 372 (43.2%) male	Not reported	Insomnia Severity Index (ISI): seven items; different levels of insomnia (0–7 = absence, 8–14 = sub‐threshold, 15–21 = moderate, and 22–28 = severe)	Eating Attitude Test‐26 (EAT‐26): 26 items Yale Food Addiction Scale for Children (YFAS‐C); all converted into dichotomous items (symptom count scoring)
Liu et al. (2022)	China	Cross‐sectional study	High school students	7984	*M*, SD & range not reported	*n* = 3965 (49.7%) female; *n* = 4019 (50.3%) male	Not reported	Athens Insomnia Scale (AIS)	Eating Attitude Test (EAT‐19), Chinese version: 19 items
Lo et al. (2011)	China	Cross‐sectional study	High school youths in Hong Kong	20,677	*M* = 14.5, SD = 1.7, 11–18 years; boys: *M* = 14.5, SD = 1.83; girls: *M* = 14.5, SD = 1.79	*n* = 8728 (42.2%) male; *n* = 11,949 (57.8%) female	Boys: 74.2% born in Hong Kong, 25.8% born “elsewhere” Girls: 75.3% born in Hong Kong, 24.7% born “elsewhere”	Sleep problems as one indicator of “psychological distress” within “psychosocial health problems”; two items (adapted from Uppsala Sleep Inventory): “Do you find it hard to fall asleep or stay asleep?” and “Do you have nightmares?”; rated as dichotomous (never/seldom vs. sometimes/always)	Weight misperception (to measure body image disturbances): correct perception (normal weight + “just right” perception), misperceived fatness (normal weight + “too fat”/“fat”), misperceived thinness (normal weight + “too thin”/“thin”)
Lombardo et al. (2010)	Italy	Cross‐sectional study	Student sample	476	*M* = 23.22, SD = 2.85, range not reported	100% female	Not reported	Insomnia Severity Index (ISI)	Disordered Eating Questionnaire (DEQ): assessing the presence and intensity of eating restriction Contour Drawing Rating Scale (CDRS): pictorial measure of body dissatisfaction
Lombardo et al. (2013)	Italy	Non‐randomised experi‐mental study, but only cross‐sectional relevant	Student sample	39	*M* = 24.77, SD = 3.8, range not reported	100% female	Not reported	Sleep Disorder Questionnaire (SDQ): classification into three groups (Good Sleepers (GS) = no sleep problems, persistent insomnia (PI) = clinically significant symptoms consistent with diagnostic criteria, subthreshold insomnia (SI) = symptoms of insomnia with weekly frequency, persistence or consequences lower than those indicated by diagnostic criteria) Insomnia Severity Index (ISI)	Disordered Eating Questionnaire (DEQ): 24 items Eating Attitude Test (EAT‐26), Italian version: 26 items
Lombardo et al. (2014)	Italy	Cross‐sectional study	Students (colleagues, friends and acquaintances of the research team, approached at Sapienza University Rome)	1019	*M* = 24.38, SD = 4.12, 18–48 years	100% female	Not reported	Sleep Disorders Questionnaire (SDQ): three categories of sleep quality (good sleepers (GS) = no sleep problems, persistent syndromal insomnia (PI) = clinically significant symptoms of insomnia on basis of diagnostic criteria, subthreshold insomnia (SI) = symptoms of insomnia with frequency, persistence or consequences lower than those indicated by diagnostic criteria) The Insomnia Severity Index (ISI)	Disordered Eating Questionnaire (DEQ): 24 items
Lundgren et al. (2008)	United States	Cross‐sectional study	Non‐obese night eaters and non‐obese controls	41	Night eaters: *M* = 42, SD = 15.5; Controls: *M* = 36.5, SD = 12.1, ranges not reported	Night eaters: 84.2% female; Controls: 86.4% female	Night eaters: 94.7% Caucasian, 5.3% Hispanic/Latina, Controls: 81.8% Caucasian, 4.5% African American, and 13.6% Asian/Pacific Islander	24‐h sleep records (sleep and wake times) Morningness–Eveningness Scale Pittsburgh Sleep Questionnaire Epworth Sleepiness Scale (ESS)	Night Eating Questionnaire (NEQ): classified as NES‐positive if they reported evening hyperphagia (consumption of 25% or more of daily food intake after supper until final awakening next morning) and/or nocturnal awakening and ingestion of food three or more times per week
Manasse et al. (2022)	United States	Longitudinal study (EMA recordings)	Children with overweight/obesity aged 8–14	30	*M* = 11, SD = 1.89, 8–14 years	56.7% female	60% African American, 20% White, 13.3% Hispanic, 3.3% Asian, and 3.3% not identifying	Actigraphy (14 days), marking time beginning to fall asleep and time awakening: collected continuously and stored in 30 s epochs Sleep diary protocols: time in bed, time out of bed	Ratings for LOC eating (“While you were eating, did you feel a sense of loss of control?” “While you were eating, did you feel that you could not stop eating once you had started?” “While you were eating, did you feel like you could not resist eating?” “While you were eating, did you feel like a car without brakes, you just kept eating and eating?”). The four items assessing LOC eating were summed to form a total score (range = 4–20)
Mason and Heron (2016)	United States	Cohort study	General population sample	12,288	T1 (wave 3): 18–28 years; T2 (wave 4): 25–35 years	Not reported	Not reported	Sleep difficulties (assessed at T1): number of nights participants had trouble falling asleep in the past 4 weeks	Binge eating symptoms in the past week (assessed at baseline, T0): two items, rated as “yes” or “no” (“Have you eaten so much in a short period that you would have been embarrassed if others had seen you do it?” and “Have you been afraid to start eating because you thought you wouldn't be able to stop or control your eating?”) Weight perception (assessed ay T1): “What do you think about your weight” (very underweight, slightly underweight, about the right weight, slightly overweight, very overweight)
Matias et al. (2020)	Brazil	Cross‐sectional study	Brazilian adolescents	100,182	9th grade students (70.8% between 14 and 15 years)	51.7% female	Not reported	Adapted questions from the Global School‐Based Student Health Survey (GSHS) questionnaire: “How often were you unable to sleep at night because something really bothered you?” (yes = sometimes, mostly, always or no = never, rarely)	“Body weight dissatisfaction” (from the COMPAC study): self‐assessment scale, dichotomised as “satisfied” vs. “dissatisfied” Attitudes towards body weight: “What are you doing in relation to your body weight?” (trying to lose weight, trying to gain weight, trying to maintain weight) Both combined: “satisfied” (regardless of attitudes), “dissatisfied and not controlling weight”, “dissatisfied and trying to lose weight”, “dissatisfied and trying to gain weight”, “dissatisfied and trying to maintain weight”
Meule et al. (2014)	Germany	Cross‐sectional study	Student sample	729	*M* = 23.55, SD = 3.89, 18–47 years	*n* = 561 (77%) women	Not reported	Morningness–Eveningness Questionnaire‐reduced (rMEQ): five items	Night Eating Questionnaire (NEQ): 14‐items
Mori et al. (2009)	Japan	Cohort study but only cross‐sectional used for analysis	Junior high school first‐grade female pupils in the region (Toyama)	3939	All 12 years old	100% female	Not reported	Sleeping behaviours: waking‐up time, time of sleep, number of hours slept	Perceived body image: “Do you perceive yourself as thin, normal or fat?” in comparison to actual BMI: ‘underestimated group’ (underestimated weight), ‘normal group’ (normal weight) and ‘overestimated group’ (overestimated weight)
Nagata et al. (2021)	United States	Cohort study	General population sample (young adults)	12,082	*M* = 21.77, SD = 0.12; T1: 18–26 years; T2: 24–32 years	50.6% female, 49.4% male	69.4% White (non‐Hispanic), 15% Black/African American (non‐Hispanic), 11.4% Hispanic/Latina, 3% Asian/Pacific Islander (non‐Hispanic), 0.5% American Indian/Native American, and 0.7% other	Proxies of sleep disturbances: trouble falling asleep (“Over the past 4 weeks, how often did you have trouble falling asleep?”), trouble staying asleep (“Over the past 4 weeks, how often did you have trouble staying asleep through the night? For example, you woke up several times at night or woke up earlier than you planned to?”); 4 response options for both (never in past 4 weeks, less than once a week, 1 or 2 times a week, 3 or 4 times a week, 5 or more times a week); at baseline, both questions were combined	Eating behaviour: those indicating wanting to lose weight or stay the same weight were asked “Which of the following things did you do during the past 7 days to lose weight or stay the same weight?” (fasting/skipping meals, making yourself throw up, laxatives, diuretics, taking weight loss pills), of which the first was defined as restrictive eating behaviour, the rest compensatory Overeating or loss of control eating: present when individuals self‐reported that they have “eaten so much in a short period of time that they would have been embarrassed if others had seen them do it” or who indicated they had “been afraid to start eating because they thought they wouldn't be able to stop or control their eating” in the past 7 days
Natale et al. (2008)	Italy	Cross‐sectional study	Controls for comparison with females in eating disorder treatment centre	124	Controls: *M* = 31.98, SD = 10.31, 18–44 years	100% female	Not reported	Reduced version of Morningness–Eveningness Questionnaire (MEQr): five items, three labels (4–10 = evening, 11–18 = intermediate, and 19–25 = morning)	Eating Disorder Inventory (EDI‐2): 91 questions
Nolan and Geliebter (2016)	United States	Cross‐sectional study	University students and general (older) adult population	Students: 254 community: 244	Students: *M* = 18.7, SD = 0.1 Community: *M* = 44.5, SD = 0.9, ranges not reported	Students: 63.1% female, 34.9% male Community: 59% female, 41% male	Not reported	Pittsburgh Sleep Quality Scale (PSQI): 19 items	Night Eating Questionnaire (NEQ): 14 items Yale Food Addiction Scale (YFAS)
Nolan and Geliebter (2019)	Online and United States	Cross‐sectional study	Community members (online) and university students	Community members: 468 university students: 254	Student: *M* = 18.7, SEM = 0.1 Community: *M* = 42.9, SEM = 0.6, range not reported	Students: 63.1% female, Community: 55.8% female	Approximately 80% White	Pittsburgh Sleep Quality Index (PSQI): 19 items	Night Eating Diagnostic Questionnaire (NEDQ), symptom checklist: 22 items
Park et al. (2020)	South Korea	Cross‐sectional study	Women aged 19 and above	9288	> 20 years	100% female	Not reported	Appropriate sleep (7‐8 h) measured as part of general health‐related behaviour assessment	Subjective body image perception: “What do you think of your body image?” (severely thin/slightly thin, average/normal, slightly overweight/severely overweight); adjusted by BMI (BIOP = body image over perception, BICP = body image correct perception, BIUP = body image under perception)
Parker et al. (2020, 2022)	United States	Cross‐sectional study (with 14‐day EMA assessment)	Youth (8–17) from the Children's Growth and Behaviour Study	48	Article: *M* = 12.88, SD = 2.69, range not reported	Article: *n* = 33 (68.8%) female; *n* = 15 (31.2%) male	50% White, 14.6% Non‐Hispanic Black or African American, 22.9% Asian, 8.3% Multiracial, and 4.2% unknown Ethnicity: 85.4% non‐Hispanic, 12.5% Hispanic, and 2.1% unknown	Actigraphy monitors to assess sleep (within‐person shifts in nightly sleep duration (h/night), bedtime, waketime, and midpoint were computed using the absolute difference between a person's duration/time of the previous night's sleep and their 2‐week average sleep duration/time)	Ecological Momentary Assessment (EMA) for loss of control eating (LOC): six items (e.g., I felt a sense of loss of control, I could not stop eating once I started); severity computed by averaging items within persons and within days LOC‐Severity: When an eating episode was reported, participants rated the degree to which they experiences LOC‐eating during that snack/meal, using adapted items from the EDE: “How much did you lose control during this eating episode?”, “Did you feel that you could not keep yourself from eating?”, “Did you feel that you could not stop eating once you started?”, “During the eating episode you just finished, how much did you feel a sense of loss of control?”, “How upset or distressed are you about how much you just ate?”, “How much did you feel driven to eat?”
Prieto et al. (2012)	Spain	Cross‐sectional study	Adolescents (12–16)	1600	*M* = 14.13, SD = 1.47, 12–16 years	49.6% girls	Not reported	Morningsness‐Eveningness Scale for Children (MESC): 10 items	Veçú et santé perçue de l'adolescent (VSP‐A): body image items (39 items)
Ramos et al. (2023)	Brazil	Cross‐sectional study (using PeNSE 2015)	Adolescents	2439	54.4% aged between 15 and 19 years, 45.6% aged between 11 and 14 years	*n* = 1259 (51.6%) female; *n* = 1180 (48.4%) male	71.1% Non‐White, 28.9% White	Insomnia: sporadic (never/sometimes/rarely), yes (often/always)	Extreme Weight Loss Behaviour (EWLB): “Did you vomit or take laxatives to lose weight or avoid gaining weight?” (yes, no), “Have you taken any medicine, formula, or other weight loss product without medical follow‐up?” (yes, no), at least of these questions affirmed was considered presence of EWLB Body Image Satisfaction: satisfied (satisfied/very satisfied), indifferent, dissatisfied (dissatisfied/very dissatisfied) Attitude towards weight: no attitude, trying to lose or maintain weight, trying to gain weight
Reche‐Garcia et al. (2018)	Spain	Cross‐sectional study	Student sample	240	*M* = 21.1, SD = 4.2, 18–43 years	84% male, 16.3% female (reported in text, slightly different in tables: 86.7% male, 16.3% female)	Not reported	Athens Insomnia Scale (EAI‐8): eight items	Exercise Dependence (EDS‐R): 21 items, divided into risk of dependency (RD = scores > 5 in three criterions), symptomatic but independent (SID = scores of 3–4 in three or more criterions or obtain scores of 5–6 combined with scores of 3–4 in three criterions, without reaching the requirements to be included in the RD group), and asymptomatic independent (AID = minimum score of one to two in at least three criterions, without reaching the requirements of incorporation in the SID group)
Reichborn‐Kjennerud et al. (2004)	Norway	Longitudinal population‐based twin panel but only cross sectional used for analysis	Twins in Norway (population‐based sample)	7831	*M* = 25.52, SD = 3.70, 18–31 years Men no BE: *M* = 25.60, SD = 3.67 Men BE: *M* = 26.16, SD = 3.71 Women no BE: *M* = 25.47, SD = 3.72 Women BE: *M* = 25.21, SD = 3.71	*n* = 3352 (42.8%) male, *n* = 4479 (57.2%) female	Not reported	Five‐item version (SCL‐5) of the Hopkins Symptom Check List (SCL)‐25 was used to assess symptoms of anxiety and depression = screening for insomnia, sleep medication use	Binge eating: “Have you lost control while eating and were unable to stop before you had eaten too much?” (at least twice a week, 1–4 times a month, seldom or never) Inappropriate compensatory behaviours: “Have you used (1) vomiting or (2) laxatives or (3) fasting or (4) excessive physical exercise to control your weight?” (at least twice a week, 1–4 times a month, seldom or never) Binge eating in the absence of compensatory behaviours = a feeling of loss of control at least 2 times a week and the absence of regular use of any of the above‐mentioned inappropriate compensatory behaviours.
Riccobono et al. (2019)	Italy	Cross‐sectional study	High school students	301	*M* = 17.64, SD = 1.3, 15–19 years	*n* = 181 (60.1%) female; *n* = 120 (39.9%) male	Not reported	Morningness–Eveningness Questionnaire (MEQ): 19 items; three types (morning = 59–86, intermediate = 42–58, and evening = 16–41)	Night Eating Questionnaire (NEQ): 15 items
Riccobono et al. (2020)	Italy	Cross‐sectional study	Student sample	1136	*M* = 25.97, SD = 10.78, range not reported	*n* = 360 (31.7%) male; *n* = 774 (68.1%) female	Not reported	Morningness–Eveningness Questionnaire (MEQ), Italian version: 19 items, divided into morning type (score 59–86), intermediate type (score 42–58), and evening type (score 16–41)	Night Eating Questionnaire (NEQ), Italian version: 15 items
Richardson et al. (2025)	Australia	Cohort study	Adolescents (data collected as part of the Risks to Adolescent Wellbeing (RAW) Project)	528	T0: *M* = 11.19, SD = 0.55, 10–12 years; T1: *M* = 12.19, SD = 0.53, 11–13 years; T2: *M* = 13.20, SD = 0.53, 12–14 years; T3: *M* = 14.25, SD = 0.56, 13–16 years; T4: *M* = 15.24, SD = 0.57, 14–17 years; T5: *M* = 16.24, SD = 0.56, 15–18 years	T0: 51% male; T1: 52% male; T2: 52% male; T3: 52% male; T4: 51% male; T5: 52% male	82% White background	Children's Morningness–Eveningness Scale (MESC): 10 items School‐night sleep duration: “How many hours sleep do you usually get each night on a school night?” (pre‐filled drop down from 0 to 12) Paediatric Daytime Sleepiness Scale (PDSS): eight items, higher scores reflect greater daytime sleepiness (for longitudinal analyses reverse‐scored)	Children's Eating Attitude Test (ChEAT): 26 items
Rosenbaum et al. (2023)	United States	Cross‐sectional study	Female undergraduates	269	*M* = 19.53, SD = 3.26, range not reported	100% female	20.1% African American/Black, 20.4% Asian American/Asian/Pacific Islander, 16% Latina/Hispanic, 0.4% Native American/American Indian, 5.9% Multiracial, 33.8% White, and 3% other	“In the past 7 days, on average, how many hours of sleep did you get per night?” (open‐ended)	Body Appreciation Scale 2 (BAS‐2): 10 items Appearance Evaluation (seven items) and Appearance Orientation (12 items) subscales of the Multidimensional Body Self Relations Questionnaire (MBSRQ)
Sahlan et al. (2023)	Iran	Cross‐sectional study	Student sample	1043	*M* = 20.77, SD = 2.08, 18–27 years	88.1% women	Not reported	Farsi‐Insomnia Severity Index (F‐ISI)	Farsi‐Eating Disorder Examination Questionnaire – 6th Edition (F‐EDE‐Q): combined four subscales to examine global ED symptoms over the past 28 days; an additional item on binge eating (having loss of control over eating and consuming a large amount of food); self‐induced vomiting and laxative misuse merged
Schmidt and Randler (2010)	Germany	Cross‐sectional study	Secondary school adolescent girls	284	*M* = 14.1, SD = 1.2, 12–17 years	100% female	Not reported	Composite Scale of Morningness (CSM): 13 items; scores range from 13 (extreme eveningness) to 55 (extreme morningness) Rising times and bedtimes during weekdays and weekends Parental monitoring of bedtimes (“My parents set by bedtime”), during week and weekends	Eating Disorder Inventory‐2 (EDI‐2), German version: 91 questions, capturing 11 dimensions; focus on 3 subscales (drive for thinness, bulimia, body dissatisfaction)
Seigel et al. (2004)	Sweden	Cross‐sectional study	Young adult females	726	*M* = 20.2, SD = 1.8, 18–23 years	100% female	Not reported	Items taken from the Uppsala Sleep Inventory but used measure constructed for this study: Insomnia score: “How severe are your problems with… difficulties falling asleep after going to bed, frequent awakening during the night, awakening too early in the morning, not feeling sufficiently rested by sleep?” (no, small, moderate, severe, and very severe); cut‐off = “severe”	Body Image and Eating: “How many times have you attempted to reduce your weight?” (never, 1–5 times, 6–10 times, > 10 times); cut‐off = “6–10 attempts” “How often do you…fear gaining weight, feel overweight, feel dissatisfied with your body, eat large amounts of food with some loss of self‐control, feel impulses to vomit after meals?” (never, seldom, sometimes, fairly often, very often); cut‐off = “rather often”
Soares et al. (2011)	Portugal	Cross‐sectional study	Student sample	870	Female: *M* = 19.5, SD = 1.56, 17–25 years Male: *M* = 19.8, SD = 1.68, 17–25 years	62.5% female, 37.5% male	Not reported	Two items: “I have difficulty falling asleep” (DIS) and “I wake up many times during the night” (DMS); rated on 6‐point scale (never to always); Sleep disturbance index (SDI) calculated from the sum of DIS and DMS item scores	Eating Attitudes Test‐40 (EAT‐40): 40 items
Suna and Ayaz (2022)	Turkey	Cross‐sectional study	Student sample	568	*M* = 20.32, SD = 1.61, 18–25 years	21.3% male, 78.7% female	Not reported	Pittsburgh Sleep Quality Index (PSQI), Turkish version: score < =5 considered “good” sleep quality, > 5 considered “poor” sleep quality	The Eating Attitude Test (EAT‐26), Turkish version: cut‐off score = 20 Night Eating Questionnaire (NEQ): 14 items
Taut et al. (2018)	Romania	Cross‐sectional study	11–15‐year‐old school children	5404	*M* = 13.23, SD = 1.65, 11–15 years (different age ranges reported in abstract)	50.6% girls	Not reported	1 item on difficulties in getting to sleep (part of health complaints): about every day, more than once a week, almost every week, almost every month, rarely or never (absent = rarely/never, present = everything else)	Body weight dissatisfaction: “Do you think your body is…” (much too thin, too thin, about the right size, too fat, much too fat) Unhealthy weight control: “Which of the following things have you done in the last 12 months in order to lose weight” (skipping meals, smoking, vomiting, using pills, and restricting their diet to one of more foods); answered as yes/no
Tholin et al. (2009)	Sweden	Cross‐sectional study (population based twin study)	Swedish twins	6208	Overall: 20–47 years; men: *M* = 34.7, SD = 7.7; women: M = 34.5, SD = 7.6	*n* = 9743 (44.8%) male; *n* = 11,998 (55.2%) female	Not reported	Sleep‐related problems: “Have you had difficulty falling asleep (during the past 3 months)?”, “Have you had feelings of not having had enough sleep on awakening (during the last 6 months)?”, “Have you had disturbed or restless sleep (during the last 6 months)?”; sleep problem = “usually”/“always”	Night eating (broad): awakening with food intake during the night at least once a week and/or 25% of daily food intake after the evening meal Night eating (narrow): awakening with food intake at least once a week and/or < =50% of daily food intake after the evening meal Night eating: “How often do you get up at night to eat?” (never, once or twice, weekly, nightly, don't know/wish not to answer) and “What proportion of your daily food intake takes place after the evening meal?” (0, 1–24, 25–49, 50–74, 75–100, and don't know/wish not to answer) Binge eating: “Have you ever had binges when you ate what most people would regard as an unusually large amount of food in a short period of time?” (yes, no, and don't know/refuse) and “When you were having eating binges, did you feel that your eating was out of control?” (not at all, slightly, moderately, very much, extremely, and don't know/don't wish to answer); binge eaters = “yes” and “very much”/“extremely”
Trace et al. (2012)	Sweden	Cross‐sectional study	Twins born between 1959 and 1985	3790	BE women: *M* = 31, SD = 7.4 No BE women: *M* = 33.4, SD = 7.7, ranges not reported	100% female	Not reported	Items on sleep habits and problems: “Do you usually take a nap at least every second day?” (yes, no), “Do you think you get enough sleep?” (yes, definitely enough, yes, mostly enough, no, a bit too little, no, clearly not enough, no, and far from enough), “How do you think you sleep on the whole?” (very well, pretty well, neither poorly nor well, pretty poorly, and very poorly), “Try to determine to what degree you are a morning person or a night person” (definitely a morning person, to some degree a morning person, to some degree a night person, definitely a night person); complaints during past 3 months: “Problem falling asleep?”, “Sleepy during work or free time?”; complaints during past 6 months: “Waking up too early and not being able to sleep again?”, “Feeling of not having enough sleep on awakening?”, “Disturbed or uneasy sleep”, both assessed with five categories (never, seldom, sometimes, and usually, always)	Lifetime history of binge eating (based on SCID), two items: “Have you ever had eating binges when you ate what most people would regard as an unusually large amount of food in a short period of time?” (yes, no, and do not know/refuse) and “When you were having eating binges, did you feel your eating was out of control?” (not at all, slightly, somewhat, very much, extremely, do not know/refuse); BE group defined as yes (first item) and slightly/somewhat/very much/extremely (second item)
Özata Uyar et al. (2023)	Turkey	Cross‐sectional study	General population sample	362	*M* = 26.8, SD = 9 19–60 years	*n* = 185 (51.1%) female; *n* = 177 (48.9%) male	Not reported	Morningness–Eveningness Scale (MEQ): 19 items, higher scores reflect more morning preference, scores 16–41 “evening type”, 42–58 “intermediate type”, and 59–86 “morning type”	Night Eating Questionnaire (NEQ): 14 items, cut‐off score > =25 and > =30 (increased specificity)
Vrabec et al. (2022)	United States	Cross‐sectional study	Student sample	372	*M* = 19.47, SD = 1.75, 18–25 years	*n* = 238 (64%) female; *n* = 134 (36%) male	Race: 1.6% American Indian/Alaskan Native/Native Hawaiian, 21.8% Asian/Asian American, 6.2% Biracial/Multiracial, 10.5% Black/African American, 60.2% White (incl. Middle East) Ethnicity: 9.4% Hispanic	Social jetlag: bedtimes and waketimes on both weekends and weekdays (mid‐sleep time was recorded by calculating the time at the midpoint of the sleeping period (MST); difference between MST on weekdays and weekends was calculated) Morningness–Eveningness Questionnaire (MEQ): 19 items, evening type = 41 or below, intermediate = 42–58, morning = 59 or above PROMIS Paediatric Sleep‐Related Impairment Short Form 8a: eight items Sleep quantity: calculated from self‐report bedtime and waketimes on weekends and weekdays	Loss of Control over Eating Scale (LOCES): seven items
Walker et al. (2018)	United States	Cross‐sectional study	Student sample	452	*M* & SD not reported, range 18–25 years	66% female	59% White	Sleep was measured via two self‐report items asking about satisfaction with current sleep pattern (i.e., sleep quality) and number of minutes it typically took to fall asleep over the past month (i.e., sleep onset latency, SOL).	Restrictive eating was measured using an item from a national youth risk survey that asked about restriction of food, calories, or fat content in food intake with the intent of weight loss over the past 30 days.
White et al. (2024)	United States	Cross‐sectional study	Adolescents with sleep concerns	106	*M* = 14.44, SD = 1.69, 12–18 years	*n* = 42 (47.2%) female; *n* = 47 (52.8%) male	16.90% White, 4.5% Black, 55.10% Hispanic/Latino, 2.2% Asian, 1.1% Native American, 1.1% Middle Eastern, 16.9% Biracial/multiracial, and 2.2% other ethnicities	Time‐in‐bed: typical weekday bedtimes and wake times (average school night) Paediatric Insomnia Severity Index (PISI): six items, reference to past week	Body Dissatisfaction Scale (BDS): 9 computer‐generated female and male body images, body dissatisfaction rated as difference between ideal and actual body ratings
Wroblevski et al. (2022)	Brazil	Cross‐sectional study	High school students	97,036	*M* & SD not reported, range: 13–15 years	47.8% male, 52.2% female	Not reported	The variable “insomnia” has a value of 1 if the student answered that they always or almost always have trouble sleeping because something worries them and zero if they never, rarely or sometimes have trouble sleeping.	Body dissatisfaction: “How do you feel about your body?”. The answers are: very satisfied, satisfied, indifferent, dissatisfied and very dissatisfied. The “treatment” group included students who answered that they were dissatisfied or very dissatisfied with their body. The “treatment” group is defined by students who reported some level of dissatisfaction with their own body image and the control group is made up of students who did not report being dissatisfied with their body image.
Wu et al. (2021)	Tibet	Cross‐sectional study	Student sample	4325	*M* = 19.9, SD = 1.3, range not reported	*n* = 1668 (38.6%) male; *n* = 2657 (61.4%) female	40.3% Han, 57.1% Tibetan, 2.6% other	Pittsburgh Sleep Quality Index (PSQI) Chinese version	Eating Attitude Test‐26 (EAT‐26): 26 items
Yeh and Brown (2014)	Australia	Cross‐sectional study	General population sample	330	*M* = 27.42, SD = 10.36, 18–87 years	*n* = 107 (32.4%) male; *n* = 223 (67.6%) female	Not reported	Pittsburgh Sleep Quality Index (PSQI): 19 items; score > 5 indicates moderate to severe sleep difficulties (differentiates poor and good sleepers)	Binge Eating Scale (BES): 16 items, score > =27 considered to be binge eaters Night Eating Questionnaire (NEQ): 14 items, score = 25 identifies possible NES cases
Yilmaz Yavuz and Altinsoy (2022)	Turkey	Cross‐sectional study	Academics based in Turkey's Eastern Black Sea Region	221	*M* = 33.45, SD = 9.65, 24–67 years	*n* = 126 (57%) male; *n* = 95 (43%) female	Not reported	Morningness–Eveningness Questionnaire (MEQ): 19 items, divided into morning type (score: 59–86), intermediate type (42–58), and evening type (16–41)	Night Eating Questionnaire (NEQ): sore > = 25 considered NES

In terms of sleep assessment, only *k* = 5 (6%) studies used an objective actigraphy measure to assess sleep; all other studies used exclusively self‐assessments. While sleep assessments via questionnaire most commonly included a version of the Pittsburgh Sleep Quality Index (*k* = 19, 21%), the Morningness–Eveningness Questionnaire (*k* = 15, 17%), and the Insomnia Severity Index (*k* = 13, 15%), individual sleep habits (e.g., sleep duration) and sleep problems (e.g., problems with falling asleep or waking up at night) were also commonly assessed. In regard to disordered eating, the assessment methods were more varied; however, most studies reported findings on night eating using the Night Eating Questionnaire (*k* = 20, 23%) and general eating disorder symptoms using a version of the Eating Attitudes Test (*k* = 17, 19%) or the Eating Disorder Examination Questionnaire (*k* = 8, 9%). An overview of all study methodologies and relevant study findings is provided in Tables 1 and S1.

### 4.2 Quality Assessment

The vast majority of studies included in this review outlined clear research questions, and clearly defined utilised variables. *k* = 35 studies (39%) used non‐validated items (e.g., items measuring behavioural proxies, different language versions of questionnaires where validation was unclear) to assess either or both disordered eating and sleep. Of these, 26 (29% of total studies) used exclusively single‐item assessments (e.g., “I have difficulty falling asleep”, “What do you think about your body image?”) for one or both constructs. Cohort studies commonly used single‐item assessments, likely due to large assessment batteries. Overall, most studies demonstrated acceptable quality, even though the majority of studies utilised a cross‐sectional and only one study used an experimental design (Johnson 2020). Two studies were considered poor quality (Babayan et al. 2018; Bahri et al. 2015), as assessment approaches and reporting were unclear.

A full overview of studies' quality assessment is available in Table S2, and quality criteria were considered within the narrative review below.

### 4.3 Meta‐Analyses and Narrative Synthesis

**FIGURE 2:**
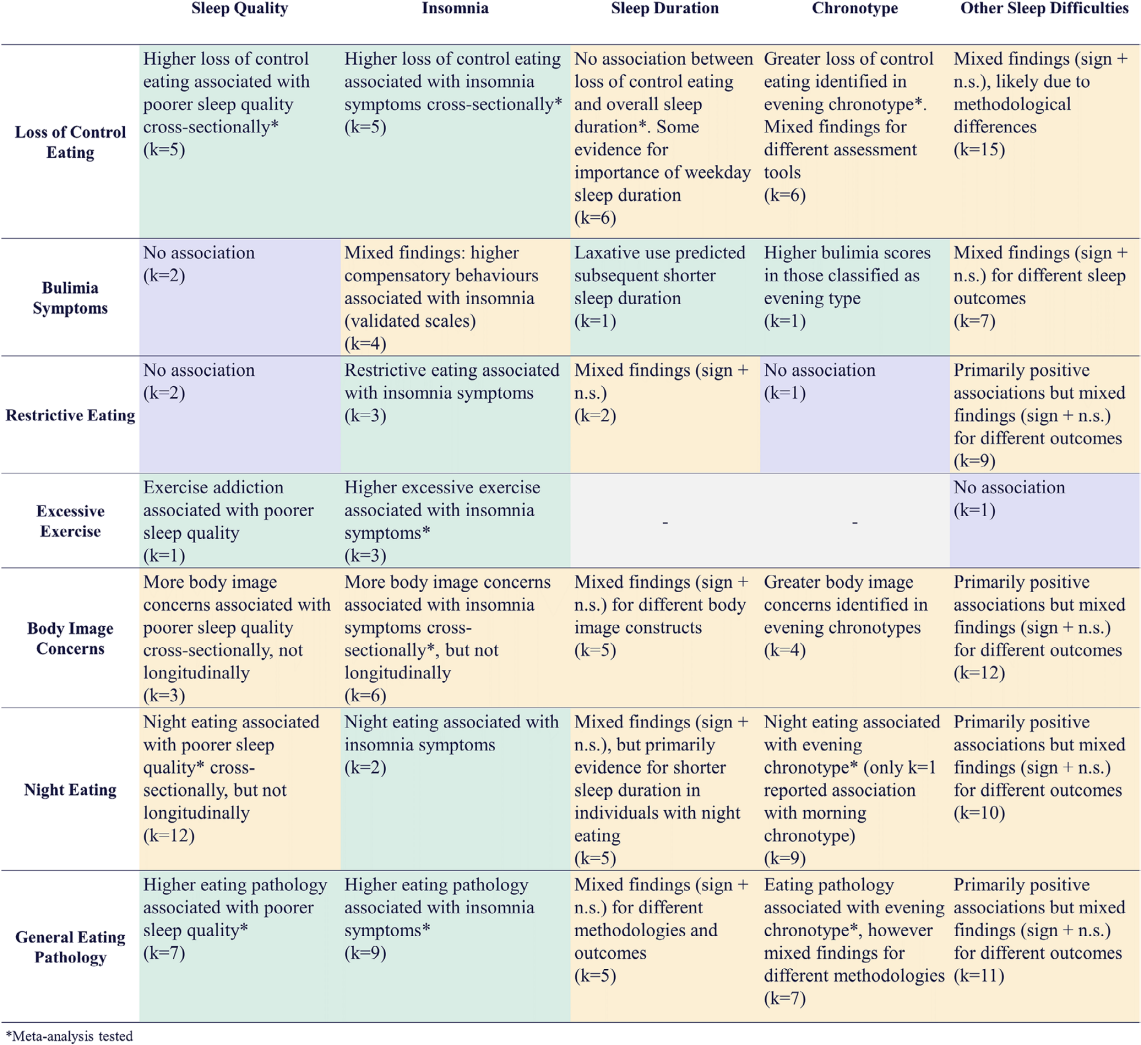
Overview of associations between sleep and disordered eating.

To aid in synthesis and to highlight subtype‐specific research gaps in the current literature, disordered eating concepts were grouped into broadly similar constructs. The heterogeneity in assessments for both disordered eating and sleep was acknowledged by providing additional information on methodological approaches in the narrative review, Tables 1 and S1. Each section will first outline findings from meta‐analyses (if obtainable), and then narratively discuss further study findings that could not be included in meta‐analyses. Figure 2 provides a broad overview of all associations synthesised in this review.

#### 4.3.1 Binge Eating, Loss of Control Eating, and Food Addiction

In the following we have synthesised all information on what was broadly termed “loss of control eating”, including concepts such as binge eating and food addiction, acknowledging that, while these concepts are distinct, they share important common characteristics (e.g., eating more than intended/desired).

##### 4.3.1.1 Sleep Quality

**FIGURE 3:**
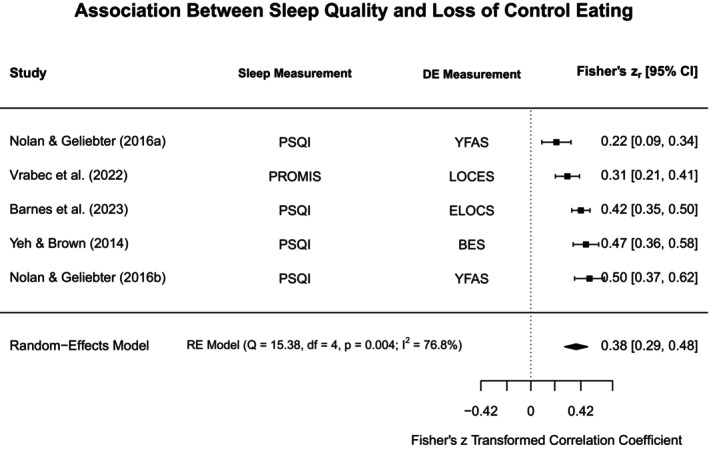
Meta‐analysis illustrating the association between sleep quality and loss of control eating. BES = Binge Eating Scale, ELOCS = Eating Loss of Control Scale, LOCES = Loss of Control Eating Scale, PSQI = Pittsburgh Sleep Quality Index, PROMIS = Patient‐Reported Outcomes Measurement Information System, YFAS = Yale Food Addiction Scale.

Five analyses from four studies met eligibility criteria for being included in the meta‐analysis assessing the association between sleep quality and loss of control eating illustrated in Figure 3 (Nolan and Geliebter 2016; Yeh and Brown 2014; Barnes et al. 2023; Vrabec et al. 2022). Based on these studies, the constructs were significantly positively associated (*r*_pooled_ = 0.37, 95% CI: 0.28–0.45, *p* < 0.001), indicating that higher loss of control eating is linked to poorer sleep quality within adults. The positive correlation is thus a reflection of differing scoring conventions for the included measures (i.e., greater scores reflect poorer sleep quality but greater sleep disturbance). Even though the power to detect heterogeneity with only four studies is low, *Q*‐ and *I* ^2^ indicated substantial heterogeneity amongst the included studies (*Q* = 15.38, *p* < 0.01, *I* ^2^ = 76.8%).

In contrast to these consistently positive cross‐sectional associations, Krawitz (2011) did not identify any significant association when investigating the longitudinal association between students' sleep quality at the beginning of the semester and binge eating symptoms at the end of the semester (as measured by the Bulimic Investigatory Test, Edinburgh (BITE)).

##### 4.3.1.2 Insomnia

**FIGURE 4:**
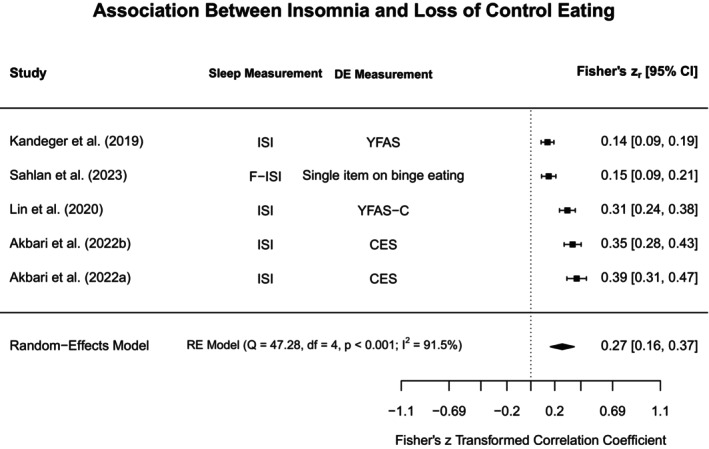
Meta‐analysis illustrating the association between insomnia and loss of control eating. CES=Compulsive Eating Scale, (F‐)ISI = (Farsi) Insomnia Severity Index, YFAS(‐C) = Yale Food Addiction Scale (for children).

Five analyses from four studies could be included in the meta‐analysis illustrated in Figure 4 (Sahlan et al. 2023; Akbari et al. 2022; Kandeger et al. 2019; Lin, Cheung, et al. 2020). Based on these studies, which all included mixed‐gender samples, insomnia symptoms were significantly associated with more loss of control eating (*r*_pooled_ = 0.26, 95% CI: 0.16–0.35, *p* < 0.001), though heterogeneity across studies was high (*Q* = 47.28, *p* < 0.001, *I* ^2^ = 91.5%).

Partly in line with these findings, Reichborn‐Kjennerud et al. (2004) identified a significant association between a one‐item assessment of insomnia symptoms (experienced during the last month) and a one‐item binge eating assessment only for women (OR = 1.90) of a population‐based sample, but not for men.

##### 4.3.1.3 Sleep Duration

**FIGURE 5:**
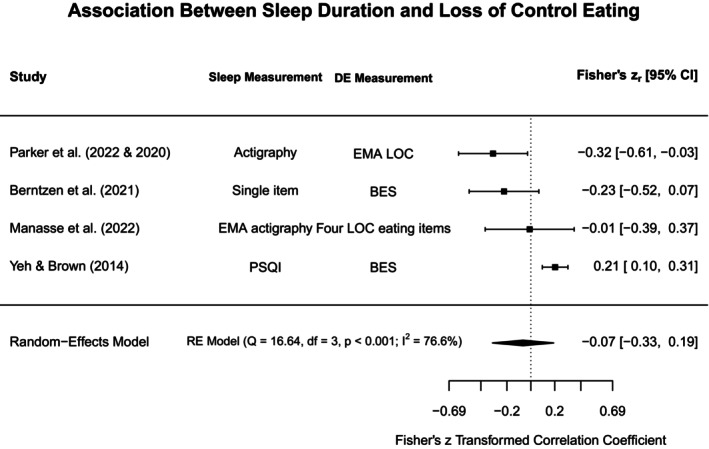
Meta‐analysis illustrating the association between sleep duration and loss of control eating. BES=Binge Eating Scale, EMA = Ecological Momentary Assessment, LOC = Loss of Control Eating, PSQI=Pittsburgh Sleep Quality Index.

The association between overall sleep duration (no distinction between week‐ and weekend days) and loss of control eating (Figure 5) could be meta‐analytically explored based on four studies (Yeh and Brown 2014; Manasse et al. 2022; Parker et al. 2020; Berntzen et al. 2021), of which two used an actigraphy‐based sleep assessment and an Ecological Momentary Assessment to measure loss of control eating (Manasse et al. 2022; Parker et al. 2020). There was no significant association (*r*_pooled_ = −0.06, 95% CI: −0.32 to 0.19, n.s.), and heterogeneity was high across studies (*Q* = 16.64, *p* < 0.001, *I* ^2^ = 76.6%), likely due to the utilisation of both self‐assessment and objective sleep measurements.

In contrast to these assessments of overall sleep duration, Vrabec et al. (2022), reported a significant small negative effect for the association of loss of control eating (Loss of Control Eating Scale, LOCES) and sleep quantity on weekdays (*β* = −0.12), not weekends, when controlling for age and sex in a US student sample. Furthermore, Borisenkov et al. (2020) only found significant group differences for weekday sleep duration and weekly averages when comparing students with food addiction (identified through the Yale Food Addiction Scale, YFAS) versus those without.

##### 4.3.1.4 Chronotype

**FIGURE 6:**
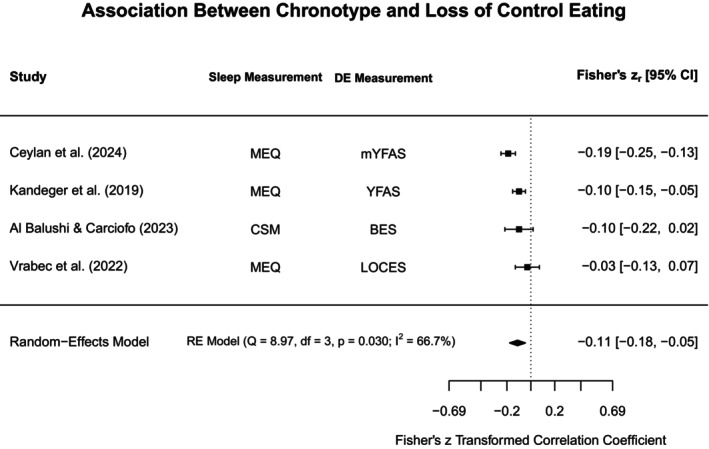
Meta‐analysis illustrating the association between chronotype and loss of control eating. BES=Binge Eating Scale, CSM = Composite Morningness Questionnaire, LOCES = Loss of Control Eating Scale, MEQ = Morningness–Eveningness Questionnaire, (m)YFAS = (modified) Yale Food Addiction Scale.

Four studies could be included to assess the association between chronotype and loss of control eating as part of a meta‐analysis illustrated in Figure 6 (Vrabec et al. 2022; Kandeger et al. 2019; al Balushi and Carciofo 2023; Ceylan et al. 2024). Overall, this analysis identified a significant association between evening chronotype and greater loss of control eating in mostly adult samples (*r*_pooled_ = −0.11, 95% CI: −0.18 to 0.05, *p* < 0.001). Heterogeneity across studies was significant and moderately high (*Q* = 8.97, *p* < 0.05, *I* ^2^ = 66.7%).

Studies that could not be included in the meta‐analysis, however, indicated non‐significant effects when investigating the association between the Munich Chronotype Questionnaire (MCQ) and Russian young adults' and adolescents' food addiction symptoms (YFAS) (Borisenkov et al. 2020) and the relationship between the Biological Rhythms Interview of Assessment in Neuropsychiatry (BRIAN) and food addiction symptoms in a small sample (*N* = 54) of Turkish women (Çeçen and Guleken 2023).

##### 4.3.1.5 Other Sleep Problems

Loss of control eating was associated with a variety of further individual sleep problems, such as ‘poor sleep’ and general nighttime sleep problems (Yeh and Brown 2014; Trace et al. 2012), perceptions of feeling insufficiently rested by sleep (Trace et al. 2012; Seigel et al. 2004), self‐reported use of sleep medication (Yeh and Brown 2014), and problems maintaining sleep (Seigel et al. 2004), with BMI potentially being a relevant factor to consider (Reichborn‐Kjennerud et al. 2004). Importantly, differences in study findings can potentially be attributed to the use of different measurement approaches. For example, sleep efficiency and sleep debt were only positively associated with binge eating when assessed via self‐report (Yeh and Brown 2014; Berntzen et al. 2021), not when measured via accelerometer (Berntzen et al. 2021; Barragán et al. 2021). Further, bed and wake times were only significantly associated with loss of control eating when assessed via Ecological Momentary Assessment and actigraphy (Parker et al. 2020), in contrast to self‐reported sleep timings (Seigel et al. 2004; Clark 2018; Kiltie et al. 2024). However, when binge eating was assessed in reference to *lifetime* behaviours, Trace and colleagues identified significant associations with self‐reports of waking up too early and sleep onset latency (Trace et al. 2012). Yet, present binge eating and food addiction symptoms did not show any cross‐sectional association with delayed sleep onset (Yeh and Brown 2014; Borisenkov et al. 2020; Seigel et al. 2004). Longitudinally, associations between loss of control eating and subsequent difficulties with falling asleep were found in two studies (Mason and Heron 2016; Nagata et al. 2021), even though this association did not hold after controlling for depression (Nagata et al. 2021). Finally, more research is needed to investigate associations with social jetlag, as loss of control eating (Vrabec et al. 2022), but not food addiction (Borisenkov et al. 2020; Ceylan et al. 2024), showed a significant association with this circadian misalignment.

#### 4.3.2 Bulimia and Compensatory Behaviours

##### 4.3.2.1 Sleep Quality

Only two studies assessed the association between sleep quality and bulimia symptoms, reporting no association between the Pittsburgh Sleep Quality Index (PSQI) and students' bulimic behaviours as measured by the Eating Attitudes Test (EAT‐16) (Suna and Ayaz 2022), and no significant prospective association between baseline PSQI reports and students' engagement in compensatory behaviours at the end of the semester (Krawitz 2011).

##### 4.3.2.2 Insomnia

Four studies explored the relationship between insomnia and bulimia symptoms; however, given heterogeneity in analysis approaches, it was not possible to conduct a meta‐analysis. Of those, only Cooper (2022) used a longitudinal research design and found insomnia (assessed by a single item) to be neither cross‐sectionally nor longitudinally significantly associated with subsequent compensatory behaviours (vomiting to lose weight and laxatives to lose weight). In contrast, cross‐sectional findings indicated significant associations between insomnia symptoms and adolescents' bulimic (*r* = 0.33) and compensatory behaviours (*r* = 0.21) (Liu et al. 2022), students' purging behaviours (*r* = 0.14) (Sahlan et al. 2023), as well as adolescents' engagement in extreme weight loss behaviours (vomiting or laxative use to avoid weight gain) (Ramos et al. 2023).

##### 4.3.2.3 Sleep Duration

Only Cooper (2022) assessed the relationship between self‐reported sleep duration and specific compensatory behaviours reporting a significant positive association between sleep duration and laxative use for weight loss (cross‐sectionally) at the first assessment point (*B* = 0.74, *p* < 0.05), and a negative effect when modelled as a predictor longitudinally (*B* = −0.84, *p* < 0.05). In addition, this study reported a negative effect for the association between sleep duration and vomiting for weight loss at the second assessment point (*B* = −0.84, *p* < 0.01) but not at any other timepoint.

##### 4.3.2.4 Chronotype

Only Schmidt and Randler (2010) reported on the relationship between chronotype and bulimic behaviours. The authors found a significant correlation between the Composite Scale of Morningness (CSM) and the bulimia subscale of the Eating Disorder Inventory‐2 (EDI‐2) (*r* = −0.13) in adolescent girls, and reported significantly higher bulimia scores for those being classified as evening type.

##### 4.3.2.5 Other Sleep Problems

Seven studies assessed bulimia symptoms or specific compensatory behaviours in the context of other sleep problems, reporting primarily significant positive associations. In regard to general bulimia symptoms, significant cross‐sectional associations were identified with general sleep problems (Hirai et al. 2022), difficulties initiating and maintaining sleep (Soares et al. 2011), but not bed and rise times (Schmidt and Randler 2010). When investigating specific compensatory behaviours, such as postprandial impulses to vomit, Seigel et al. (2004) established significant associations with self‐reported early awakening and feeling insufficiently rested by sleep, but not difficulties initiating sleep. Further, when accounting for multiple testing, Kiltie et al. (2024) reported no significant effects, for associations between purging (measured via the Eating Pathology Symptoms Inventory = EPSI) and sleep timings. Finally, longitudinal research did establish prospective relationships between “vomiting after meals” and subsequent difficulties with initiating (*r* = 0.19) and maintaining sleep (*r* = 0.24), (Bos et al. 2013), however, longitudinal effects did not hold after controlling for depressive symptoms (Nagata et al. 2021).

#### 4.3.3 Dieting and Restrictive Eating

##### 4.3.3.1 Sleep Quality

Only two studies assessed an association between sleep quality and restrictive eating behaviours. Suna and Ayaz (2022) reported no significant association between oral control behaviours, dieting (EAT‐26) and sleep quality in a student sample when comparing good and poor sleepers as identified by the PSQI, and Walker et al. (2018) reported no significant association between a single restrictive‐eating item and two items measuring sleep quality when accounting for students' trait anxiety.

##### 4.3.3.2 Insomnia

Three studies reported significant positive associations when assessing the relationship between insomnia and restrictive eating behaviours in primarily female adolescents and young adults. More specifically, Lombardo et al. (2010) reported a significant association between restrictive eating behaviours and insomnia severity within a student population (effect size not reported) and Lauer et al. (2021), who assessed the relationship between insomnia and weight loss intentions, identified increased insomnia symptoms in girls who reported these intentions (*η* ^2^_partial_ = 0.03). Similarly, Liu et al. (2022) reported a significant positive correlation between the Athens Insomnia Scale (AIS) and the dieting‐related items from the Eating Attitude Test (EAT‐19) (*r* = 0.36).

##### 4.3.3.3 Sleep Duration

Utilising self‐report assessments for sleep duration, Cooper (2022) did not find any cross‐sectional or longitudinal significant associations with restrictive eating items (*N* = 18,922 at baseline); however, Gallant et al. (2013) identified significantly more previous (OR = 1.3) and repeated weight loss behaviours (OR = 1.39) in a Canadian student sample (*N* = 3069) who were classified as short sleepers (< 7 h).

##### 4.3.3.4 Chronotype

Only De Young et al. (2022) assessed the relationship between chronotype (Morningness–Eveningness Questionnaire) and the EDE‐Q subscale of cognitive restraint in a student sample, finding no significant association between the two established measures.

##### 4.3.3.5 Other Sleep Problems

The majority of studies that included additional measures of sleep difficulties found significant small positive associations with dieting and restrictive eating. This included cross‐sectional associations with general sleep difficulties (Cooper 2022; Hirai et al. 2022; Soares et al. 2011; Bos et al. 2013; Bener et al. 2006), feeling insufficiently rested by sleep (Seigel et al. 2004), sleep‐related daytime dysfunction (Hirai et al. 2022), nighttime awakening (Seigel et al. 2004; Soares et al. 2011; Bos et al. 2013), and difficulties falling asleep, with females potentially being uniquely affected (Soares et al. 2011). Longitudinally, associations were either non‐significant (Cooper 2022) or did not hold after accounting for symptoms of depression, except for the prospective association with difficulties falling asleep (Nagata et al. 2021). Interestingly, Bos et al. (2013) reported a significant small positive association between students' avoidance to eat when hungry and their subsequent difficulties initiating sleep (*r* = 0.14), but no cross‐sectional association at the baseline assessment.

Further, a non‐significant association was identified with sleep onset latency for a single item measuring restrictive eating in US students (Walker et al. 2018), as well as women's recurrent weight‐reducing attempts (Seigel et al. 2004), and no associations were found when assessing the relationship between dieting and sleepiness/hypersomnia (Bener et al. 2006), self‐reported early morning awakening (Seigel et al. 2004), or between individuals' cognitive restraint and their self‐reported sleep timings (Kiltie et al. 2024).

#### 4.3.4 Excessive Exercise

##### 4.3.4.1 Sleep Quality

Only Ahorsu et al. (2023) assessed the relationship between the PSQI and the Exercise Addiction Inventory Youth Version (EAI‐Y), identifying a significant positive correlation in a large adolescent population (*r* = 0.12).

##### 4.3.4.2 Insomnia

**FIGURE 7:**
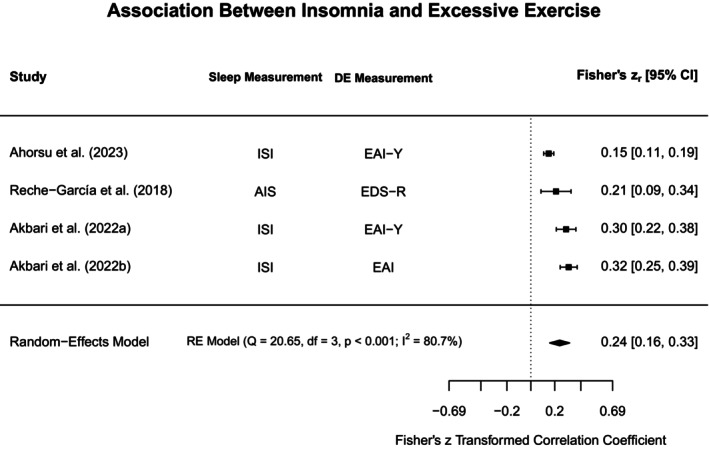
Meta‐analysis illustrating the association between insomnia and excessive exercise. AIS = Athens Insomnia Scale, EAI(‐Y) = Exercise Addiction Inventory (for young people), EDS‐*R* = Exercise Dependence Scale‐Revised, ISI = Insomnia Severity Index.

Four analyses from three studies could be included in the meta‐analysis illustrated in Figure 7 (Akbari et al. 2022; Ahorsu et al. 2023; Reche‐Garcia et al. 2018). Based on these studies, insomnia symptoms were significantly associated with higher excessive exercise values (*r*_pooled_ = 0.24, 95% CI: 0.16–0.32, *p* < 0.001), though heterogeneity across studies was high (*Q* = 20.65, *p* < 0.001, *I* ^2^ = 80.7%).

##### 4.3.4.3 Other Sleep Problems

Only Kiltie et al. (2024) explored other sleep problems in the context of excessive exercise, identifying no significant associations between students' excessive exercising and their bed –or wake times (using a criterion of *p* < 0.001 to determine significance to account for multiple testing).

#### 4.3.5 Body Image

##### 4.3.5.1 Sleep Quality

Three studies assessed a relationship between body image and the PSQI. Of these, Ahorsu et al. (2023) and Hao et al. (2023) identified significant positive cross‐sectional associations between sleep quality and body image concerns (*r* = 0.46, Body Image Concern Inventory), as well as body dissatisfaction as measured by a visual scale (*β* = 0.19). Only one longitudinal study assessed the relationship between sleep quality and weight and shape concerns based on the Eating Disorder Examination Questionnaire (EDE‐Q) (Krawitz 2011), demonstrating that sleep quality assessed at the first timepoint did not significantly predict US students' shape or weight concerns as measured approximately 2 months later.

##### 4.3.5.2 Insomnia

**FIGURE 8:**
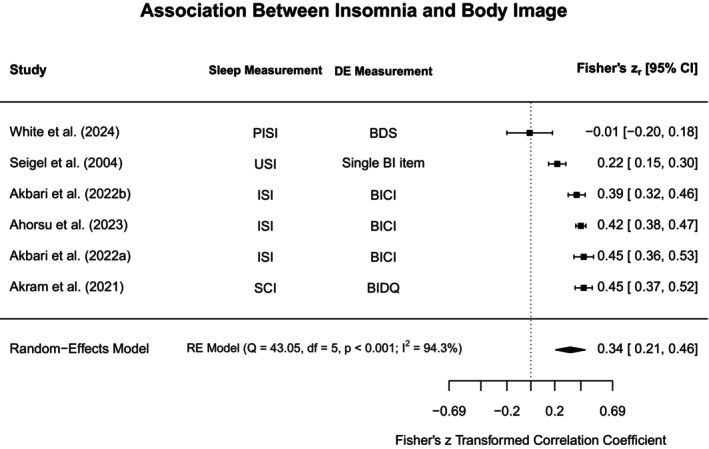
Meta‐analysis illustrating the association between insomnia and body image. BDS = Body Dissatisfaction Scale, BI = Body Image, BICI = Body Image Concern Inventory, BIDQ = Body Image Disturbance Questionnaire, PISI = Paediatric Insomnia Severity Index, SCI = Sleep Condition Indicator, USI = Uppsala Sleep Inventory.

Six analyses from five studies could be included in a meta‐analysis to assess the associations between body image and insomnia illustrated in Figure 8 (Akbari et al. 2022; Seigel et al. 2004; Ahorsu et al. 2023; White et al. 2024; Akram et al. 2021). Overall, there was a significant positive association between insomnia and body image related concerns (*r*_pooled_ = 0.32, 95% CI: 0.21–0.43, *p* < 0.001), with heterogeneity across studies being very high (*Q* = 43.05, *p* < 0.001, *I* ^2^ = 94.3%).

Birkeland et al. (2012), however, did not find a significant association between insomnia and a four‐item body image scale long‐term when assessing this relationship longitudinally across a 17‐year timespan (age 13–30).

##### 4.3.5.3 Sleep Duration

Five studies assessed the cross‐sectional association between self‐reported sleep duration and different facets of body image, highlighting the potential influential role of different body image components (e.g., dissatisfaction vs. appearance orientation) and participants' age for this association. White et al. (2024) reported a positive correlation between time‐in‐bed and the Body *Dis*satisfaction Scale (BDS) in adolescents (*N* = 106, *r* = 0.23), while Rosenbaum et al. (2023) found small positive associations between US female students' sleep duration and body appreciation (*r* = 0.22) as well as appearance evaluation (*r* = 0.20), but not appearance orientation (*r* = 0.03). In line with this, Lew et al. (2020) found American adolescents with short sleep duration to display higher odds of being categorised as high risk for poor body image. Park et al. (2020) identified significantly shorter sleep length in South Korean women who overestimated their body weight, but could not replicate this finding in women aged 65 and above. Finally, no significant differences in sleep timings of Japanese female high school students were identified by Mori et al. (2009) for different body image groups (based on a single‐item assessment).

##### 4.3.5.4 Chronotype

Four studies assessed the association between chronotype and body image concerns, indicating an association between evening type and body image concerns. Schmidt and Randler (2010), for example, found a small negative effect for the relationship between chronotype and drive for thinness (*r* = −0.12) as well body dissatisfaction (*r* = −0.24). Equally, Prieto et al. (2012) reported that adolescents who were classified as evening type exhibited a significantly poorer body image. Similarly, De Young et al. (2022) found significant small negative associations between chronotype and shape concerns (*r* = −0.19) as well as weight concerns (*r* = −0.18). Only Natale et al. (2008) reported a non‐significant effect for the association between body dissatisfaction and the Morningness–Eveningness Questionnaire (MEQr) in their non‐clinical control group.

##### 4.3.5.5 Other Sleep Problems

Significant effects were reported for the association of general sleep problems and body image concerns in all studies that explored this relationship. This included measurements of body dissatisfaction (Bruck and Astbury 2012; Matias et al. 2020; Wroblevski et al. 2022), a desire to be thinner (Bos et al. 2013), body weight misperception (Lo et al. 2011), and general body image assessments (Babayan et al. 2018). Furthermore, body image concerns were significantly associated with nighttime awakening (Seigel et al. 2004; Bos et al. 2013), nightmares (Lo et al. 2011), sleepiness (Altan et al. 2018), and feeling insufficiently rested by sleep (Seigel et al. 2004). While two studies reported significant associations for the relationship between body image and sleep onset latency both cross‐sectionally (Taut et al. 2018) and longitudinally (Bos et al. 2013), Seigel et al. (2004) reported a non‐significant effect for the association between women's self‐reported difficulties with initiating sleep and their body dissatisfaction, as well as weight gain fears (assessed via single items). Equally mixed findings were identified for self‐reported sleep timings, where no associations were found when sleep timings were assessed irrespective of week‐ and weekend timings (Seigel et al. 2004; Kiltie et al. 2024) with only Schmidt and Randler (2010) reporting significant associations between German adolescent girls' body dissatisfaction and their week‐ and weekend bedtimes, as well as their rising times on weekends. Finally, Fernández Argüelles et al. (2022) reported a significant association between high school students' body image discrepancy and their reduced sleep efficiency (assessed via actigraphy), highlighting the need for more objective sleep assessments in this area of research.

#### 4.3.6 Night Eating

##### 4.3.6.1 Sleep Quality

**FIGURE 9:**
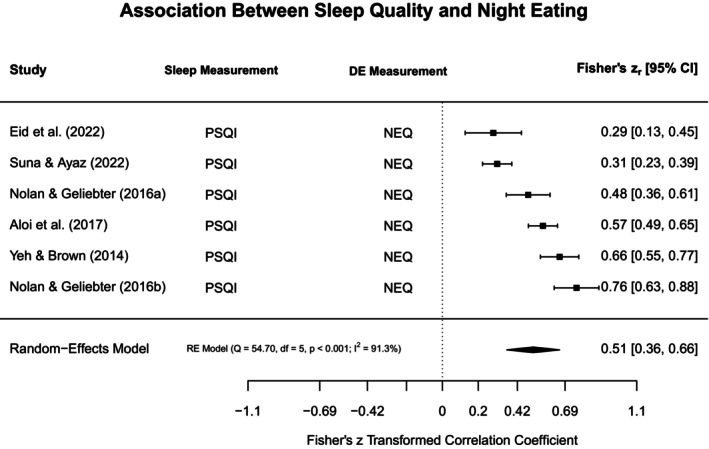
Meta‐analysis illustrating the association between sleep quality and night eating. NEQ = Night Eating Questionnaire; PSQI = Pittsburgh Sleep Quality Index.

Six reports of associations from five studies were included in the meta‐analysis assessing the association between night eating and sleep quality illustrated in Figure 9 (Nolan and Geliebter 2016; Yeh and Brown 2014; Suna and Ayaz 2022; Aloi et al. 2017; Eid et al. 2022). Overall, the analysis identified a significant positive effect between poorer sleep quality and night eating symptoms (*r*_pooled_ = 0.47, 95% CI: 0.35–0.58, *p* < 0.001), yet heterogeneity was very high across studies (*Q* = 54.70, *p* < 0.001, *I* ^2^ = 91.3%).

In line with the findings included in the meta‐analysis, Cakir et al. (2018), Lundgren et al. (2008), Farhangi (2019) and Aleksic et al. (2023) reported significantly poorer sleep quality in participants who exhibited symptoms of night eating. Blouchou et al. (2024) equally reported a higher likelihood of poor sleep in participants who were classified as experiencing night eating, but only if a less strict cut‐off was used to classify night eaters. In contrast, Ee and Gan (2022) did not find any significant associations between being classified as a night eater and experiencing poor sleep quality, which could be explained through the low reliability of the NEQ in the study's Malay student sample. In an Australian longitudinal study, Eid et al. (2022) identified a small significant effect for the association between the NEQ and PSQI cross‐sectionally (*β* = 0.28) but not longitudinally for sleep quality three months later (*β* = 0.06) when controlling for anxiety, depression, and baseline PSQI values. Finally, Nolan and Geliebter (2019) found consistently significant positive associations between the PSQI and some (e.g., depressed mood, morning anorexia), but not all NEQ items, when assessing the relationship between sleep quality and individual NEQ items in both a student and community population.

##### 4.3.6.2 Insomnia

Two cross‐sectional studies reported on associations between insomnia and night eating. Both Lee and Suh (2018) and Kandeger et al. (2018) found medium to large significant positive associations between the Insomnia Severity Index (ISI) and the NEQ (*r* = 0.54 and 0.38).

##### 4.3.6.3 Sleep Duration

Five cross‐sectional studies assessed an association between sleep duration and night eating. Three of these studies identified significant effects, finding a positive small (*r* = 0.10) (Suna and Ayaz 2022) to medium (*r* = 0.33) (Yeh and Brown 2014) association, and significantly shorter sleep duration in participants who displayed night eating symptoms (*n* = 19) versus those who did not (*n* = 22) (Lundgren et al. 2008). De Young et al. (2022), who explored only the Night Eating Questionnaire (NEQ) subscales “Morning Anorexia” and “Evening Hyperphagia” and assessed sleep via actigraphy, found a small significant association for the former, not for the latter subscale (longer sleep episodes were associated with greater morning anorexia). Finally, Farhangi (2019) did not identify significant group differences in sleep duration between a very small sample of adolescent boys who displayed night eating symptoms (*n* = 11), and boys who did not report night eating symptoms (*n* = 73).

##### 4.3.6.4 Chronotype

**FIGURE 10:**
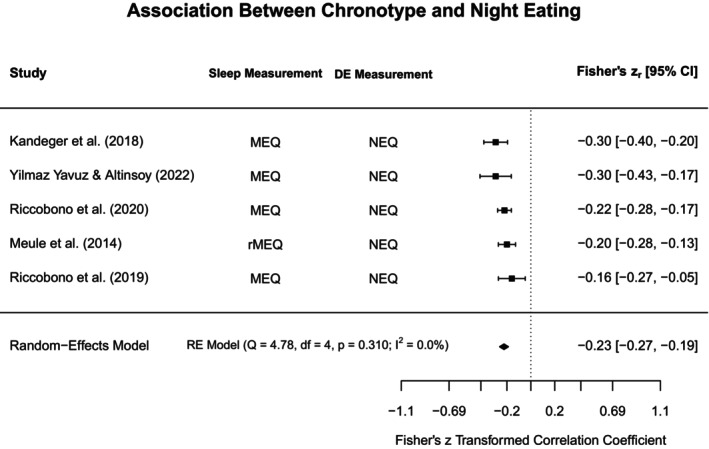
Meta‐analysis illustrating the association between chronotype and night eating. MEQ = Morningness–Eveningness Questionnaire, NEQ = Night Eating Questionnaire.

Five studies were included in a meta‐analysis investigating the relationship between night eating and chronotype illustrated in Figure 10 (Kandeger et al. 2018; Meule et al. 2014; Riccobono et al. 2019, 2020; Yilmaz Yavuz and Altinsoy 2022). Overall, this analysis identified a significant effect between evening chronotype and night eating symptoms (*r*_pooled_ = −0.22, 95% CI: −0.26 to −0.19, *p* < 0.001), with very low heterogeneity across studies (*Q* = 4.78, *p* = 0.31, *I* ^2^ = 0%).

Additionally, Lundgren et al. (2008) identified significantly lower levels on the Morningness–Eveningness Scale (more eveningness) in participants with night eating symptoms when compared to those who did not exhibit any symptoms. Equally, Özata Uyar et al. (2023) reported significantly higher night eating symptoms in those participants classified as evening type. In line with these findings, De Young et al. (2022) who only looked at Morning Anorexia and Evening Hyperphagia separately, reported a significant association between these night eating symptoms and evening chronotypes.

In contrast, only Blouchou et al. (2024) reported a positive association between morningness (measured by the Sleep, Circadian Rhythms, and Mood (SCRAM) questionnaire) and night eating when utilising a high cut‐off for night eating values in an adult population.

##### 4.3.6.5 Other Sleep Problems

Eight studies reported on the association between general sleep problems and night eating. All but one study found a significant *positive* association between these constructs (Yeh and Brown 2014; Suna and Ayaz 2022; Eid et al. 2022; Lundgren et al. 2008; Nolan and Geliebter 2019; Gundogdu and Erdogdu Yildirim 2023; Tholin et al. 2009), with only Farhangi (2019) reporting significantly more sleep disturbances in adolescent boys who were not exhibiting night eating symptoms (*n* = 73 compared to *n* = 11 with night eating).

Similarly, the majority of studies found significant positive small to moderate associations between night eating and sleep onset latency (Yeh and Brown 2014; Suna and Ayaz 2022; Lundgren et al. 2008; Tholin et al. 2009). Eid et al. (2022) found a small positive association between sleep onset latency and Australian adults' night eating symptoms cross‐sectionally, but no significant association between night eating and subsequent sleep onset latency three months later was found when adjusting for baseline sleep difficulties. Only Farhangi (2019) found no significant differences in sleep onset latency in a smaller sample of adolescent boys when comparing night eaters (*n* = 11) with those not displaying any symptoms (*n* = 73).

Despite having only limited research available, significant associations were also found for the relationship between night eating and experiencing more nightmares (Lee and Suh 2018), as well as feeling insufficiently rested by sleep (Lundgren et al. 2008; Tholin et al. 2009). Significant cross‐sectional associations were also reported for the association with daytime dysfunction (Yeh and Brown 2014; Suna and Ayaz 2022; Eid et al. 2022; Lundgren et al. 2008; Farhangi 2019), however, Eid et al. (2022) did not find a prospective association when adjusting for baseline levels. No significant association was reported for the relationship between night eating and sleepiness (ESS), when comparing participants with (*n* = 19) and without night eating symptoms (*n* = 22) (Lundgren et al. 2008).

Primarily small to moderate associations were found for the relationship between poorer sleep efficiency and night eating (Yeh and Brown 2014; Eid et al. 2022; Lundgren et al. 2008; Farhangi 2019), with only Suna and Ayaz (2022) reporting a small non‐significant effect for the association between night eating and *habitual* sleep efficiency.

Mixed findings were identified for the association between night eating and the use of sleep medication. For instance, Yeh and Brown (2014) found a significant positive association between the NEQ and the use of sleep medication in an Australian adult sample (*r* = 0.25), and Lundgren et al. (2008) reported a higher use of sleep medication in participants who were classified as night eaters. However, Suna and Ayaz (2022) did not find a significant association between students' sleep medication use and their self‐reported night eating symptoms, and Eid et al. (2022) did not find any association between adults' sleep medication use and their night eating behaviours. No significant group differences in sleep medication use were identified between participants with and without night eating symptoms (Farhangi 2019), which is not surprising considering the study's adolescent sample.

Finally, when assessing the relationship between students' bedtimes and night eating behaviours, Aleksic et al. (2023) identified significantly later bedtimes for students who were classified as night eaters.

#### 4.3.7 Overall ED Symptomatology

##### 4.3.7.1 Sleep Quality

**FIGURE 11:**
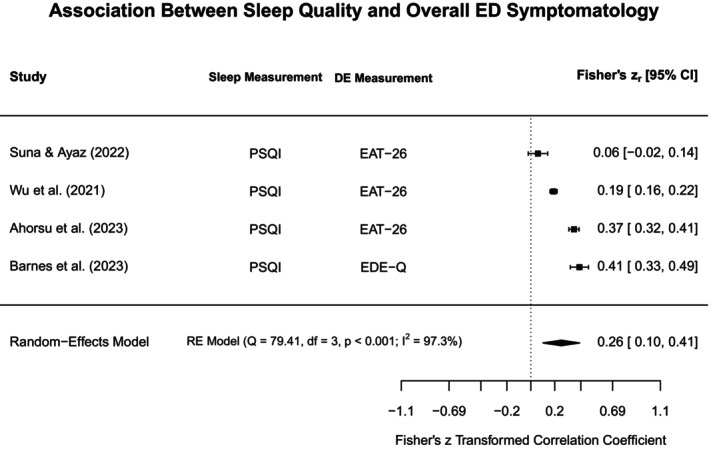
Meta‐analysis illustrating the association between sleep quality and overall eating disorder symptomatology. EAT = Eating Attitudes Test, EDE‐Q = Eating Disordered Examination Questionnaire, ED = Eating Disorder, PSQI = Pittsburgh Sleep Quality Index.

Four studies were eligible to be included in the meta‐analysis assessing the relationship between sleep quality and general ED symptomatology illustrated in Figure 11 (Barnes et al. 2023; Suna and Ayaz 2022; Ahorsu et al. 2023; Wu et al. 2021). Overall, the meta‐analysis identified a significant effect for the relationship between poor sleep and ED symptomatology (*r*_pooled_ = 0.25, 95% CI: 0.10–0.39, *p* < 0.01), high heterogeneity was identified across studies (*Q* = 79.41, *p* < 0.001, *I* ^2^ = 97.3%).

In line with this finding, Arslan and Aydemir (2019) found a significant association between sleep quality and disordered eating attitudes (*χ* ^2^ = 17.66) and Hasan et al. (2023) reported a significant association between poorer sleep quality and higher EAT‐26 values (no standardised effect size reported). Only Hirai et al. (2022) found no group differences in overall sleep quality between a very small group of participants identified as experiencing ED symptoms (*n* = 7) and participants who were at low risk for an ED (*n* = 190).

##### 4.3.7.2 Insomnia

**FIGURE 12:**
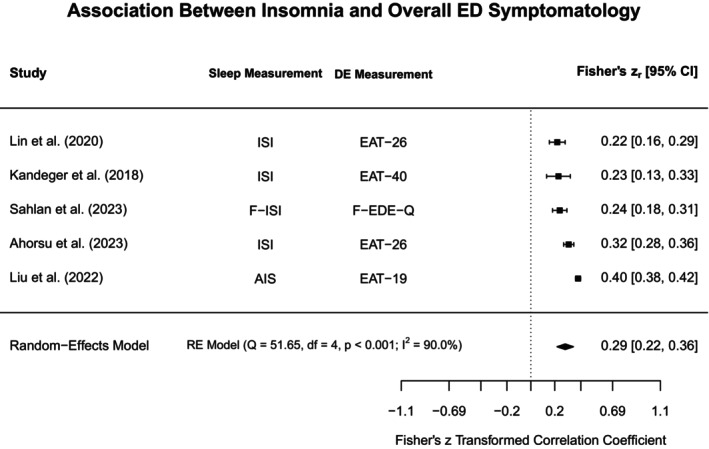
Meta‐analysis illustrating the association between insomnia and overall eating disorder symptomatology. AIS = Athens Insomnia Scale, EAT = Eating Attitudes Test, (F‐)EDE‐Q = (Farsi) Eating Disorder Examination Questionnaire, (F‐)ISI = (Farsi) Insomnia Severity Index.

Five studies were eligible for inclusion in the meta‐analysis investigating the association between insomnia and general ED symptomatology illustrated in Figure 12 (Lin, Jiang, et al. 2020; Sahlan et al. 2023; Liu et al. 2022; Ahorsu et al. 2023; Kandeger et al. 2018). Overall, the meta‐analysis identified a significant association between insomnia symptoms and disordered eating (*r*_pooled_ = 0.28, 95% CI: 0.22–0.35, *p* < 0.001), however high heterogeneity was identified across studies (*Q* = 51.65, *p* < 0.001, *I* ^2^ = 90%).

In line with the findings of the meta‐analysis, Lombardo et al. (2014) and Lombardo et al. (2013) identified significant positive associations between ED symptoms and insomnia severity, using the Disordered Eating Questionnaire (DEQ) (*χ* ^2^ = 49.02), while Aspen et al. (2014) reported higher insomnia symptoms in women who were classified as higher risk for an ED based on the Eating Disorder Examination Questionnaire (EDE‐Q) (no standardised effect size reported). Only Hirai et al. (2022) reported a very small non‐significant association with the Athens Insomnia Scale (AIS) when comparing Japanese high school students displaying more ED symptoms (EAT‐26 ≥ 20) with those at low risk.

##### 4.3.7.3 Sleep Duration

Five studies utilising different analytic techniques reported on the association between sleep duration (consistently measured through self‐report) and general ED symptomatology, revealing mixed findings. Using the EAT‐26 and a cross‐sectional research design, Suna and Ayaz (2022) did not establish a significant association between these constructs (*r* = −0.001), and Hirai et al. (2022) did not identify any significant group differences for different sleep length categories when comparing those classified as experiencing ED symptoms with participants who did not display symptoms. Cooper (2022), however, reported different findings for the relationship between sleep duration and a composite of restrictive and compensatory behaviours when utilising different methodological approaches. When assessing sleep duration as participants' average nightly hours of sleep, no significant association was found. Yet, at their third assessment point, when participants reported on their bed‐ and wake times during the week and the weekend, Cooper found a significant negative association between sleep duration (measured as average sleep length weighted across weekdays and weekends) and the utilised composite measure. Richardson et al. (2025), who assessed correlations between sleep duration and the Children's Attitude Test (ChEAT) both cross‐sectionally and longitudinally, found significant negative associations (*r* = −0.10 to −0.14) for all cross‐sectional analyses (except the last timepoint) and mixed findings for longitudinal associations. Finally, Johnson (2020), the only study utilising an experimental research design, found no significant group differences in disordered eating between their sleep‐deprived experimental group and control participants.

##### 4.3.7.4 Chronotype

**FIGURE 13:**
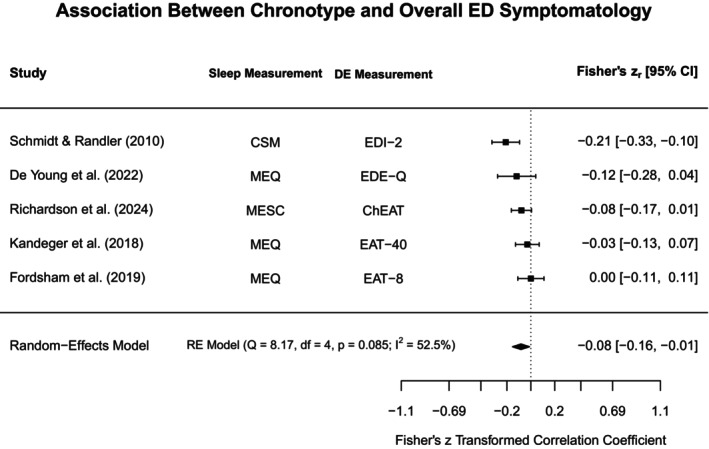
Meta‐analysis illustrating the association between chronotype and overall eating disorder symptomatology. CSM = Composite Morningness Questionnaire, (ch)EAT = (Children's) Eating Attitudes Test, EDI‐2 = Eating Disorder Inventory, EDE‐Q = Eating Disorder Examination Questionnaire, MEQ = Morningness–Eveningness Questionnaire, MESC = Children's Morningness–Eveningness Scale.

Five studies were eligible for inclusion in the meta‐analysis investigating the association between chronotype and general ED symptomatology illustrated in Figure 13 (Schmidt and Randler 2010; De Young et al. 2022; Kandeger et al. 2018; Richardson et al. 2025; Fordsham et al. 2019). Overall, the meta‐analysis identified a significant small association between evening type and disordered eating (*r*_pooled_ = −0.08, 95% CI: −0.16 to −0.01, *p* < 0.05). Heterogeneity across studies was non‐significant but moderate (*Q* = 8.17, *p* = 0.08, *I* ^2^ = 52.5%).

In contrast to these findings, Hasan et al. (2023) found no significant association between the EAT‐26 and the MEQ in a student sample, and Natale et al. (2008) found no significant association between the EDI‐2 and a reduced version of the Morningness–Eveningness Questionnaire in their non‐clinical control population. Additionally to the cross‐sectional baseline findings included in the meta‐analysis, Richardson et al. (2025) reported mixed findings for further cross‐sectional and longitudinal associations, with higher correlation coefficients being identified for associations between later timepoints, when adolescent participants were older.

##### 4.3.7.5 Other Sleep Problems

Ten studies assessed an association between general sleep problems and general ED symptomatology, with all of them reporting significant positive associations (Bahri et al. 2015; Suna and Ayaz 2022; Hirai et al. 2022; Soares et al. 2011; Bos et al. 2013; Lee et al. 2021; Chardon et al. 2016; Figueroa et al. 2024; Hafstad et al. 2013), except for Richardson et al. (2025) who reported mixed findings for different time points. Longitudinally, Hafstad et al. (2013) found a significant small positive association with children's parent‐reported early sleep problems (experienced before age five) and later disordered eating attitudes (EAT) (*r* = 0.11) and Bos et al. (2013) identified a significant longitudinal association between disordered eating attitudes at baseline and subsequent sleep disturbances at the first and second follow‐up (each timepoint one academic year apart, no standardised effect sizes reported).

Similarly, significant positive associations were reported for sleep onset latency and awakening during the night, both cross‐sectionally (Soares et al. 2011) and longitudinally (Bos et al. 2013). While non‐significant findings were reported for the association with sleep medication use (Suna and Ayaz 2022), mixed findings were reported for associations with sleep timings (Schmidt and Randler 2010; Hirai et al. 2022), likely due to differences between weekday and weekend behaviours. Equally mixed findings were identified for the relationship with daytime dysfunction, with Hirai et al. (2022) reporting a significant correlation effect for the association between the AIS daytime functioning item and the EAT (*r* = 0.29), while Suna and Ayaz (2022) reported a non‐significant association between the EAT and the daytime dysfunction item of the PSQI.

Finally, Chardon et al. (2016) reported a significant positive effect for the association between children's parent‐reported disordered eating attitudes and behaviours (ChEAT) and daytime sleepiness (*r* = 0.37). Richardson et al. (2025) further identified consistently significant cross‐sectional and longitudinal associations between sleepiness and disordered eating across all five assessment points. Only Hirai et al. (2022) found a non‐significant effect (adjusted for multiple testing) when comparing different levels of daytime sleepiness between those participants displaying ED symptoms (*n* = 17) and those without exhibiting symptoms.

### 4.4 Included Moderators and Mediators—Narrative Review

Table S3 provides a narrative overview of all moderator‐ and mediator‐analyses which were previously tested for the relationship between sleep and disordered eating in included studies. While the majority of studies controlled for relevant covariates (e.g., gender, BMI, and age) (see Table S2), surprisingly few moderation analyses were conducted in the included studies, testing the moderating effect of weight status (Gallant et al. 2013) and gender (Hafstad et al. 2013) in the context of restrictive eating (Gallant et al. 2013), and general disordered eating (Hafstad et al. 2013). Of these, only weight status was identified as a significant moderator (i.e., the adjusted odds of reporting restrictive eating tendencies, perceived stress, and short sleep were only significantly elevated in under‐ and normal weight participants who engaged in repeated weight loss behaviours).

The most commonly tested mediator for the relationship between sleep and disordered eating was negative mood (Rosenbaum et al. 2023; Lee and Suh 2018; Gundogdu and Erdogdu Yildirim 2023; Wu et al. 2021; Lombardo et al. 2014), revealing mixed findings for different sleep‐ and disordered eating indicators (see Table S3). Anxiety was identified as a significant mediator by three studies (Lee and Suh 2018; Wu et al. 2021; Nguyen‐Rodriguez et al. 2010), while Rosenbaum et al. (2023) and Gundogdu and Erdogdu Yildirim (2023) reported non‐significant mediation effects for anxiety on the relationship between sleep duration and body image assessments (Rosenbaum et al. 2023), as well as insomnia and night eating (Gundogdu and Erdogdu Yildirim 2023).

Further mediators that showed significant effects were stress for the relationship between insomnia and night eating and the association between sleep duration and body appreciation (Rosenbaum et al. 2023; Gundogdu and Erdogdu Yildirim 2023), skipping breakfast in the context of chronotype and binge eating (al Balushi and Carciofo 2023), sleep‐associated monitoring and body‐image related distortions/coping processes in the context of body image disturbances and insomnia symptoms (Akram et al. 2021), impulsivity for the relationship between chronotype and food addiction (Kandeger et al. 2019), and problematic smartphone use in the context of sleep quality and general disordered eating (Wu et al. 2021). Non‐significant or mixed findings were identified for mediators such as general repetitive thinking (which was only identified as a significant mediator when poor sleep predicted ED symptomatology, not the other way around) (Richardson et al. 2025), physical pain and social jetlag (n.s.) (Ceylan et al. 2024), the effect of the timing of light exposure (only significant for the effect of sleep/wake preferences on evening hyperphagia, not morning anorexia) (De Young et al. 2022), and sleep efficiency (only relevant for morning anorexia, not hyperphagia) (De Young and Bottera 2022).

## 5 Discussion

Based on information provided by 89 studies, this systematic review demonstrated significant associations between sleep and various domains of disordered eating in non‐clinical populations. Specifically, small to moderate associations were identified for general ED symptomatology and loss of control eating across sleep quality and insomnia symptom assessments. While previous research has established significant associations between inadequate sleep and emotional eating (Zerón‐Rugerio et al. 2023), the current review further suggests a co‐occurrence of sleep problems and general disordered eating behaviours that requires more targeted exploration. Compared to other disordered eating behaviours, emotional eating and eating disinhibition have received heightened research attention in the context of sleep (Zerón‐Rugerio et al. 2023), likely due to their presumed relationship with overweight and obesity. Evidence included in the present review, however, supports further explorations of sleep's potential role in broader disordered eating symptom presentations.

Not surprisingly, especially considering the conceptual and diagnostic overlap between night eating syndrome and insomnia symptoms (Milano et al. 2012; Allison et al. 2010), night eating was moderately associated with all sleep problems and chronotype, where evening types displayed more night eating behaviours. Even though the directionality of the relationship between night eating and sleep problems is difficult to establish, addressing sleep problems in patients with night eating is seen as an essential part of clinical treatment approaches (Kucukgoncu et al. 2015). The association between night eating and evening chronotype may be related to altered hormone levels (Kandeger et al. 2018), but more research on the directionality and specific mechanisms linking night eating to sleep traits is needed. Future assessments looking into the relationship between night eating and sleep should consider disentangling the association of night *eating* behaviours from the sleep component of the symptom presentation.

The role of eating‐related physiological impacts on sleep has also been discussed as a consequence of anorexia nervosa, where malnutrition is hypothesised to affect neurological and endocrine functioning, and consequently sleep–wake patterns (Allison et al. 2016). In the present review, which included only non‐clinical populations and a wide variety of indicators to measure restrictive eating (individual items on weight control behaviours as well as validated questionnaire subscales), findings were mixed. While overall sleep quality did not appear to be related to restrictive eating in the two student populations where this association was tested, associations with specific sleep problems and insomnia partly exhibited small significant effects, potentially due to the focus on primarily female adolescents and young adults. Interestingly, restrictive eating was the only disordered eating behaviour that showed a small (non‐significant) association with morning chronotype, while all other disordered eating behaviours in this review, as well as mental health concerns generally, have been shown to be associated with eveningness (Taylor and Hasler 2018). Future research will need to investigate this relationship and involved mechanisms to identify if and why this pattern of association differs from other disordered eating behaviours.

Similarly, preliminary findings for body image concerns call for further exploration, considering that studies included in this review showed an overall tendency towards a positive association with sleep problems. Some of the inconsistencies identified in this review could potentially be addressed by aligning methodological approaches (e.g., visual assessments, validated questionnaires of comparable body image components), as many studies in this review utilised a variety of (often non‐validated single‐item) assessments to measure body dissatisfaction. Furthermore, the role of gender and age differences deserves further attention within this field.

Similar to research in ED populations (Cooper et al. 2020), studies assessing the relationship between sleep and bulimia symptoms, as well as specific compensatory behaviours, in the general population are scarce. Based on available research and previous findings within clinical populations (Abdou et al. 2018), there is a clear need for more studies investigating this relationship. As a compensatory behaviour, excessive exercise constitutes a particularly interesting concept, considering that healthy levels of exercising are associated with clear improvements in sleep quality (Xie et al. 2021), while vigorous exercise right before bedtime can negatively impact sleep (Stutz et al. 2019). As only few studies included in this review assessed excessive exercise in the context of sleep, more research into its impact on different sleep indicators is needed.

Interestingly, sleep duration showed no clear association with disordered eating on its own, highlighting the need to differentiate between general sleep traits and subjective perceptions of sleep quality and sufficiency. Importantly, future studies will need to investigate the role of subjective versus objective sleep duration measures, as current findings are primarily based on subjective self‐reports. Equally, the differentiation between week/workday and weekend/free day sleep patterns deserves further attention, potentially with a focus on sleep length and timing variability (Fang et al. 2021; Sletten et al. 2023). This includes the concept of social jetlag, which is increasingly receiving more attention in mental health research (Henderson et al. 2019; Caliandro et al. 2021), but has scarcely been studied in the context of disordered eating.

In order to progress research in the field of sleep and disordered eating, future studies will need to address the lack of longitudinal study designs to explore temporal and causal associations between both constructs, as it is currently still unclear *how* sleep and specific disordered eating behaviours are related to each other. Only 10 (~11%) out of the included 89 studies used some form of a longitudinal research design, with four of these drawing on non‐validated measurement tools to assess sleep and/or disordered eating, most likely due to restrictions of cohort study designs (i.e., a multitude of constructs being assessed at the same time). Focusing on longitudinal research will also aid in addressing open questions regarding symptom‐specific mediators for the relationship between sleep and disordered eating, as current evidence does not allow for the identification of clear patterns of association.

## 6 Strength and Limitations

The present review included a large number of studies, allowing for an extensive overview of the associations between sleep and disordered eating in non‐clinical populations. To the best of the authors' knowledge, this is the first review exploring the relationship between various sleep indicators and subtype‐specific disordered eating outcomes in non‐clinical populations, extending findings from previous reviews focusing on exclusively clinical populations. Thus, insights from the present review can inform prevention approaches but do not speak to symptoms of sleep disturbance within clinical presentations. The following limitations of this review need to be acknowledged: first, due to broad inclusion criteria, study populations and designs differed greatly across included research. Even though this heterogeneity was acknowledged when discussing findings, this review did not allow for detailed comparisons of populations and assessment methods, which would have helped to understand associations in a more nuanced way. Effects of heterogeneity within conceptual categories (e.g., “loss of control eating”, “restrictive eating”) due to our approach towards synthesising study findings will need to be explored in the future, once more research is available. Second, only a small number of studies could be included in individual meta‐analyses models, limiting the information value of these analyses, while highlighting the need for more research into subtype‐specific associations. Given this low number of studies included in each meta‐analysis model, no bias calculation could be conducted statistically and further explanatory models for associations could not be explored (including how study quality moderated effect sizes). Any reported pooled effects thus need to be considered preliminary and should be replicated differentiating different methodological approaches (e.g., pooled Ecological Momentary Assessment data, self‐assessments) to explore differences in associations once more evidence is available. Individual quality criteria were considered in the narrative review and eligibility criteria for meta‐analyses ensured that only studies with adequate quality were included in the analyses. Finally, terminologies used to describe sleep problems varied across studies, with terms like “disturbances” and “difficulties” often being open to interpretation. Furthermore, when it comes to the assessment of sleep, the terms ‘sleep quality’ and ‘insomnia’ are often conflated, likely due to the item‐overlap in prevalent scales (Niu et al. 2023), which complicates the evaluation of associations with mental health concerns. Even though the current review aimed to differentiate between distinct sleep constructs, it is important to acknowledge that individual studies sometimes classify concepts interchangeably.

## 7 Conclusion

The present systematic review and meta‐analysis identified clear associations between sleep‐related concerns and various disordered eating indicators in non‐clinical populations. Evaluating a broad range of research findings highlighted the need for more nuanced and rigorous research in this field. While general disordered eating behaviours, loss of control eating, and night eating exhibit generally consistent associations with poor sleep quality and sleep problems, associations with restrictive eating behaviours, bulimia symptoms, and body image concerns require further investigation.

## Author Contributions

**Marie‐Christine Opitz:** conceptualization, investigation, methodology, data curation, formal analysis, writing – original draft, writing – review and editing, project administration. **Nora Trompeter:** methodology, investigation, writing – review and editing. **Francisco Diego Rabelo‐da‐Ponte:** methodology, investigation, writing – review and editing. **Michelle Carroll:** investigation, writing – review and editing. **Kyle Buchan:** investigation, writing – review and editing. **Giulia Gaggioni:** conceptualization, methodology, writing – review and editing. **Sarah Moody:** investigation, writing – review and editing. **Sylvane Desrivières:** methodology, writing – review and editing, funding acquisition. **Nadia Micali:** funding acquisition, methodology, writing – review and editing. **Ulrike Schmidt:** conceptualization, writing – review and editing, funding acquisition. **Helen Sharpe:** conceptualization, methodology, supervision, funding acquisition, writing – review and editing.

## Ethics Statement

The authors have nothing to report.

## Conflicts of Interest

Nora Trompeter receives an honorarium from Wiley as Associate Editor for Mental Health Science. Nadia Micali receives an honorarium as associate editor on European Eating Disorders review. Francisco Diego Rabelo‐da‐Ponte receives royalties from Springer Nature.

## Supporting information


**TABLE S1:** Overview of relevant study findings.

**TABLE S2:** Overview of quality assessments.

**TABLE S3:** Narrative overview of mediators and moderators identified for the association of sleep and disordered eating.

## Data Availability

The authors have nothing to report.

## Appendix A Members of the EDIFY Consortium

Prof Ulrike Schmidt, Dr. Helen Sharpe, Dr. Karina Allen, Prof Heike Bartel, Prof Iain Campbell, Prof Sylvane Desrivières, Prof Richard Dobson, Dr. Amos Folarin, Dr. Tara French, John Kelly, Umairah Malik, Prof Nadia Micali, Sneha Raman, Dr. Zulqarnain Rashid, Prof Janet Treasure, Callum Bryson, Daire Douglas, Amelia Hemmings, Dr. Başak İnce Çağlar, Carina Kuehne, Sarah Moody, Dr. Marie‐Christine Opitz, Dr. Tamsin Parnell, Fiona Stephens, Dr. Nora Trompeter, Jessica Wilkins, Alice Bromell, Grace Davis, Cameron Eadie, Beck Heslop, Isabella Malvisi, Katie Mckenzie, Emma Scott, Chris Sims, Tallulah Street, Andreia Tavares‐Semedo, Eleanor Wilkinson, Lucz Zocek.
