# Supplementary material for: The Association Between Disordered Eating and Sleep in Non‐Clinical Populations—A Systematic Review and Meta‐Analysis
Source: J Sleep Res. 2025 Jun 30;35(2):e70117. doi: 10.1111/jsr.70117 (PMC13003291; doi:10.1111/jsr.70117)
Supplement: Supplementary file 1 — TABLE S1: Overview of relevant study findings. [file JSR-35-e70117-s002.docx]

Supplementary Table 1. Overview of Relevant Study Findings

| **Author(s)** | **Sleep Outcome** | **Disordered Eating Outcome** | **Findings reported** | **Effect sizes reported** |
| --- | --- | --- | --- | --- |
| Ahorsu et al. (2023) | Insomnia Severity Index (ISI): 7 items, Pittsburgh Sleep Quality Index (PSQI): 19 items | Exercise Addiction Inventory - Youth Version (EAI-Y): 6 items, Body Image Concern Inventory (BICI): 19 items, Eating Attitudes Tests (EAT-26): 26 items | There were significantly positive relationships between psychological distress, exercise addiction, insomnia, sleep quality, body image concerns, and eating disorders which ranged from small to large effect sizes. There was a significant direct effect of exercise addiction on insomnia (unstandardised coefficient =0.19, t=6.74, p<0.001), and sleep quality (unstandardised coefficient =0.13, t=5.36, p<0.001) | Correlations:  Exercise addiction + insomnia: r=0.15, p<0.01  Exercise addiction + sleep quality: r=0.12, p<0.01  Insomnia + body image concerns: r=0.40, p<0.01  Sleep quality + body image concerns: r=0.46, p<0.01 Insomnia + EAT: r=0.31, p<0.01 Sleep quality + EAT: r=0.35, p<0.01  Relevant parameters from mediation:  Direct effect of exercise addiction on insomnia: B(unstand.)=0.19, SE=0.03, t=6.7, p<0.001  Direct effect of exercise addiction on sleep quality: B=0.13, SE=0.02, t=5.36, p<0.001 |
| Akbari et al. (2022) | Insomnia Severity Index (ISI), Persian version: 7 items | Exercise Addiction Inventory (EAI), Persian version: 6 items, Exercise Addiction Inventory Youth Version (EAI-Y), Persian version: 6 items, Compulsive Eating Scale (CES), Persian version: 8 items, Body Image Concern Inventory (BICI), Persian version: 19 items | Positive association between insomnia and exercise addiction + body image concern + compulsive eating (both adults and adolescents)  Exercise addiction was a significant mediator in the relationship between problematic social media use and all mental health outcomes (both adults and adolescents).  All SEMs showed invariance between male and female participants. | Adolescents:  Insomnia + Exercise addiction: r=0.29  Insomnia + Body image concern: r=0.42  Insomnia + Compulsive eating: r=0.37  Adults:  Insomnia + Exercise addiction: r=0.31  Insomnia + Body image concern: r=0.37  Insomnia + Compulsive eating: r=0.34 |
| Akram et al. (2021) | Sleep Condition Indicator (SCI): 8 items, Sleep-Associated Monitoring Index (SAMI): 10 items | Assessment of Body Image Cognitive Distortions Form A (ABCD): 18 items, Body Image Disturbance Questionnaire (BIDQ): 7 items, Coping with Body Image Challenges Inventory (BICSI): 29 items | Insomnia symptoms were significantly related to increased levels of sleep-associated monitoring on awakening, sleep-associated monitoring through the day, body image cognitive distortion, body image disturbance, appearance fixing, avoidance and reduced acceptance. The association between insomnia symptoms and body image disturbance was mediated by body image cognitive distortions (z=−7.70, p<0.001), coping avoidance (z=−4.95, p < 0.001), rationale acceptance coping (z = − 2.93, p=0.003), appearance fixing coping (z=−3.76, p<0.001) and awakening sleep monitoring (z=−2.69, p=0.007). | Correlations (mistake in table 1):  Insomnia symptoms + Body image cognitive distortion: r=-0.38, p<0.001  Insomnia symptoms + Body image disturbance: r=-0.42, p<0.001  Insomnia symptoms + Coping (appearance fixing): r=-0.21, p<0.001  Insomnia symptoms + Coping (avoidance): r=-0.41, p<0.001 Insomnia symptoms + Coping (rationale acceptance): r=0.19, p<0.001  Regression (predicting body image disturbance, controlling for age and sex):  Overall model statistically significant (F=117.95, p<0.001), adjusted R2=59.3%  Insomnia symptoms predicting body image disturbance (covariates: age, sex): beta=-0.05, B=-0.42, p<0.001 [-0.06; -0.05] |
| al Balushi & Carciofo (2023) | Composite Scale of Morningness (CSM): 13 items | Binge Eating Scale (BES): 16 items | Morningness-eveningness and morning affect were negatively correlated with binge-eating (more eveningness was associated with more binge-eating), although the correlation with morningness-eveningness did not reach statistical significance | Correlations:  Morningness-Eveningness+ Binge eating: r=-0.10 (n.s.)  Morning affect + Binge eating: r=-0.23, p<0.01  Direct effect in mediation: Morningness-eveningness predicting binge-eating: b=-0.05 (n.s.) |
| Aleksic et al. (2023) | Socio-epidemiologic questionnaire: time at which participants went to sleep, sleep quality (quite poor, poor, average, good, quite good) | Night Eating Questionnaire: 14 items | Students with NES went to bed 1h later (on average 1am) compared to students without NES (on average midnight). A considerably higher proportion of students who had NES reported poorer sleep quality compared to those free from NES. | Group comparison:  Bedtime (M, SD) hours: No NES: M=0.04, SD=1.4 NES: M=1.6, SD=1.5 p=0.001  Sleep Quality:  Quite poor: No NES: n=15 (2.9%) NES: n=3 (18.8%)  Poor: No NES: n=45 (8.7%) NES: n=6 (37.5%)  Average: No NES: n=232 (44.8%) NES: n=3 (18.8%)  Good: No NES: n=183 (35.3%) NES: n=3 (18.8)  Quite good: No NES: n=39 (7.5%) NES: n=1 (6.3%)  p=0.001 |
| Aloi et al. (2017) | Pittsburgh Sleep Quality Index (PSQI) | Night Eating Questionnaire (NEQ): 14 items   Binge Eating Scale (BES): 16 items   Eating Disorder Examination Questionnaire (EDE-Q): 22 items | The NEQ showed positive correlations with the PSQI (r = .514, p < .001) | Correlation:  NEQ + PSQI: r = .514, p < .001 |
| Altan et al. (2018) | Pediatric Daytime Sleepiness Scale: 8 items | One question on body image: "What do you think about your body image?" | Low-level negative correlation between daytime sleepiness and being satisfied with one's body image. | Corrrelation between thinking about body image & PDSS score: p=-0.193, p<0.01  Prediction level of body image satisfaction on daytime sleepiness: beta =-0.146 p=0.006, t=-2.79 |
| Arslan & Aydemir (2019) | Pittsburgh Sleep Quality Index (PSQI) | Eating Attitudes Test (EAT): 40 items | Those with disrupted sleep quality were found to have disrupted eating attitudes | Pearson Chi-squared: χ^2^ = 17.66 (p=0.001)  Good sleep + normal eating: n=84 Good sleep + disrupted eating (EAT): n=64  bad sleep + normal eating: n=179 bad sleep + disrupted eating (EAT): n=208 |
| Aspen et al. (2014) | Insomnia Severity Index (ISI): 5-items | Eating Disorder Examination-Questionnaire (EDE-Q): 39-items | ISI scores for the high-risk women were greater than control women.   Rates of insomnia in the clinical range (as measured by the ISI cut-off) were significantly greater high-risk groups as compared to the control group, with prevalence rates increasing significantly by category.  Post hoc analyses indicated that women with clinical insomnia had significantly higher nocturnal eating frequency ratings as compared to those without clinical insomnia. | ISI: control M=6.8 (0.6) vs high risk M=8.1 (0.3) Clinical ISI: control M=5.2 (0.02) vs high risk M=13.9 (0.02) |
| Babayan et al. (2018) | Sleeping problems (no validated metric used), dichotomous variable - yes/no sleeping problem | Body Self-Image Questionnaire: 23 items | Regarding body image, significant differences were noticed according to sleeping problem (F=17.81, p=<.001). | Presence of sleeping problems: Body image: M=73.74 (SD=11.63)  Absence of sleeping problems: Body image: M=78.83 (SD=13.78)  F=12.94, p=<.001 |
| Bahri et al. (2015) | Insomnia questions from the General Health Questionnaire (GHQ-28): one subscale on "distress and insomnia" (7 items) | Eating Attitudes Test (EAT-26): 26 items  Eating Disorder Inventory (EDI-64): 64 items | Positive significant correlation between components of mothers' general health (incl. sleep disorder) and mothers' eating attitudes. | Correlations within mothers not to be identified from the manuscript (values reported relate to the relationship of mother's health and their children's eating behaviours) |
| Barnes et al. (2023) | Pittsburgh Sleep Quality Index (PSQI): 19 items | Eating Loss of Control Scale (ELOCS): 18 items  Eating Disorder Examination Questionnaire - Version 17 (EDE-Q): reference to past 28 days | PSQI global scores were significantly positively correlated with eating-disorder psychopathology. When considered as a group, participants with poor sleep quality endorsed higher levels of eating-disorder psychopathology | Correlations:  EDE-Q global + PSQI global: r=0.39, p<0.01  Objective binge eating episode frequency + PSQI global: r=0.26, p<0.01  Subjective binge eating episode frequency + PSQI global: r=0.19, p<0.01  ELOCS + PSQI global: r=0.38, p<0.01  Group differences:  Good sleep quality (n=305): EDE-Q: M=1.46, SD=1.21  OBE: M=1.02, SD=2.87  SBE: M=1.30, SD=3.85  ELOCS: M=1.55, SD=3.00  Poor sleep quality (n=343):  EDE-Q: M=2.37, SD=1.35  OBE: M=2.63, SD=4.91  SBE: M=3.00, SD=5.42  ELOCS: M=1.81, SD=2.24  EDE-Q: t=-8.99 d=0.71 F (age, BMI, sex)=48.42, Eta2=0.07  OBE: t=-5.16 d=0.40 F (age, BMI, sex)=16.20 Eta2=0.03  SBE: t=-4.62 d=0.36 F (age, BMI, sex)=13.41 Eta2=0.02  ELOCS: t=-9.12 d=0.71 F (age, BMI, sex)=60.11 Eta2=0.09 |
| Bener et al. (2006) | Two items of the Self-Reporting Questionnaire (SRQ-20): sleeping badly and feeling tired all the time | Adolescent Dieting Scale (ADS): 8-item scale, included were items on calorie counting, reducing food quantity and meal skipping | Statistically significant relationship between sleeping problems and extreme dieting (p=0.009) | Group comparison (chi-squares and fisher's exact test) for dieters regarding "sleeping badly":  Non-/minimal dieters: n=146 (46.9%) Intermediate dieters: n=84 (37.8%) Extreme dieters: n=35 (58.3%)  p=0.009  Group comparison (chi-squares and fisher's exact test) for dieters regarding "feeling tired all the time":  Non-/minimal dieters: n=156 (50.2%) Intermediate dieters: n=92 (41.4%) Extreme dieters: n=32 (53.3%)  n.s. |
| Berntzen et al. (2021) | Actigraphy, worn for 7 consecutive days (sleep duration, sleep efficiency, sleep onset latency, fragmentation index, sleep initiation, waking)  5 sleep items: sleep duration, sleep need, sleep quality, morning tiredness, daytime tiredness. Calculated sleep debt (sleep need - sleep duration)  Basic Nordic Sleep Questionnaire (BNSQ): 9 items  Morningness-eveningness questionnaire (MEQ): 19 items | Binge Eating Scores (BES). | Sleep duration was negatively correlated with binge-eating in individuals (r = 0.22, P = .019): shorter sleep was associated with higher binge-eating score.  Longer sleep debt was consistently correlated with stronger susceptibility to disinhibited eating in individuals (r = 0.33, P = .0027) and within-pair differences in these behaviors (r = 0.35, P = .031), meaning the correlation holds regardless of genetic and shared environmental influences.  Sleep debt was consistently positively correlated with binge-eating score in individuals (r = 0.34, P < .001) and within-pair differences (r = 0.35, P = .013). | Correlations:  Sleep duration + binge eating: r=-0.22, p=0.02  Sleep debt + binge eating: r=0.34, p<0.001 |
| Birkeland et al. (2012) | Bergen Insomnia Scale: 6 items | Body Image: 4-items ("I would like to change a good deal about my body", "By and large, I am satisfied with my looks", "I would like to change a good deal about my looks", "By and large, I am satisfied with my body") | Not reported | Correlation:  Body image (13 years) + insomnia (30 years): r=-0.04 (n.s.) |
| Blouchou et al. (2024) | Sleep, Circadian Rhythms, and Mood (SCRAM) questionnaire: 15 items | Night Eating Questionnaire (NEQ): 14 items | When NES was defined as an NEQ score ≥25, a marginal trend for an inverse relationship between the total SCRAM and “Good Sleep” was observed. When NES was defined as an NEQ score ≥ 30, a positive association with “morningness” was observed. No other significant associations were detected. | Logistic regressions:  NEQ>=25 vs SCRAM total: OR=0.97 [0.95; 1.00]. p=0.06 NEQ>=25 vs Good Sleep: OR=0.95 [0.91; 0.99], p<0.05 NEQ>=25 vs morningness: OR=1.03 [0.98; 1.07], p=0.21 NEQ>=30 vs SCRAM total: OR=0.99 [0.96; 1.04], p=0.96 NEQ>=30 vs Good Sleep: OR=0.95 [0.89; 1.02], p=0.20 NEQ>=30 vs Morningness: OR=1.08 [1.02; 1.15], p<0.05 |
| Borisenkov et al. (2020) | Munich Chronotype Questionnaire (MCTQ)   Sleep onset on weekdays and free days, sleep onset latency, wake-up time, sleep inertia, sleep duration, mean weekly sleep duration, sleep efficiency | Yale Food Addiction Scale for Children (YFAS-C) and YFAS, Russian version; symptom count (=sum of confirmed symptoms) and a dichotomous measure of food addiction | There were no significant associations between food addiction and chronotype or social jetlag.   Food addiction was associated only with the time of sleep onset on school days. | Food addiction (FA) + sleep onset on weekdays: OR (adjusted)=1.15 [1.04; 1.28], p=0.007 OR(unadjusted)=1.28 [1.16; 1.40]  Group comparison:  Weekdays:  Sleep onset: F=17.7, p=0.00, n2=0.008 Sleep onset latency: F=0.3, p=0.58, n2=0.00 Wake Time: F=3.7, p=0.05, n2=0.002 Sleep inertia: F=2.5, p=0.11, n2=0.001 Sleep duration: F=8.4, p=0.004, n2=0.004  Free days:  Sleep onset: F=3.2, p=0.08, n2=0.001 Sleep onset latency: F=1.0, p=0.31, n2=0.00 Wake Time: F=1.7, p=0.20, n2=0.001 Sleep inertia: F=1.5, p=0.22, n2=0.001 Sleep duration: F=0.2, p=0.69, n2=0.00  General (weekdays/free days):  Chronotype: F=0.9, p=0.34, n2=0.00 Social jetlag: F=0.7, p=0.41, n2=0.00 Average weekly sleep duration: F=6.7, p=0.01, n2=0.003 Sleep efficiency: F=5.0, p=0.03, n2=0.002 |
| Bos et al. (2013) | Two items: "I have difficulty falling asleep", "I wake up many times during the night”  Overall sleep disturbance score index (SDI): summing scores of both items (0-10 range).  4 groups: Good sleepers ("never"/"rarely" difficulties at baseline and follow-up), persistent sleep difficulties ("often"/"very often"/"always" difficulties at baseline and follow-up), onset sleep difficulties (not reporting sleep difficulties at baseline but either at T0 or T1 follow-up), remission sleep difficulties (sleep complaints at baseline but which decreased either at T0 or T1) | Eating Attitudes Test-26 (EAT), Portuguese version: 33 items; High (1 SD above mean) vs low (1 SD below mean) EAT score group | Participants with more severe global disorders of eating at baseline reported significantly more sleep difficulties in initiating and maintaining sleep and in overall sleep disturbances at all stages of the study, when compared to students with less severe disorders. When exploring in more detail which EAT dimensions predicted long-term difficulties in initiating and/or maintaining sleep and persistent insomnia, it was observed that bulimic behaviours were a significant predictor. Our results do not support an association between dietary control and sleep. The fact that dietary control are nowadays frequent (almost “normative”) among the general population, particularly among university students, and not exclusively found in subjects with abnormal eating habits, may explain this result. | Correlations:  "Desire to be thinner" + DIS(T0): r=0.94*  "Desire to be thinner" + DIS(T1): r=0.16**  "Desire to be thinner" + DMS(T1): r=0.13*  "Desire to be thinner" + SDI(T0): r=0.09* "  Desire to be thinner" + SDI(T1): r=0.13*  "Avoid eating when hungry" + DIS(T1): r=0.14*  "Avoid eating when hungry" + DMS(T0): r=0.10*  "Avoid eating when hungry" + DMS(T1): r=0.14*  "Avoid eating when hungry" + SDI(T0): r=0.09*  "Avoid eating when hungry" + SDI(T1): r=0.17**  "Vomiting after meals" + DIS(T0): r=0.15**  "Vomiting after meals" + DIS(T1): r=0.19**  "Vomiting after meals" + DMS(T0): r=0.13**  "Vomiting after meals" + DMS(T1): r=0.24**  "Vomiting after meals" + SDI(T0): r=0.17**  "Vomiting after meals" + SDI(T1): r=0.22** |
| Bruck & Astbury (2012) | One of the 21 "common problems" experienced during the past 12 months: "difficulty sleeping"; 4-point scale (no, rarely, sometimes, often); "often" defined as sleep problems.  Dichotomous variable created as "often difficulty sleeping" vs "no/rarely difficulty sleeping", anyone else omitted | Body weight dissatisfaction: One item: "How much would you to weight now?" (happy as I am, 1-5kg more, over 5kg more, 1-5kg less, 6-10kg less, over 10kg less), coded as "10kg less" vs everything else (dichotomous) | Significant predictors of sleeping difficulties were body weight dissatisfaction, amongst other variables. The association remained significant, even after self-reported depression and anxiety symptoms were added to the model. | Regression:  Sleep difficulties + body weight dissatisfaction: OR=2.05 [1.72-2.44] (n=4,222)  Unadjusted (no depression, anxiety) regression (n=5,456): x2=15.02, p=0.002  Adjusted regression (n=5,456): x2=11.69, p=0.009 |
| Çakır et al. (2018) | Pittsburgh Sleep Quality Index (PSQI) | Night Eating Disorder Scale (NEDS) | Participants with NES had higher PSQI score than non-NES. | NES + PSQI: M=8.4 (SD=2.84) non-NES+PSQI: M=6.2 (SD=2.1) p<0.001 |
| Cecen & Guleken (2023) | Biological Rhythms Interview of Assessment in Neuropsychiatry (BRIAN): 21 items | Yale Food Addiction Scale (YFAS): 27 items | In nonobese subjects, there was no correlation between YFAS and BRIAN subtype parameters. | n.s. correlations not shown in article. |
| Ceylan et al. (2024) | Morningness-Eveningness Questionnaire (MEQ): 19 items, a score between 59 and 86 refers to morning type; 42 and 58 intermediary type and 16 and 41 evening type  Social jetlag (calculated based on sleep timing) | Modified Yale Food Addiction Scale version 2.0 (mYFAS 2.0): 13 items, cut off for "moderate to severe food addiction" = at least four symptoms | Participants with the morning chronotype obtained the lowest score on the food addiction scale, whereas those with the evening chronotype had the highest score. Evening chronotype was the most significant risk factor for food addiction. | Group Comparison:  YFAS:  Evening: M=3.08, SD=3.7 Mixed: M=2.13, SD=2.9 Morning: M=1.88, SD=2.99 F=8.52 p=0.001 η^2^=0.02  Full sample: Regression:  MEQ --> YFAS Coefficient (β)=-0.19, p<0.001  Social Jetlag --> YFAS Coefficient (β)=0.03, p=0.29  Logistic regression (predicting food addiction): Evening type: OR=1.66 [1.13; 2.43], p<0.05 Morning type: OR=0.98 [0.65; 1.48], n.s. Social Jetlag: OR=1.06 [0.96; 1.17], n.s. |
| Chardon et al. (2016) | Sleep Disturbance Scale for Children (SDSC): 26 items (completed by parents)  Pediatric Daytime Sleepiness Scale (PDSS): 8 items (completed by young people) | Children's Eating Attitudes Test (ChEAT), 26-items | Increases in sleep disturbance and daytime sleepiness were independently associated with greater disordered eating attitudes and behaviours. | Correlations:  ChEAT + SDSC: r=-0.26**, p<0.01 ChEAT + PDSS: r=0.37**, p<0.01 |
| Clark (2018) | Sleep record (4 consecutive days Thursday to Sunday)  The Periodicity of Eating- Sleeping (POES-FVS): bedtime, time arising, number of hours slept (POES Sleep sub-score) | Binge Scale (BS): 9 items, score<8 =subclinical binge tendencies, score =8-12 =potential binge problems, score>12 = probable binge disorder; binge eaters defined as "yes" to the question "Do you binge eat?" | Results indicated no significant association between going to bed after midnight and Binge Scale scores.   Participants' binge eating was not significantly associated with bedtimes after midnight. | Chi-Square Test:  Non-Clinical Binge:  86.8% 0-2 nights bedtime after midnight (n=59) 90.4% 3 nights bedtime after midnight (n=103) 90.6% 4 nights bedtime after midnight (n=414)  Clinical Binge:  13.2% 0-2 nights bedtime after midnight (n=9) 9.6% 3 nights bedtime after midnight (n=11) 9.4% 4 nights bedtime after midnight (n=43)  X^2^(639)=0.982, p=0.61 |
| Cooper (2022) | Sleep duration (average nightly hours of sleep for wave 1&2, sleep and wake times during week and non-school days at wave 3, weighted by the number of week and weekend days and averaged over seven days to calculate average hours per night)  Single item for insomnia symptoms (ease of falling asleep and returning to sleep) at wave 1&2 (frequencies: never, rarely, occasionally, often, every day); coded dichotomously (1=insomnia symptoms occasionally, often, every day)  Single item for insufficient sleep at wave 1&2 (getting enough sleep); coded as 1=sufficient sleep and 0=insufficient sleep  Single item for alertness at wave 3 (how often in past seven days fell asleep when they should have been awake); frequencies (never, a few times, almost every day, every day); coded dichotomously (1=a few times, almost every day, every day)  Sleep health: composite sleep health (count of symptoms): insomnia symptoms, insufficient sleep, short sleep (<7 hours) | Those indicating trying to lose weight/stay the same weight were asked to identify engagement in particular restrictive and purging behaviours over past 7 days (wave 1-3): diet, exercise to lose weight, self-induced vomiting, diet pill use, laxative use and/or other (each endorsed REPB = 1) | Results showed that REPBs have significant associations with several facets of poor sleep health; these relationships hold across time, up to one year later. Results provide support for the hypotheses that engagement in REPBs in adolescence would be significantly associated with higher self-reported insomnia symptoms and insufficient sleep, shorter sleep duration, and an overall lower sleep health composite score in adolescence and young adulthood. Regarding specific REPBs across all three Waves, it was found that engagement in dieting, vomiting, diet pill use, and other unspecified restrictive and/or purge behaviors to lose weight were significantly associated with self-report of insufficient sleep, insomnia symptoms, shorter sleep duration, and poorer overall sleep health. | Cross-Sectional: (univariate)  Logistic Regressions: Wave 1:  --> Insomnia Symptoms:  REPB Composite: OR=1.1, SE=0.15, p=0.50 [0.84; 1.44] Dieting to lose weight: OR=1.2, SE=0.09, p=0.02* [1.03; 1.38] Exercising to lose weight: OR=1.1, SE=0.05. p=0.03* [1.01; 1.20] Vomiting to lose weight: OR=0.48, SE=0.23, p=0.13 [0.18; 1.25] Diet pill to lose weight: OR=1.58, SE=0.48, p=0.14 [0.87; 2.90] Laxatives to lose weight: OR=1.00, SE=0.48, p=1.00 [0.39; 2.58] Other to lose weight: OR=1.60, SE=0.24, p<0.01** [1.19; 2.15]  --> Insufficient Sleep:  REPB Composite: OR=1.03, SE=0.04, p=0.49 [0.95; 1.12] Dieting to lose weight: OR=0.88, SE=0.07, p=0.10 [0.75; 1.02] Exercising to lose weight: OR=1.16, SE=0.06, p=0.01* [1.04; 1.28] Vomiting to lose weight: OR=0.41, SE=0.20, p=0.07 [0.16; 1.09] Diet pill to lose weight: OR=0.45, SE=0.11, p<0.01** [0.27; 0.74] Laxatives to lose weight: OR=0.81, SE=0.48, p=0.72 [0.25; 2.60] Other to lose weight: OR=0.68, SE=0.09; p<0.01** [0.52; 0.88]  --> Sleep Duration:  REPB Composite: B=0.02, SE=0.03, p=0.35 [-0.03; 0.07) Dieting to lose weight: B=-0.01, SE=0.05, p=0.86 [-0.10; 0.09] Exercising to lose weight: B=0.05, SE=0.03, p=0.12 [-0.01; 0.11] Vomiting to lose weight: B=-0.44, SE=0.45, p=0.32 [-1.33; 0.44] Diet pill to lose weight: B=-0.24, SE=0.22, p=0.27 [-0.68; 0.19] Laxatives to lose weight: B=0.74, SE=0.36, p=0.04* [0.03; 1.45] Other to lose weight: B=-0.10, SE=0.08; p=0.23 [-0.26; 0.06]  --> Sleep Health:  REPB Composite: B=0.02, SE=0.01, p=0.30 [-0.01; 0.05] Dieting to lose weight: B=0.08, SE=0.03, p<0.01 [0.03; 0.14] Exercising to lose weight: B=-0.02, SE=0.02, p=0.21 [-0.06; 0.01] Vomiting to lose weight: B=0.23, SE=0.21, p=0.28 [-0.19; 0.64] Diet pill to lose weight: B=0.37, SE=0.11, p<0.01** [0.16; 0.58] Laxatives to lose weight: B=-0.05, SE=0.19, p=0.79 [-0.42; 0.32] Other to lose weight: B=0.23, SE=0.05, p<0.01** [0.13; 0.33]  Wave 2:  --> Insomnia Symptoms:  REPB Composite: OR=1.07, SE=0.05, p=0.12 [0.98; 1.17] Dieting to lose weight: OR=1.07, SE=0.10, p=0.45 [0.89; 1.29] Exercising to lose weight: OR=1.09, SE=0.07, p=0.16 [0.97; 1.23] Vomiting to lose weight: OR=1.19, SE=0.52, p=0.69 [0.50; 2.82] Diet pill to lose weight: OR=1.06, SE=0.32, p=0.85 [0.58; 1.93] Laxatives to lose weight: OR=1.41, SE=1.13, p=0.67 [0.29; 6.92] Other to lose weight: OR=1.33, SE=0.22, p=0.09 [0.96; 1.84]  --> Insufficient Sleep:  REPB Composite: OR=0.92, SE=0.04, p=0.03* [0.85; 0.99] Dieting to lose weight: OR=0.74, SE=0.05, p<0.01** [0.64; 0.85] Exercising to lose weight: OR=1.06, SE=0.07, p=0.36 [0.93; 1.21] Vomiting to lose weight: OR=0.39, SE=0.15, p=0.01* [0.19; 0.82] Diet pill to lose weight: OR=0.44, SE=0.11, p<0.01** [0.27; 0.73] Laxatives to lose weight: OR=0.52, SE=0.29, p=0.25 [0.17; 1.59] Other to lose weight: OR=0.73, SE=0.11, p=0.03* [0.54; 0.97]  --> Sleep Duration:  REPB Composite: B=-0.02, SE=0.03, p=0.53 [-0.08; 0.04] Dieting to lose weight: B=-0.07, SE=0.05, p=0.19 [-0.18; 0.04] Exercising to lose weight: B=0.03, SE=0.04, p=0.44 [-0.05; 0.11] Vomiting to lose weight: B=-0.86; SE=0.25; p<0.01** [-1.35; -0.37] Diet pill to lose weight: B=-0.58; SE=0.15, p<0.01** [-0.88; -0.28] Laxatives to lose weight: B=0.14, SE=0.27, p=0.59 [-0.38; 0.67] Other to lose weight: B=0.12, SE=0.10, p=0.24 [-0.08; 0.32]  --> Sleep Health:  REPB Composite: B=0.04; SE=0.02, p=0.04* [<0.01; 0.07] Dieting to lose weight: B=0.11, SE=0.03, p<0.01** [0.05; 0.17] Exercising to lose weight: B=-0.01, SE=0.02, p=0.83 [-0.05; 0.04] Vomiting to lose weight: B=0.46; SE=0.15, p<0.01** [0.17; 0.76] Diet pill to lose weight: B=0.34, SE=0.12, p=0.01* [0.10; 0.58] Laxatives to lose weight: B=0.10, SE=0.27, p=0.71 [-0.44; 0.64] Other to lose weight: B=0.12, SE=0.07, p=0.07 [-0.01; 0.25]  Wave 3:  --> Alertness:  REPB Composite: OR=1.02, SE=0.04, p=0.60 [0.94; 1.11] Dieting to lose weight: OR=1.04, SE=0.08, p=0.57 [0.90; 1.21] Exercising to lose weight: OR=1.00, SE=0.06, p=0.98 [0.88; 1.13] Vomiting to lose weight: OR=1.02, SE=0.11, p=0.88 [0.82; 1.26] Diet pill to lose weight: OR=2.05, SE=0.94, p=0.12 [0.83; 5.07] Laxatives to lose weight: OR=1.30, SE=0.77, p=0.66 [0.40; 4.20] Other to lose weight: -  --> Sleep Duration:  REPB Composite: B=-0.14, SE=0.04, p<0.01 [-0.21; -0.07] Dieting to lose weight: B=-0.10, SE=0.08, p=0.21 [-0.26; 0.06] Exercising to lose weight: B=-0.26, SE=0.05, p<0.01 [-0.36; -0.15] Vomiting to lose weight: B=-0.17, SE=0.10, p=0.10 [-0.38; 0.04] Diet pill to lose weight: B=0.06, SE=0.38, p=0.87 [-0.68; 0.81] Laxatives to lose weight: B=0.69, SE=0.74, p=0.35 [-0.78; 2.17] Other to lose weight: -  Longitudinal:  --> Insomnia (wave 2)  REPB Composite: OR=1.08, SE=0.05, p=0.11 [0.98; 1.18] Dieting to lose weight: OR=1.08, SE=0.08, p=0.33 [0.92; 1.26] Exercising to lose weight: OR=1.00, SE=0.06, p=0.98 [0.88; 1.13] Vomiting to lose weight: OR=0.75, SE=0.39, p=0.58 [0.26; 2.11] Diet pill to lose weight: OR=2.56, SE=1.05, p=0.02 [1.14; 5.75] Laxatives to lose weight: OR=0.40, SE=0.29, p=0.21 [0.10; 1.66] Other to lose weight: OR=0.96, SE=0.19, p=0.83 [0.65; 1.41]  --> Insufficient Sleep (wave 2)  REPB Composite: OR=0.95, SE=0.04, p=0.22 [0.87; 1.03] Dieting to lose weight: OR=0.87, SE=0.07, p=0.10 [0.75; 1.03] Exercising to lose weight: OR=0.97, SE=0.07, p=0.65 [0.85; 1.11] Vomiting to lose weight: OR=0.50, SE=0.31, p=0.26 [0.14; 1.70] Diet pill to lose weight: OR=1.10, SE=0.36, p=0.78 [0.57; 2.11] Laxatives to lose weight: OR=0.77, SE=0.46, p=0.66 [0.24; 2.50] Other to lose weight: OR=0.84, SE=0.18, p=0.42 [0.56; 1.28]  --> Sleep Duration (wave 2):  REPB Composite: B=-0.01, SE=0.03, p=0.79 [-0.07; 0.05] Dieting to lose weight: B=-0.06, SE=0.05, p=0.27 [-0.16; 0.05] Exercising to lose weight: B=0.02, SE=0.04, p=0.69 [-0.07; 0.11] Vomiting to lose weight: B=-0.44, SE=0.31, p=0.16 [-1.05; 0.17] Diet pill to lose weight: B=0.07, SE=0.41, p=0.87 [-0.74, 0.88] Laxatives to lose weight: B=-0.84, SE=0.40, p=0.04* [-1.63; -0.05] Other to lose weight: B=0.06, SE=0.14, p=0.67 [-0.21; 0.33]  --> Sleep Health (wave 2): mistake in original table  REPB Composite: - Dieting to lose weight: B=0.06, SE=0.03, p=0.05 [<0.01; 0.11] Exercising to lose weight: B=<0.01, SE=0.02, p=0.94 [-0.05; 0.04] Vomiting to lose weight: B=0.11, SE=0.14, p=0.43 [-0.17; 0.39] Diet pill to lose weight: B=0.10, SE=0.13, p=0.43 [-0.15; 0.36] Laxatives to lose weight: B=0.03, SE=0.25, p=0.92 [-0.46; 0.52] Other to lose weight: B=-0.01, SE=0.07, p=0.94 [-0.15; 0.14] |
| De Young et al. (2022) | Morningness-Eveningsness Questionnaire (MEQ): Scores 59-86 indicates morning sleep/waketime preference, scores 16-41 evening sleep/waketime preferences  Pittsburgh Sleep Quality Index (PSQI)  Naturalistic light exposure and sleep efficiency: actigraphy assessment (measuring sleep duration and sleep efficiency) | Eating Disorder Examination Questionnaire (EDE-Q)  Night Eating Questionnaire (NEQ): only subscales "Morning Anorexia" and "Evening Hyperphagia" used | Later sleep/waketime preference was associated with more evening hyperphagia and a later peak in light exposure. However, a later peak of light exposure was associated with less evening hyperphagia.  Among individuals with high sleep efficiency, earlier sleep/ waketime preference was associated with less morning anorexia; however, later sleep/ waketime preference was generally associated with greater morning anorexia regardless of sleep efficiency. | Correlations:  MEQ total + EDE-Q Restraint: r=0.05, p=0.58 MEQ total + EDE-Q Eating Concerns: r=-0.08, p=0.34 MEQ total + EDE-Q Shape Concern: r=-0.19*, p=0.02 MEQ total + EDE-Q Weight Concern: r=-0.18*, p=0.03 MEQ total + EDE-Q Global: r=-0.12, p=0.13 MEQ total + NEQ Morning Anorexia: r=-0.32***, p<0.001 MEQ total + NEQ Evening Hyperphagia: r=-0.19*, p=0.02  Regression:  Sleep duration + morning anorexia: B=0.004, SE=0.002 [0.0002; 0.007], t=2.09, p=0.04 |
| EE & Gan, 2022 | Pittsburgh Sleep Quality Index (PSQI): 19 items  Morningness and Eveningness Questionnaire (MEQ): 19 items | Night Eating Questionnaire (NEQ): 14 items | No association between night eating syndrome & sleep quality | Univariate Regression:  Night eating predicting sleep quality: OR=4.10 [0.91; 18.44], p=0.07  Multivariate Regression:  Night eating predicting sleep quality: OR=2.36 [0.46; 12.02], p=0.30  Group comparison (chi-square test):  Good sleep + night eater: n=2 (1.4%)  Poor sleep + night eater: n=13 (5.5%)  p=0.09 |
| Eid et al. (2022) | Pittsburgh Sleep Quality Index (PSQI): 19 items | Night Eating Questionnaire (NEQ): 14 items | Cross-sectional:  Night eating was associated with high PSQI score. After controlling for the effects of covariates, night-eating was associated with poor subjective sleep quality. Night eating was related to longer SOL (sleep onset latency), habitual sleep efficiency (SE), more sleep disturbances (SD) and daytime dysfunction (DD).   Longitudinal:   Night eating did not predict PSQI score at T1, after controlling for the effect of PSQI at T0. | Regression:  Cross-sectional:  NEQ -> PSQI: B=0.14***, SE=0.03, B=0.28, t=4.21, p<0.001 NEQ -> SSQ: B=0.03**/0.02*, SE=0.008/0.008, B=0.20, p=0.007/0.01 NEQ -> SOL: B=0.05***, SE=0.01, B=0.30, t=3.82, p<0.001 NEQ -> SE: B=0.03*, SE=0.01, B=0.20, p=0.01 NEQ -> SD: B=0.02*, SE=0.006, B=0.26, t=3.45, p=0.001 NEQ -> SM: B=0.01/0.01, SE=0.009-0.009, B=0.11, t=1.35, p=0.18/0.19 NEQ -> DD: B=0.02*/0.02, SE=0.008/0.008, B=0.16, p=0.03/0.03  Longitudinal:  NEQ -> PSQI: B=0.03, SE=0.04, B=0.06, t=0.86, p=0.39 NEQ -> SSQ: B=0.01, SE=0.008, B=0.11, t=1.42, p=0.16 NEQ -> SOL: B=-0.007, SE=0.01, B=-0.05, t=-0.65, p=0.52 NEQ -> sleep duration: B=0.02, SE=0.01, B=0.12, t=1.46, p=0.15 NEQ -> SD: B=0.001, SE=0.006, B=0.02, t=0.19, p=0.85 NEQ -> DD: B=0.005, SE=0.009, B=0.04, t=0.50, p=0.62 |
| Farhangi (2019) | Pittsburgh Sleep Quality Index (PSQI), Persian version: 19 items | Night Eating Questionnaire (NEQ): 14 items | Adolescents with NES, had higher PSQI.  Among PSQI components, habitual sleep efficiency score, daytime dysfunction score and sleep disturbances score among NES group were significantly higher compared with non-NES group. Interestingly, the prevalence of night eating syndrome in poor sleepers was substantially higher compared with good sleepers (P = 0.03). | NES: n=11, non-NES: n=73  PSQI total score:  NES: M=9.00, SD=2.56 Non-NES: M=6.78, SD=2.52 p=0.02  SSQ:  non-NES: M=1.18, SD=0.87 NES: M=1.65, SD=0.02 p=0.12  SOL:  non-NES: M=1.27, SD=0.78 NES: M=0.95, SD=0.78 p=0.24  Sleep duration:  non-NES: M=1.09, SD=0.34 Non-NES: M=0.59, SD=0.11 p=0.19  Habitual SE:  non-NES: M=1.54, SD=1.12 NES: M=0.72, SD=0.13 p=0.04  SD:  non-NES: M=1.54, SD=0.52 NES: M=1.12, SD=0.59 p=0.02  Use of SM:  non-NES: M=0.45, SD=0.15 NES: M=0.41, SD=0.08 p=0.78  Daytime dysfunction:  non-NES: M=1.90, SD=0.70 NES: M=1.32, SD=0.76 p=0.02 |
| Fernández-Argüelles et al. (2022) | Sleep Activity: ActiGraph accelerometers (7 complete days) and sleep diary: sleep efficiency (total sleep time divided by total time in bed, in %) | Body Image Discrepancy:   Figure Rating Scale, adapted for Spanish context | Negative relationships were observed between sleep efficiency and perceived body image, as well as between sleep efficiency and body image discrepancy. High scores of both perceived body image and body image discrepancy were related to reduced sleep efficiency.  Sleep efficiency predicted both perceived body image and body image discrepancy. | Correlations:  Perceived b.i. + sleep efficiency: r=-0.20 (p<0.01) Ideal b.i. + sleep efficiency: r=-0.07 (n.s.) B.i. discrepancy + sleep efficiency: r=-0.15 (p<0.05)  Linear Regression (predicted by sleep efficiency):   Perceived b.i.: B=-0.22 (CI: -.011;-0.02), SE=0.02, p=0.003 (only without physical self-concept +BMI+PWB added, then n.s.)  Ideal b.i.: B=-0.04 (CI: -0.04;0.03), SE=0.02, p=0.59  B.i. discrepancy: B=-0.18 (CI: -0.09; -0.12), SE=0.02, p=0.01 (only without physical self-concept +BMI+PWB added, then n.s.) |
| Figueroa et al. (2024) | Sleep Disturbance bank form the PROMIS: 6 items (sleep quality and frequency of sleep disturbances over past week) | Eating Disorders Examination Questionnaire Short (EDE-QS): 12 items | Significant correlation between disordered eating and sleep disturbances. | Correlation:  Disordered eating + sleep disturbance: r=0.31, p<0.001 |
| Fordsham et al. (2019) | Morningness-Eveningness Questionnaire (MEQ): 19 items | Eating Attitudes Test - Short Version (EAT-8): 8 items |  | Correlation:  Morningness and Eating Attitudes: r=0.00 (n.s.) |
| Gallant et al. (2013) | Sleep duration: "How many hours of sleep do you usually get per night?" (<7h = short sleeper) | Currently trying to lose weight (TLW): "Are you currently trying to lose weight?"  Previously lost weight (PWL): "Have you ever deliberately lost weight (>10Ibs)?"  Affirmative answers to both (RWL)  Restrictive eating (from TFEQ): "Do you voluntarily stop eating before have emptied your plate to restrain your caloric intake?" -> often/always vs never/rarely  Overeating (from TFEQ): "Do you have trouble to stop eating before you have emptied your plate, even if you are no longer hungry?" -> often/always vs never/rarely | Individuals who reported PWL had increased odds of reporting restrained and overeating tendencies, particularly the former, and of being a short sleeper, compared to individuals who did not report PWL. Individuals who reported RWL behaviours had an additive increased risk of reporting all psycho-behavioural measures compared to all other individuals. | Short Sleeper:  Not trying to lose weight (n=2047) vs trying (n=1022): OR=1.16 [0.92; 1.5], n.s.  No previous weight loss (n=2053) vs previous weight loss (n=1016): OR=1.30 [1.0; 1.6], p<0.05  No repeated weight loss (n=2492) vs repeated weight loss (n=577): OR=1.39 [1.1; 1.8]. p<0.05 |
| Gundogdu & Yildirim (2023) | Scale for Outcomes in Parkinson's Disease (SCOPA) Sleep Scale: 12 items | The Night Eating Questionnaire (NEQ): 14 items | When the scores of girls and boys with NEQ scores below and above 25 were compared, the SCOPA sleep scale was higher in adolescent girls and boys with NEQ scores of 25 and above; when all adolescents were divided into NEQ groups (without dividing them by gender), scores on the SCOPA sleep scale were higher in adolescents with NEQ scores of 25 and above.   SCOPA total scores were associated with NEQ>25 for the total sample and boys, but not adolescent girls. | Group comparison:  SCOPA-Night Sleep:  NEQ<25 (n=113): M=6.95, SD=3.15 NEQ>=25 (n=54): M=9.41, SD=3.76 F=19.63, p<0.001  SCOPA-Daytimes Sleepiness: NEQ<25 (n=113): M=2.51, SD=2.41 NEQ>=25 (n=54): M=5.73, SD=4.71 F=34.15, p<0.001  SCOPA-Total: NEQ<25 (n=113): M=9.46, SD=4.69 NEQ>=25 (n=54): M=15.14, SD=7.16 F=37.52, p<0.001  Linear regression (predicting NEQ above 25):  SCOPA-total (all): β=0.30, t=4.16, p<0.001 [0.01; 0.03] |
| Hafstad et al. (2013) | Sleep pattern in childhood (3 items from the BCL=Behaviour Checklist): difficulties falling asleep at bedtime, nightly awakenings, unwillingness to sleep alone | Eating Attitudes Test (EAT-12) | All childhood measure were pooled across the three measurement points (1.5, 2.5, 4.5 years).  Children rated high on sleep problems before age five were significantly more likely to score high on self-reported eating problems at age 16. | Bivariate Correlation:  EAT (16 y) + sleep problems: r=0.11**, p<0.01  Regression:  Sleep problems -> EAT (16 y.):  B=0.14, beta=0.102*, SE=0.08, p=0.049 |
| Hao et al. (2023) | Pittsburgh Sleep Quality Index (PSQI): 19 items | Stunkard visual graph to measure body dissatisfaction | Sleep quality was a significant predictor of body dissatisfaction. | Regression sleep quality --> body dissatisfaction:  β=0.19, p<0.01 (reported in text, β=0.16 reported in table) |
| Hasan et al. (2023) | Pittsburgh Sleep Quality Index (PSQI): 19 items  Morningness-Eveningness Questionnaire (MEQ): 19 items; score <=30 definite evening, score 31-41 moderate evening, score 59-69 moderate morning, score >=70 definite morning, categorised into three categories (definite and moderate merged) | Eating Attitude Test (EAT-26): 26 items, score >20 disturbed eating patterns | The ED risk was independent of the chronotype category. However, there was a significant association between ED risk and sleep quality (p = 0.016), as those with ED risk had higher reports of poor sleep quality (86.1%) than good sleep quality (13.9%) compared to those without ED risk (77.8% and 22.2%, respectively).  EAT score was positively associated with sleep quality score. This remained to be significant after adjusting for age and sex. The sleep quality score presents to be the best predictor for the EAT score. | Chi-Square Group Comparison:  Disordered eating attitudes (n=209):  Morningness: n=20 (9.6%) Intermediate: n=115 (55%) Eveningness: n=74 (35.4%)  Normal eating attitudes (n=343):  Morningness: n=32 (9.3%) Intermediate: n=198 (57.7%) Eveningness: n=113 (32.9%)  p=0.82  Disordered eating attitudes:  Poor sleep: n=180 (86.1%) Good sleep: n=29 (13.9%)  Normal eating attitudes:  Poor sleep: n=267 (77.8%) Good sleep: n=76 (22.2%)  p=0.02  Regression (EAT-26 =DV):  Sleep quality: B=0.87, p<0.001 [0.49; 1.26] Chronotype: B=1.25, p=0.09 [-0.02; 0.27]  Adjusted (age, sex):  Sleep quality: B=0.87, p<0.001 [0.48; 1.26] Chronotype: B=0.11, p=0.14 [-0.03; 0.25] |
| Hirai et al. (2022) | Athens Insomnia Scale (AIS), Japanese version: 8 items | Eating Attitudes Test-16: 26 items, cut-off score >=20 indicates potential ED | Adolescents with ED symptoms were found to be vulnerable to reduced daytime functioning potentially related to sleep problems. The between-group comparisons in this study did not find an association between sleep problems at night and potential ED, but specifically identified daytime functional problems related to sleep (potentially due to age). | Group Comparisons:  Sleep Initiation:  Non-ED: n=201 (no problem), n=125 (slightly), n=44 (markedly), n=11 (very) ED symptoms: n=6 (no problem), n=7 (slightly), n=2 (markedly), n=2 (very)  z=1.56, p=0.12, p(adj)=0.19, r(effect)=0.08  Night Awakening:  Non-ED: n=324 (no problem), n=45 (minor), n=11 (considerable), n=1 (serious) ED symptoms: n=13 (no problem), n=3 (minor), n=1 (considerable), n=0 (serious)  z=0.97, p=0.33, p(adj)=0.38, r(effect)=0.05  Early Morning Awakening:  Non-ED: n=314 (not earlier), n=58 (a little earlier), n=7 (markedly earlier), n=2 (much earlier) ED symptoms: n=12 (not earlier), n=2 (a little earlier), n=1 (markedly earlier), n=2 (much earlier)  z=1.5, p=0.13, p(adj)=0.18, r(effect)=0.08  Total Sleep Duration:  Non-ED: n=172 (sufficient), n=165 (slightly insufficient), n=38 (markedly insufficient), n=6 (very insufficient) ED symptoms: n=9 (sufficient), n=3 (slightly insufficient), n=2 (markedly insufficient), n=3 (very insufficient)  z=0.34, p=0.73, p(adj)=0.73, r(effect)=0.02  Overall Sleep Quality:  Non-ED: n=190 (satisfactory), n=160 (slightly unsatisfactory), n=25 (markedly unsatisfactory), n=6 (very unsatisfactory) ED symptoms: n=7 (satisfactory), n=4 (slightly unsatisfactory), n=4 (markedly unsatisfactory), n=2 (very unsatisfactory)  z=1.71, p=0.09, p(adj)=0.18, r(effect)=0.09  Sense of Wellbeing during the day***:  Non-ED: n=313 (normal), n=61 (slightly decreased), n=2 (markedly decreased), n=5 (very decreased) ED symptoms: n=8 (normal), n=4 (slightly decreased), n=3 (markedly decreased), n=2 (very decreased)  z=3.98, p<0.001, p(adj)<0.001, r(effect)=0.20  Daytime Functioning:  Non-ED: n=315 (normal), n=47 (slightly decreased), n=14 (markedly decreased), n=5 (very decreased) ED symptoms: n=10 (normal), n=2 (slightly decreased), n=2 (markedly decreased), n=3 (very decreased)  z=2.84, p=0.005, p(adj)=0.02, r(effect)=0.14  Sleepiness During Day:  Non-ED: n=103 (none), n=207 (mild), n=59 (considerable), n=12 (intense) ED symptoms: n=4 (none), n=4 (mild), n=7 (considerable), n=2 (intense)  z=2.18, p=0.03, p(adj)=0.08, r(effect)=0.11  Correlations:  EAT-26 Total:  Nocturnal: r=0.27, p(adj)<0.001 Daytime: r=0.29, p(adj)<0.001  Dieting Score:  Nocturnal: r=0.25, p(adj)<0.001 Daytime: r=0.25, p(adj)<0.001  Bulimia Score:  Nocturnal: r=0.16, p(adj)=0.002 Daytime: r=0.23, p(adj)<0.001  Oral Control Score:  Nocturnal: r=0.14, p(adj)=0.006 Daytime: r=0.14, p(adj)=0.005 |
| Johnson (2020) | Sleep intervention: sleep deprived (50% of their typical reported sleep duration) vs habitual sleep (100% of their reported sleep duration)  Pittsburgh Sleep Quality Index (PSQI): 19 items   Consensus Sleep Diary (CSD): 9 items | Eating Pathology Symptom Inventory (EPSI): 45 items  Night Eating Questionnaire (NEQ): 14 items  Eating Disorder Behaviour Form (EDBF): binge eating, overaeting, loss of control eating, self-induced vomiting, misuse of diuretics, exercise, skipping meals, fasting, urges to engage in these behaviours | Effects of the experimental group were not significant and there were no significant group by time interactions for disordered eating urges. For weight-control urges, there were no significant effects of time, group or their interaction for urges to vomit, restrict, or exercise.   Univariate ANCOVAs, controlling for pre-meal urges, examined group differences in disordered eating and weight-control urges at follow up. There were no significant group differences at follow-up for urges to overeat and urges for loss of control eating. Similarly, there were no significant group differences at follow-up for urges to vomit, restrict, and exercise.   The sleep-deprived group was not more likely to report engaging in a disordered-eating behaviours compared to the habitual group. | Pearson's chi-square (experimental group differences among disordered eating behaviours at follow up):  Any Behaviour: 35% (n=14) total, 15% (n=6) sleep deprived, 20% (n=8) habitual  No Behaviour: 65% (n=26) total, 35% (n=14) sleep deprived, 30% (n=12) habitual   χ^2^=0.44, p=0.51 |
| Kandeger et al. (2018) | Morningness-Eveningness Questionnaire (MEQ): 19 items; classification as morning type (score 59-86), evening type (16-41) and neither (42-58)  Insomnia Severity Index (ISI): 7 items | Night Eating Questionnaire (NEQ): 14 items, items 13, 15 and 16 not used for total score  Eating Attitudes Test (EAT): 40 items, cut-off=30 | There was a statistically significant correlation between scores of all scales, except between MEQ and EAT.   Statistically significant differences were found between the groups in terms of MEQ, ISI, and NEQ scores. Evening types had higher insomnia and night eating scores than the other two chronotypes (p < 0.01). | Correlations:  MEQ + NEQ: r=-0.29, p<0.01 MEQ + EAT: r=-0.03, n.s. ISI + NEQ: r=0.38, p<0.01 ISI + EAT: r=0.23, p<0.01  Group comparison:  NEQ:  Evening type: M=18.22, SD=4.96 Neither type: M=15.09, SD=5.16 Morning type: M=13.41, SD=4.53 F=14.51, p<0.001, partial eta squared=0.07, post hoc A>B=C  EAT:  Evening type: M=11.00, SD=7.91 Neither type: M=11.49, SD=9.40 Morning type: M=11.61, SD=7.07 F=0.09, p=0.92, partial eta squared=0.00, post hoc - |
| Kandeger et al. (2019) | Morningness-Eveningness Questionnaire (MEQ): 19 items; classified into 3 categories (16-41=evening, 42-58=neither, 59-86=morning)  Insomnia Severity Index (ISI) | Yale Food Addiction Scale (YFAS): 25 items; in order for food addiction to be specified, participant had to meet =>3 symptoms and indicate clinically significant impairment | There was a strong correlation between all scale scores except correlations between BMI and sleep parameters.  Statistically significant differences were found between the groups in the MEQ, ISI, YFAS and BIS-11-SF scores excluding BMI. Evening-type individuals had more insomnia, impulsivity and food addiction scores than the other two chronotypes (p<0.01).  Insomnia score was a risk factor for food addiction. Having morning or evening types on MEQ were no risk factor. | Correlations:  MEQ + YFAS: r=-0.10, p<0.01 ISI + YFAS: r=0.14, p<0.01  Group Comparison (LSD multiple group comparison test):  YFAS:  Evening type: M=3.64, SD=1.53 Neither type: M=3.44, SD=1.45 Morning type: M=3.06, SD=1.35 F=8.53, p<0.001, partial eta squared=0.01, C>A>B  Logistic regression (predicting food addiction):  ISI: OR=2.24 [1.58; 3.17], B=0.81, p<0.001 Morning type: OR=0.98 [0.63; 1.53], B=-0.02, p=0.94 Evening type: OR=1.12 [0.74; 1.69], B=0.11, p=0.59 |
| Kiltie et al. (2024) | PROMIS sleep disturbance - short form: 8 items (authors mentioned error in response scale)  Number of hours spent out of bed (time to bed - time of waking) | Eating Pathology Symptoms Inventory (EPSI): 45 items | Only supplementary material relevant | Correlations (all n.s. p>.001):  Wake time:  Body Dissatisfaction: r=0.05 (Mon), -0.02 (Wed), -0.05 (Sat) Binge Eating: r=0.14 (Mon), 0.05 (Wed), 0.09 (Sat) Cognitive restraint: r=-0.07 (Mon), -0.10 (Wed), -0.13 (Sat) Purging: r=0.11 (Mon), 0.05 (Wed), -0.11 (Sat) Restricting: r=-0.06 (Mon), -0.01 (Wed), 0.06 (Sat) Excessive exercise: r=0.01 (Mon), -0.07 (Wed), 0.02 (Sat)  Bed time:  Body Dissatisfaction: r=-0.08 (Mon), -0.06 (Wed), 0.02 (Sat) Binge Eating: r=0.01 (Mon), -0.01 (Wed), 0.10 (Sat) Cognitive restraint: r=-0.07 (Mon), -0.04 (Wed), -0.05 (Sat) Purging: r=-0.02 (Mon), -0.08 (Wed), -0.03 (Sat) Restricting: r=0.02 (Mon), 0.08 (Wed), 0.15 (Sat) Excessive exercise: r=0.07 (Mon), 0.02 (Wed), 0.05 (Sat) |
| Krawitz (2011) | Pittsburgh Sleep Quality Index (PSQI) | Bulimic Investigatory Test, Edinburgh (BITE): 33 items  The Eating Disorders Examination Questionnaire (EDE-Q) | Compensatory Behaviours: A significant interaction between the mixed cluster and compensatory behaviors was found that among good sleep, mixed cluster individuals displayed greater compensatory behaviors than the healthy cluster with good sleep.   Shape concern: There were no significant effects for the dummy coded independent variables, Time 1 sleep or significant interactions.  Weight concern: A regression analysis was conducted to assess the effects of Time 1 weight concern and sleep on Time 2 weight concern. The overall model was significant, and accounted for a 41.6%> of the total variability in weight concern scores. Time 1 weight concern was significant (b=0.62, p< .001), with higher scores at Time 1 predicting higher scores at Time 2. There were no other significant effects or interactions.   BITE severity: There was no main effect for the moderator as an independent variable. There were no significant interactions.  Objective Bulimic Episodes (OBE): No additional main effects other than overall regression model with main effect for Time 1 OBE. | Predicting compensatory behaviours:  Step 1: Sleep T1: B=0.00, SE=0.05, p=0.94, t=0.06   Step 2: Sleep T1: B=0.07, SE=0.08, p=0.40, t=0.83 Sleep x (healthy vs mixed): B=-0.39, SE=0.13, p<0.001, t=-2.96 Sleep x (healthy vs restraint): B=0.12, SE=0.12, p=0.32, t=0.98  Predicting shape concerns:  Step 1: Sleep T1: B=-0.00, SE=0.01, p=0.66, t=-0.42  Step 2: Sleep T1: B=0.00, SE=0.02, p=0.97, t=0.02 Sleep x (healthy vs mixed): B=-0.03, SE=0.03, p=0.43, t=-0.78 Sleep x (healthy vs restraint): B=0.00, SE=0.03, p=0.96, t=0.04  Predicting weight concerns:  Step 1: Sleep T1: B=-0.01, SE=0.01, p=0.29, t=0.29  Step 2: Sleep T1: B=-0.00, SE=0.02, p=0.97, t=-0.02 Sleep x (healthy vs mixed): B=-0.04, SE=0.04, p=0.29, t=-1.05 Sleep x (healthy vs restraint): B=-0.01, SE=0.04, p=0.70, t=-0.38  Predicting BITE-severity:  Step 1: Sleep T1: B=-0.00, SE=0.05, p=0.86, t=-0.17  Step 2: Sleep T1: B=0.02, SE=0.08, p=0.79, t=0.26 Sleep x (healthy vs mixed): B=-0.15, SE=0.13, p=0.24, t=-1.17 Sleep x (healthy vs restraint): B=0.03, SE=0.12, p=0.77, t=0.28  Predicting objective bulimic episodes:  Step 1: Sleep T1: B=0.01, SE=0.16, p=0.67, t=0.41  Step 2: Sleep T1: B=0.00, SE=0.02, p=0.96, t=0.04 Sleep x (healthy vs mixed): B=-0.02, SE=0.04, p=0.49, t=-0.69 Sleep x (healthy vs restraint): B=0.03, SE=0.03, p=0.29, t=1.06 |
| Lauer et al. (2021) | Insomnia Severity Index (ISI): 7 items | Weight control behaviours: Intention about weight ("I am trying to lose weight", "I am trying to gain weight", "I am trying to stay the same weight", "I am not trying to do anything about my weight in any way") | The MANCOVA result indicated that insomnia differed across the four WCBs.   Follow-up post hoc tests with Bonferroni adjustments revealed significant univariate effects for insomnia.  Specifically, compared to those who were trying to stay the same weight or not trying to do anything about their weight, the girls who were trying to lose weight had significantly greater insomnia symptoms (d = .43 and .43, respectively). | MANCOVA (comparison between WCBs):  Insomnia:   Stay the same weight (n=74): M=5.6, SD=4.91 Lose weight (n=85): M=7.88, SD=5.68 Gain weight (n=20): M=6.35, SD=5.46 Do nothing (n=144): M=5.64, SD=4.78 F=3.78, p=0.01, partial eta square=0.03 |
| Lee & Suh (2018) | Disturbing Dream Nightmare Severity Index Questionnaire (DDNSI): 5 items   Insomnia Severity Index (ISI): 7 items | Night Eating Questionnaire (NEQ): 17 items | Nightmares were positively correlated with night eating. | Correlation:  Nightmares + night eating: r=0.29, p<0.01  Insomnia + night eating: r=0.54, p<0.01 |
| Lee et al. (2021) | 6-item sleep disturbance short form from the Patient-Reported Outcomes Measurement Information System item bank (sleep quality over past week from very poor to very good and frequency of sleep disturbance in the past week from not at all to very much) | Eating Disorder Examination Questionnaire Short (EDE-QS): 12 items (reference past 7 days) | Only correlation relevant | Correlation:  Sleep disturbance + Disordered Eating: r=0.33, p<0.001 |
| Lew et al. (2020) | Sleep duration, binary (<8h or >=8h): "On an average school night, how many hours of sleep do you get?" | Body image: two dichotomous variables (whether adolescents felt they were the right weight and whether they were trying to either gain or lose weight) | Individuals reporting short sleep duration had significantly increased odds of being categorized into any of the high-risk classes, compared to their peers reporting sufficient sleep. In particular, students who reported short sleep duration had odds of being in the high risk for all behaviors class that were 2.47 times as great (95% CI: [1.96, 3.11]) as individuals reporting sufficient sleep. | Adjusted Odds (adjusted for age, biological sex, grade, sexual identity, race, BMI percentile) of Class Membership for Students Reporting Short Sleep Duration:  High risk for poor body image: OR=1.32 [1.17; 1.49] |
| Lin et al. (2020) | Insomnia Severity Index (ISI): 7 items; different levels of insomnia (0-7=absence, 8-14=sub-threshold, 15-21=moderate, 22-28=severe) | Eating Attitude Test-26 (EAT-26): 26 items  Yale Food Addiction Scale for Children (YFAS-C); all converted into dichotomous items (symptom count scoring) | The effects of insomnia may be exacerbated by psychological problems and can lead to excess weight via unhealthy eating when the individual cannot get sleep, and where they possibly consume energy-rich foods. | Correlations:  EAT + Insomnia: r=0.22, p<0.01 YFAS + Insomnia: r=0.30, p<0.01 |
| Liu et al. (2022) | Athens Insomnia Scale (AIS) | Eating Attitude Test (EAT-19), Chinese version: 19 items | Results determined that each of the three mental health factors and insomnia were all positively associated with the abnormal eating attitudes total scale and subscales and that all of these associations were large or medium size statistical effects | Correlations:  Insomnia + Dieting: r=0.36, p<0.01 Insomnia + Bulimia and food preoccupation: r=0.33, p<0.01 Insomnia + Awareness of food contents: r=0.24, p<0.01 Insomnia + compensatory behaviour: r=0.21, p<0.01 Insomnia + total EAT-19: r=0.38, p<0.01 |
| Lo et al. (2011) | Sleep problems as one indicator of "psychological distress" within "psychosocial health problems"; 2 items (adapted from Uppsala Sleep Inventory): "Do you find it hard to fall asleep or stay asleep?" and "Do you have nightmares?"; rated as dichotomous (never/seldom vs sometimes/always) | Weight misperception (to measure body image disturbances): correct perception (normal weight + "just right" perception), misperceived fatness (normal weight + "too fat"/"fat"), misperceived thinness (normal weight + "too thin"/"thin") | In the fully adjusted model, girls who misperceived themselves as fat or very fat were associated with all psychosocial health problems. Boys who misperceived themselves as very thin or fat were significantly associated with all psychosocial health problems, except for having nightmares.   Boys who misperceived themselves as thin or very fat had higher risks of having bad/average appetite, being sometimes/always sleepless at night and having no/average confidence in getting along with friends, but those who misperceived themselves as very fat also had higher risk of sometimes/always having nightmares.  In general, greater ORs were observed for misperceived fatness than thinness in girls, but similar ORs were observed in boys | Logistic regression:  Boys (reference "just right"):  Sleepless + very thin: OR=1.32 [1.03; 1.68], p=0.03 Sleepless + thin: OR=1.22 [1.07; 1.38], p=0.002 Sleepless + fat: OR=1.16 [1.05; 1.28], p=0.004 Sleepless + very fat: OR=1.75 [1.43; 2.13], p<0.001  Nightmares + very thin: OR=1.10 [0.82; 1.49], p=0.50 Nightmares + thin: OR=1.10 [0.96; 1.27], p=0.20 Nightmares + fat: OR=1.11 [0.99; 1.24], p=0.09 Nightmares + very fat: OR=1.84 [1.38; 2.47], p<0.001  Girls (reference "just right"):  Sleepless + very thin: OR=1.26 [0.94; 1.68], p=0.10 Sleepless + thin: OR=0.95 [0.82; 1.11], p=0.60 Sleepless + fat: OR=1.37 [1.25; 1.51], p<0.001 Sleepless + very fat: OR=1.83 [1.48; 2.25], p<0.001  Nightmares + very thin: OR=1.09 [0.82; 1.46], p=0.50 Nightmares + thin: OR=1.09 [0.92; 1.29], p=0.30 Nightmares + fat: OR=1.25 [1.13; 1.38], p<0.001 Nightmares + very fat: OR=1.78 [1.52; 2.08], p<0.001  PAF analysis:  Boys:  Sleepless + misperceived thinness: 15.6 [6.1; 26.5)  Sleepless + misperceived fatness: 14.4 [6.8; 23.2)  Nightmares + misperceived thinness: 4.1 [0; 10.7]  Nightmares + misperceived fatness: 7.9 [2.6; 13.1]  Girls:  Sleepless + misperceived thinness: 0.6 [0; 9.6)  Sleepless + misperceived fatness: 29.7 [20.8; 40.1)  Nightmares + misperceived thinness: 6.2 [0; 15.8]  Nightmares + misperceived fatness: 18.4 [10.8; 26.6] |
| Lombardo et al. (2010) | Insomnia Severity Index (ISI) | Disordered Eating Questionnaire (DEQ): assessing the presence and intensity of eating restriction  Contour Drawing Rating Scale (CDRS): pictorial measure of body dissatisfaction | Emotional suppression predicts insomnia severity and Emotional suppression and Insomnia severity both predicts Restrictive eating. | Not reported |
| Lombardo et al. (2013) | Sleep Disorder Questionnaire (SDQ): classification into 3 groups (Good Sleepers (GS)=no sleep problems, persistent insomnia (PI)=clinically significant symptoms consistent with diagnostic criteria, subthreshold insomnia (SI)=symptoms of insomnia with weekly frequency, persistence or consequences lower than those indicated by diagnostic criteria)  Insomnia Severity Index (ISI) | Disordered Eating Questionnaire (DEQ): 24 items  Eating Attitude Test (EAT-26), Italian version: 26 items | As regards the eating-disorder symptoms, the group Persistent Insomnia (PI)/Disordered Eating (DE) showed higher scores than both the PI and AC (asymptomatic control) groups on both questionnaires (p < 0.001) while PI and AC do not differ from each other on both questionnaires (DEQ: p=0.302; EAT: p=0.992). | Group differences on severity of disordered eating (DEQ):  AC (asymptomatic controls): M=8.33, SD=4.40 PI: M=14.77, SD=7.20 PI/DE: 56.54, SD=15.99 F(2, 36)=81.76 p<0.001  Group differences on severity of disordered eating (EAT):  AC (asymptomatic controls): M=2.00, SD=2.31 PI: M=2.38, SD=2.33 PI/DE: 25.26, SD=12.91 F(2, 36)=38.95 p<0.001  Comparison of PI and AC group: DEQ: p=0.30, EAT: p=0.99 |
| Lombardo et al. (2014) | Sleep Disorders Questionnaire (SDQ): three categories of sleep quality (good sleepers (GS)=no sleep problems, persistent syndromal insomnia (PI)=clinically significant symptoms of insomnia on basis of diagnostic criteria, subthreshold insomnia (SI) = symptoms of insomnia with frequency, persistence or consequences lower than those indicated by diagnostic criteria)  The Insomnia Severity Index (ISI) | Disordered Eating Questionnaire (DEQ): 24 items | Results of the ANCOVA evidenced that the DEQ scores differed significantly across sleep groups. Scheffé post-hoc tests found that the PI and SI groups were not significantly different (p= 0.97), whereas they both differed from the GS group (p< 0.001).   The distribution of ED groups was significantly different across the sleep groups (χ2(2) = 49.016; P < 0.001). | Group comparisons (ANOVA):  Disordered Eating Questionnaire:  Good Sleepers: M=11.07, SD=11.41 Subthreshold insomnia: M=20.51, SD=16.90 Persistent insomnia: M=26.98, SD=18.99 F(2, 1016)=40.82 p=0.001  Scheffé post-hoc test: PI + SI: p=0.97 PI + GS and SI + GS: p<0.001  ED group comparison (symptomatic vs asymptomatic) regarding sleep groups: χ2(2) = 49.016; P < 0.001 |
| Lundgren et al. (2008) | 24-hour sleep records (sleep and wake times)  Morningness-Eveningness (ME) Scale  Pittsburgh Sleep Questionnaire (PSQ)  Epworth Sleepiness Scale (ESS) | Night Eating Questionnaire (NEQ): classified as NES-positive if they reported evening hyperphagia (consumption of 25% or more of daily food intake after supper until final awakening next morning) and/or nocturnal awakening and ingestion of food three or more times per week | Although there were no differences between groups on the Epworth Sleepiness Scale, suggesting no significant excessive daytime sleepiness, the sleep of night eaters was impaired as evidenced by the consistently elevated scores in comparison to controls on the Pittsburgh Sleep Questionnaire (PSQ).  Night Eaters had significantly lower scores on the Morningness-Eveningness (ME) scale, indicating that they considered themselves to function better in the latter part of the day than the controls. All PSQ and ME analyses remained significantly different after controlling for experiment-wise error. | Group comparison:  PSQ:  Subjective Sleep Quality:  NES: M=2.1, SD=0.7 Control: M=0.6, SD=0.6 t(1, 38)=7.1, p<0.001  Sleep latency:  NES: M=1.6, SD=1.0 Control: M=0.5, SD=0.5 t(1, 37)=5.5, p<0.001  Sleep duration:  NES: M=1.3, SD=0.9 Control: M=0.6, SD=0.5 t(1, 37)=2.7, p=0.007  Habitual sleep efficiency:  NES: M=1.1, SD=1.3 Control: M=0.0, SD=0.0 t(1, 36)=4.6, p<0.001  Sleep disturbance:  NES: M=1.4, SD=0.5 Control: M=0.8, SD=0.4 t(1, 37)=3.9, p<0.001  Use of sleep medication:  NES: M=1.0, SD=1.1 Control: M=0.1, SD=0.4 t(1, 38)=3.8, p=0.001  Daytime dysfunction:  NES: M=1.3, SD=1.0 Control: M=0.3, SD=0.5 t(1, 38)=4.0, p<0.001  Global:  NES: M=10.2, SD=3.7 Control: M=2.9, SD=1.5 t(1, 35)=8.6, p<0.001  ESS:  NES: M=6.8, SD=4.9 Control: M=5.7, SD=3.5 t(1, 39)=1.1, p=0.27  ME:  NES: M=32.6, SD=9.8 Control: M=40, SD=7.7 t(1, 39)=2.7, p=0.009 |
| Manasse et al. (2022) | Actigraphy (14 days), marking time beginning to fall asleep and time awakening: collected continuously and stored in 30s epochs (Total Sleep Time (TST) restricted to night sleep)  Sleep diary protocols: time in bed, time out of bed | Ratings for LOC eating (“While you were eating, did you feel a sense of loss of control?” “While you were eating, did you feel that you could not stop eating once you had started?” “While you were eating, did you feel like you could not resist eating?” “While you were eating, did you feel like a car without brakes, you just kept eating and eating?”). The four items assessing LOC eating were summed to form a total score (range = 4–20) | A significant interaction between within‐person negative affect and within‐person TST (total sleep time) emerged in the GLMM predicting LOC eating severity (B= −0.01, SE < 0.01, p = 0.025). Following nights characterised by lower TST (relative to one's average), higher momentary negative affect was related to higher subsequent LOC eating severity scores. Conversely, this association was attenuated following nights characterised by higher TST. | Correlations:  LOC eating + total sleep time (TST): r=-0.01 (n.s.)  GLMM (for LOC eating):  TST (between): B=-0.03, SE=0.03, t=-1.10, p=0.27 [-0.09; 0.02]  TST (within): B=-0.01, SE=0.01, t=-1.33, p=0.19 [-0.03; 0.01]  TST (between) x NA (within): B<-0.01, SE<0.01, t=-0.97, p=0.33 [-0.01; <0.01]  TST (within) x NA (within): B=-0.01, SE<0.01, t=-2.25, p=0.03 [-0.01; <-0.01] |
| Mason & Heron (2016) | Sleep difficulties (assessed at T1): number of nights participants had trouble falling asleep in the past four weeks | Binge eating symptoms in the past week (assessed at baseline, T0): two items, rated as "yes" or "no" ("Have you eaten so much in a short period that you would have been embarrassed if others had seen you do it?" and "Have you been afraid to start eating because you thought you wouldn't be able to stop or control your eating?")  Weight perception (assessed ay T1): “What do you think about your weight” (very underweight, slightly underweight, about the right weight, slightly overweight, very overweight) | Objective overeating was prospectively associated with more sleep difficulties. Loss of control eating was prospectively associated with more sleep difficulty. After controlling for depressive symptoms (at T0), objective overeating was no longer associated with sleep difficulty, however loss of control eating was still related to sleep difficulties. | Frequencies by overeating and loss of control eating (LOC):  Sleep difficulties (sd):  Yes vs no overeating (OE): X2=13.79, p=0.008  No OE: sd 0: n=5731 sd <1: n=2288 sd 1-2: n=2143 sd 3-4: n=971 sd 5+: n=907  Yes OE: sd 0: n=334 sd <1: n=142 sd 1-2: n=158 sd 3-4: n=82 sd 5+: n=71  Yes vs no loss of control eating: X2=46.52, p<0.001  No LOC: sd 0: n=5956 sd <1: n=2383 sd 1-2: n=2234 sd 3-4: n=1018 sd 5+: n=929  Yes LOC: sd 0: n=108 sd <1: n=46 sd 1-2: n=67 sd 3-4: n=36 sd 5+: n=48  Logistic regression (predicting sleep difficulty=sd):  Overeating (no depression):  <1 sd: OR=1.05 [0.79; 1.40] 1-2 sd: OR=1.11 [0.84; 1.47] 3-4 sd: OR=1.45 [1.02; 2.06] 5+ sd: OR=1.32 [0.86; 2.03]  Overeating (depression included):  <1 sd: OR=0.99 [0.75; 1.32] 1-2 sd: OR=1.00 [0.75; 1.33] 3-4 sd: OR=1.18 [0.84; 1.68] 5+ sd: OR=1.05 [0.68; 1.62]  Loss of control eating (no depression):  <1 sd: OR=1.58 [0.94; 2.66] 1-2 sd: OR=1.64 [0.99; 2.70] 3-4 sd: OR=1.47 [0.80; 2.70] 5+ sd: OR= 3.13 [1.81; 5.41]  Loss of control eating (depression included):  <1 sd: OR=1.44 [0.85; 2.41] 1-2 sd: OR=1.40 [0.86; 2.69] 3-4 sd: OR=0.59 [0.80; 2.05] 5+ sd: OR=2.22 [1.28; 3.85] |
| Matias et al. (2020) | Adapted questions from the Global School-Based Student Health Survey (GSHS) questionnaire: "How often were you unable to sleep at night because something really bothered you?" (yes=sometimes, mostly, always or no=never, rarely) | "Body weight dissatisfaction" (from the COMPAC study): self-assessment scale, dichotomised as "satisfied" vs "dissatisfied"  Attitudes towards body weight: "What are you doing in relation to your body weight?" (trying to lose weight, trying to gain weight, trying to maintain weight)  Both combined: "satisfied" (regardless of attitudes), "dissatisfied and not controlling weight", "dissatisfied and trying to lose weight", "dissatisfied and trying to gain weight", "dissatisfied and trying to maintain weight" | Adolescents who were dissatisfied and were not controlling their weight were 1.21 (95% CI: 1.11-1.32) more likely to present impairments in sleep in relation to adolescents satisfied with their weight.   Those who were dissatisfied and were trying to lose (OR: 1.16, 95%CI: 1.08-1.25) and gain (OR: 1.43, 95%CI: 1.28-1.61) weight had also higher odds. And those who were dissatisfied and were trying to maintain their weight presented 1.43 odds of having sleep impairments. | Regression (predicting sleep impairments):  Crude:  Body satisfaction: OR=1 Body dissatisfaction (not controlling): OR=1.89 [1.73; 2.07] Body dissatisfaction (lose): OR=1.84 [1.73; 1.97] Body dissatisfaction (gain): OR=2.24 [2.00; 2.50] Body dissatisfaction (maintain): OR=1.93 [1.66; 2.24]  Model 1 (adj. gender, age, mother's schooling, ethnicity, occupational status):  Body satisfaction: OR=1 Body dissatisfaction (not controlling): OR=1.8 [1.64; 1.97] Body dissatisfaction (lose): OR=1.7 [1.59; 1.82] Body dissatisfaction (gain): OR=2.15 [1.92; 2.40] Body dissatisfaction (maintain): OR=1.95 [1.68; 2.27]  Model 2 (adj. loneliness):  Body satisfaction: OR=1 Body dissatisfaction (not controlling): OR=1.21 [1.11; 1.32] Body dissatisfaction (lose): OR=1.18 [1.09; 1.27] Body dissatisfaction (gain): OR=1.42 [1.27; 1.60] Body dissatisfaction (maintain): OR=1.39 [1.18; 1.62]  Model 3 (adj. gender, age, mother's schooling, ethnicity, occupational status, loneliness):  Body satisfaction: OR=1 Body dissatisfaction (not controlling): OR=1.21 [1.11; 1.32] Body dissatisfaction (lose): OR=1.16 [1.08; 1.25] Body dissatisfaction (gain): OR=1.43 [1.28; 1.61] Body dissatisfaction (maintain): OR=1.43 [1.22; 1.68] |
| Meule et al. (2014) | Morningness-Eveningness Questionnaire-reduced (rMEQ): 5 items | Night Eating Questionnaire (NEQ): 14-items | Scores on the NEQ were weakly negatively correlated with the rMEQ (r =-.20, p <.001), indicating stronger eveningness preference with increasing night eating severity. | Correlation:  NEQ + rMEQ: r=-0.20, p<0.001 |
| Mori et al. (2009) | Sleeping behaviours: waking-up time, time of sleep, number of hours slept | Perceived body image: "Do you perceive yourself as thin, normal or fat?" in comparison to actual BMI: 'underestimated group' (underestimated weight), 'normal group' (normal weight) and 'overestimated group' (overestimated weight) | Respondents who went to bed after 23.00 hours and who slept <7 h were approximately 1.4-fold more likely to have an overestimated weight body image than those who went to bed between 22:00 and 23:00 or slept 7–8.5 h (OR 1.38, 95%CI: 1.18–1.60, P < 0.001; and OR 1.40, 95%CI: 1.18–1.65, P < 0.001, respectively). | Logistic Regression (predicting body image perception): Crude/Adjusted  Normal vs Underestimated (all n.s.)  Wake-up time:  Before 6.30: n=930 normal, n=106 under OR=0.85 [0.65; 1.10]/OR=0.82 [0.64-1.06] 6.30-7.00: n=1123 normal, n=151 under OR=1 After 7.00: n=338 normal, n=45 under OR=0.99 [0.70; 1.41]/OR=1.04 [0.73; 1.49]  Bedtime:  Before 22.00: n=331 normal, n=53 under OR=1.23 [0.88; 1.71]/OR=1.15 [0.82; 1.62] 22.00-23.00: n=1187 normal, n=155 under OR=1 After 23.00: n=880 normal, n=92 under OR=0.80 [0.61; 1.05]/OR=0.83 [0.63; 1.09]  Sleeping time:  <7h: n=511 normal, n=50 under OR=0.74 [0.54; 1.03]/OR=0.79 [0.57; 1.09] 7-8.5h: n=1542 normal, n=203 under OR=1 >8.5 h: n=354 normal, n=51 under OR=1.09 [0.79; 1.52]/OR=1.04 [0.74; 1.45]  Normal vs overestimated  Wake-up time:  Before 6.30: n=910 normal, n=465 over OR=1.10 [0.94; 1.28]/OR=1.11 [0.95-1.29] 6.30-7.00: n=1104 normal, n=514 over OR=1 After 7.00: n=330 normal, n=178 over OR=1.16 [0.94; 1.43]/OR=1.11 [0.89; 1.37]  Bedtime:  Before 22.00: n=328 normal, n=156 over OR=1.14 [0.92; 1.42]/OR=1.17 [0.94; 1.46] 22.00-23.00: n=1167 normal, n=487 over OR=1 After 23.00: n=855 normal, n=515 over OR=1.44* [1.24; 1.68]/OR=1.38* [1.18; 1.60], p<0.05  Sleeping time:  <7h: n=496 normal, n=319 over OR=1.46* [1.23; 1.73]/OR=1.40* [1.18; 1.65] 7-8.5h: n=1513 normal, n=667 over,  OR=1 >8.5 h: n=350 normal, n=176 over OR=1.14 [0.93; 1.40]/OR=1.15 [0.94; 1.41]  Adjusted variables: menstruation (on sleep), TV time (on sleep) |
| Nagata et al. (2021) | Proxies of sleep disturbances: trouble falling asleep ("Over the past 4 weeks, how often did you have trouble falling asleep?"), trouble staying asleep ("Over the past 4 weeks, how often did you have trouble staying asleep through the night? For example, you woke up several times at night or woke up earlier than you planned to?"); 4 response options for both (never in past 4 weeks, less than once a week, 1 or 2 times a week, 3 or 4 times a week, 5 or more times a week); at baseline, both questions were combined | Eating behaviour: those indicating wanting to lose weight or stay the same weight were asked "Which of the following things did you do during the past 7 days to lose weight or stay the same weight?" (fasting/skipping meals, making yourself throw up, laxatives, diuretics, taking weight loss pills), of which the first was defined as restrictive eating behaviour, the rest compensatory  Overeating or loss of control eating: present when individuals self-reported that they have "eaten so much in a short period of time that they would have been embarrassed if others had seen them do it" or who indicated they had "been afraid to start eating because they thought they wouldn't be able to stop or control their eating" in the past 7 days | While controlling for demographics, proxies of an ED diagnosis and reports of all disordered eating behaviors were all associated with both self-reported sleep disturbance outcomes  In the context of depression, incidence rate ratios for DEBs were attenuated and not independently significantly associated with self-reported sleep disturbance outcomes, with the exception of restrictive eating behaviors and difficulty falling asleep whose association remained statistically significant.   When participants with an ED diagnosis proxy were excluded from the analyses, the association between restrictive eating behaviors and difficulty falling asleep was attenuated but other associations were similar. | Prospective associations between indices of disordered eating and sleep disturbance constructs, adjusted for demographic and socioeconomic covariates:  Restrictive eating behaviours -> falling asleep: IRR=1.18 [1.08; 1.28], p<0.001  After additionally adjusting for depression: Restrictive eating behaviours -> falling asleep: IRR=1.10 [1.01; 1.20], p<0.05  Compensatory behaviours -> falling asleep: IRR=1.15 [1.03; 1.30], p<0.01  After additionally adjusting for depression: Compensatory behaviours -> falling asleep: IRR=1.08 [0.96; 1.21], n.s.  Overeating/loss of control eating -> falling asleep: IRR=1.21 [1.09; 1.34], p<0.001  After additionally adjusting for depression: Overeating/loss of control eating -> falling asleep: IRR=1.08 [0.97; 1.21], n.s. ------------------------------------------- Restrictive eating behaviours -> staying asleep: IRR=1.11 [1.02; 1.20], p<0.01  After additionally adjusting for depression: Restrictive eating behaviours -> staying asleep: IRR=1.05 [0.97; 1.14], n.s.  Compensatory behaviours -> staying asleep: IRR=1.16 [1.04; 1.30], p<0.01  After additionally adjusting for depression: Compensatory behaviours -> staying asleep: IRR=1.11 [0.99; 1.25], n.s.  Overeating/loss of control eating -> staying asleep: IRR=1.19 [1.09; 1.31], p<0.001  After additionally adjusting for depression: Overeating/loss of control eating -> staying asleep: IRR=1.10 [1.00; 1.22], n.s. ------------------------------------------- Excluding participants with potential ED diagnosis:  Restrictive eating behaviours -> falling asleep: IRR=1.14 [1.05; 1.24], p<0.01  After additionally adjusting for depression: Restrictive eating behaviours -> falling asleep: IRR=1.07 [0.99; 1.17], n.s.  Compensatory behaviours -> falling asleep: IRR=1.14 [1.01; 1.28], p<0.05  After additionally adjusting for depression: Compensatory behaviours -> falling asleep: IRR=1.08 [0.96; 1.21], n.s.  Overeating/loss of control eating -> falling asleep: IRR=1.21 [1.09; 1.34], p<0.001  After additionally adjusting for depression: Overeating/loss of control eating -> falling asleep: IRR=1.09 [0.97; 1.21], n.s. ------------------------------------------- Restrictive eating behaviours -> staying asleep: IRR=1.10 [1.01; 1.20], p<0.05  After additionally adjusting for depression: Restrictive eating behaviours -> staying asleep: IRR=1.05 [0.96; 1.14], n.s.  Compensatory behaviours -> staying asleep: IRR=1.17 [1.04; 1.31], p<0.05  After additionally adjusting for depression: Compensatory behaviours -> staying asleep: IRR=1.13 [1.00; 1.27], n.s.  Overeating/loss of control eating -> staying asleep: IRR=1.20 [1.09; 1.32], p<0.001  After additionally adjusting for depression: Overeating/loss of control eating -> staying asleep: IRR=1.11 [1.00; 1.23], n.s. |
| Natale et al. (2008) | Reduced version of Morningness-Eveningness Questionnaire (MEQr): 5 items, 3 labels (4-10=evening, 11-18=intermediate, 19-25=morning) | Eating Disorder Inventory (EDI-2): 91 questions | Only in the patient group (not included in this review) was a significant negative correlation observed between EDI-2 subscales and MEQr. | Not reported (n.s.) |
| Nolan & Geliebter (2016) | Pittsburgh Sleep Quality Index (PSQI): 19 items | Night Eating Questionnaire (NEQ): 14 items  Yale Food Addiction Scale (YFAS) | Students: There were strong positive correlations between NEQ global scores and sleep quality.  Community: There were strong significant positive correlations between NEQ global and sleep quality. | Correlations:  Students: NEQ+PSQI: r=0.45, p<0.001 YFAS+PSQI: r=0.21, p<0.001  Community: NEQ+PSQI: r=0.64, p<0.001 YFAS+PSQI: r=0.43, p<0.001  Regression (PSQI -> NEQ global):  Student: beta=0.26, t=4.29, p<0.001  Community: beta=6.95, t=0.00, p<0.001 |
| Nolan & Geliebter (2019) | Pittsburgh Sleep Quality Index (PSQI): 19 items | Night Eating Diagnostic Questionnaire (NEDQ), symptom checklist: 22 items | The regression model predicting sleep quality from all proposed diagnostic criteria was highly statistically significant in the community sample, F(9, 456) = 52.69, p =.000 (adjusted R-squared = .50), and in the student sample, F(9, 240) = 10.75, p =.000 (adjusted R-squared = .26). Poor sleep quality was associated in both samples with morning anorexia, low mood in evening, and sleep problems. However, evening hyperphagia, need to eat in order to sleep, awareness of night eating, and distress/impairment were associated with poor sleep quality only in the community group. | Tetrachoric correlation (night eating diagnostic criteria):  Sleep problems + evening hyperphagia: r=0.22, p<0.01  Regression of each psychopathology measure on NES diagnostic criteria for the community sample:  PSQI:  Evening hyperphagia: β=0.11. t=3.04, p=0.003 Nocturnal eating: β=-0.02, t=-0.45, p=0.66 Depressed mood: β=0.15, t=4.45, p<0.001 Must eat to sleep: β=0.09, t=2.11, p=0.04 Evening urge to eat: β=-0.02, t=-0.46, p=0.64 Sleep problems: β=0.53, t=15.22, p<0.001 Morning anorexia: β=0.11, t=3.31, p=0.001 Awareness: β=0.11, t=2.35, p=0.02 Distress/impairment: β=0.10, t=2.65, p=0.008 ------------------------------------------- Student sample:  PSQI:  Evening hyperphagia: β=-0.02, t=-0.38, p=0.70 Nocturnal eating: β=-0.11, t=-1.58, p=0.12 Depressed mood: β=0.13, t=2.30, p=0.02 Must eat to sleep: β=0.01, t=0.13, p=0.89 Evening urge to eat: β=0.06, t=1.06, p=0.29 Sleep problems: β=0.16, t=8.33, p<0.001 Morning anorexia: β=0.15, t=2.68, p=0.008 Awareness: β=0.00, t=-0.06, p=0.96 Distress/impairment: β=0.01, t=0.24. p=0.81 |
| Park et al. (2020) | Appropriate sleep (7-8h) measured as part of general health-related behaviour assessment | Subjective body image perception: "What do you think of your body image?" (severely thin/ slightly thin, average/normal, slightly overweight/severely overweight); adjusted by BMI (BIOP=body image over perception, BICP=body image correct perception, BIUP=body image under perception) | The BIOP group was the top with 41.7% in terms of sleeping below 7 h. Also, 28.9% said they sleep between 7 to 8 h—the recommended sleeping hours—which was less compared to the other groups. Also, the BIOP group aged 64 or below showed less sleeping time compared to their counterparts. | Health-related behaviours of different body image perceptions (aged <=65):  BICP:  >7h/day: n=1515 (37.2%) >8h/day: n=1271 (32.2%) <=8 h/day: n=1246 (30.6%)  BIUP:  >7h/day: n=269 (36.1%) >8h/day: n=230 (35%) <=8 h/day: n=190 (28.9%)  BIOP:  >7h/day: n=1071 (41.7%) >8h/day: n=762 (28.9%) <=8 h/day: n=745 (29.4%)  Rao-Scott X2=14.2, p=0.007  Health-related behaviours of different body image perceptions (aged >65):  BICP:  >7h/day: n=450 (42.9%) >8h/day: n=244 (24.2%) <=8 h/day: n=311 (32.8%)  BIUP:  >7h/day: n=236 (43.2%) >8h/day: n=93 (20.5%) <=8 h/day: n=185 (36.3%)  BIOP:  >7h/day: n=121 (43.2%) >8h/day: n=71 (25%) <=8 h/day: n=92 (31.8%)  Rao-Scott X2=2.73, p=0.61 |
| Parker et al. (2022 & 2020) | Actigraphy monitors to assess sleep (within-person shifts in nightly sleep duration (h/night), bedtime, waketime, and midpoint were computed using the absolute difference between a person's duration/time of the previous night's sleep and their 2-week average sleep duration/time) | Ecological Momentary Assessment (EMA) for loss of control eating (LOC): six items (e.g., I felt a sense of loss of control, I could not stop eating once I started); severity computed by averaging items within persons and within days  LOC-Severity: When an eating episode was reported, participants rated the degree to which they experiences LOC-eating during that snack/meal, using adapted items from the EDE: "How much did you lose control during this eating episode?", "Did you feel that you could not keep yourself from eating?", "Did you feel that you could not stop eating once you started?", "During the eating episode you just finished, how much did you feel a sense of loss of control?", "How upset or distressed are you about how much you just ate?", "How much did you feel driven to eat?" | When accounting for all facets of sleep, weekly sleep duration (est. ß = −0.31, p = .004), sleep midpoint (est. ß = −0.47, p = .01), and wake time (est. ß = 0.49, p = .01) were associated with greater LOC-eating severity. Specifically, youth who had shorter average sleep duration, an earlier average sleep midpoint, and, on average, woke up later reported greater LOC-eating severity during the EMA period. Shifts in daily facets of sleep were not significantly related to subsequent LOC-eating severity (est. ß = −0.01 – 0.02, p = .11 – .92). Results remained significant after adjusting for all covariates. | Article:  Unadjusted GLMM predicting LOC-eating:  Sleep duration (weekly):   β=-0.31, SE=0.11, t=-2.86, p=0.004 [-0.52; -0.10]  Sleep duration (daily):  β=-0.003, SE=0.01, t=-0.42, p=0.67 [-0.02; 0.01]  Sleep onset (weekly):  β=0.03, SE=0.04, t=0.60, p=0.55 [-0.06; 0.11]  Sleep onset (daily):  β=-0.01, SE=0.01, t=-1.09, p=0.27 [-0.03; 0.01]  Midpoint (weekly):  β=-0.47, SE=0.18, t=-2.68, p=0.01 [-0.82; -0.13]  Midpoint (daily):  β=0.02, SE=0.01, t=1.59, p=0.11 [-0.004; 0.04]  Waketime (weekly):  β=0.49, SE=0.18, t=2.75, p=0.01 [0.14; 0.84]  Waketime (daily):  β=0.001, SE=0.01, t=0.10, p=0.92 [-0.02; 0.02] |
| Prieto et al. (2012) | Morningsness-Eveningness Scale for Children (MESC): 10 items | Veçú et santé perçue de l´adolescent (VSP-A): body image items (39 items) | Morning-type adolescents obtained higher scores than evening-type adolescents on body image. | Evening type: M=69.99, SD=29.04 (N=342) Neither type: M=71, SD=28.06 (N=879) Morning type: M=76.25, SD=25.89 (N=379)  Post-hoc comparison:  Morning-type adolescents obtained higher scores than evening-type on body image: F(2, 1593)=3.83, p<0.05 |
| Ramos et al. (2023) | Insomnia: sporadic (never/sometimes/rarely), yes (often/always) | Extreme Weight Loss Behaviour (EWLB): "Did you vomit or take laxatives to lose weight or avoid gaining weight?" (yes, no), "Have you taken any medicine, formula, or other weight loss product without medical follow-up?" (yes, no), at least of these questions affirmed was considered presence of EWLB  Body Image Satisfaction: satisfied (satisfied/very satisfied), indifferent, dissatisfied (dissatisfied/very dissatisfied)  Attitude towards weight: no attitude, trying to lose or maintain weight, trying to gain weight | Having insomnia (PR=1.91, CI=1.48; 2.46) was significantly and positively associated with EWLB. | Group comparison:  No EWLB: Insomnia never/sometimes/rarely: n=1899 (89%) Insomnia often/always: n=238 (79.3%)  EWLB: Insomnia never/sometimes/rarely: n=234 (11%) Insomnia often/always: n=62 (20.7%)  p<0.001  Crude prevalence values: PRCrude=1.91 (1.48; 2.46), p<0.001 |
| Reche-García et al. (2018) | Athens Insomnia Scale (EAI-8): 8 items | Exercise Dependence (EDS-R): 21 items, divided into risk of dependency (RD= scores >5 in three criterions), symptomatic but independent (SID=scores of 3 to 4 in three or more criterions or obtain scores of 5 to 6 combined with scores of 3 to 4 in three criterions, without reaching the requirements to be included in the RD group), and asymptomatic independent (AID=minimum score of one to two in at least three criterions, without reaching the requirements of incorporation in the SID group) | The results suggest that exercise dependence maintains an interdependent relationship with sleep problems (p<0.01), but this did not happen with sport experience. | Correlations:  Exercise Dependence + Insomnia: r=0.21, p<0.01 |
| Reichborn-Kjennerud et al. (2004) | Five-item version (SCL-5) of the Hopkins Symptom Check List (SCL)-25 was used to assess symptoms of anxiety and depression = screening for insomnia, sleep medication use | Binge eating: "Have you lost control while eating and were unable to stop before you had eaten too much?" (at least twice a week, 1-4 times a month, seldom or never)  Inappropriate compensatory behaviours: "Have you used 1) vomiting or 2) laxatives or 3) fasting or 4) excessive physical exercise to control your weight?" (at least twice a week, 1-4 times a month, seldom or never)  Binge eating in the absence of compensatory behaviours = a feeling of loss of control at least 2 times a week and the absence of regular use of any of the above-mentioned inappropriate compensatory behaviours. | Use of sleep medication was only significantly associated with BE before adjusting for BMI. In women, higher rates of insomnia were associated with BE even after controlling for BMI. | Regression (before and after adjusting for BMI, BE as independent variable):  Insomnia:   Men (unadjusted):  No BE: 5.5% BE: 8.7% OR=1.55 [0.77; 3.24], p=0.21  Men (adjusted for BMI):  OR=1.58 [0.77; 3.24], p=0.21  Use of sleep medication:  Men (unadjusted):  No BE: 0.3% BE: 2.1% OR=7.43 [1.56; 35.48], p=0.01  Men (adjusted for BMI):  OR=5.32 [0.94; 30.10], p=0.06  Insomnia:  Women (unadjusted):  No BE: 5.7% BE: 10.6% OR=1.90 [1.23; 2.94], p=0.004  Women (adjusted for BMI):  OR=1.97 [1.25; 3.13], p=0.004  Use of Sleep Medication:  Women (unadjusted):  No BE: 0.9% BE: 2.8% OR=0.65 [0.09; 4.71], p=0.67  Women (adjusted for BMI):  OR=0.44 [0.06; 3.27], p=0.42 |
| Riccobono et al. (2019) | Morningness Eveningness Questionnaire (MEQ): 19 items; 3 types (morning=59-86, intermediate=42-58, evening=16-41) | Night Eating Questionnaire (NEQ): 15 items | The data indicate that MEQ and NEQ scores are significantly inversely correlated (r=-0.157; p=0.006). 58.3% of the participants who reached the criteria for NES, received low scores on the MEQ; the results highlight a significant association between evening type and NES. | Correlation:  MEQ+NEQ: r=-0.16, p=0.006  Morning type:  NES: M=2, SD=16.7 No-NES: M=25, SD=8.7 p=0.03  Intermediate type:  NES: M=3, SD=25 No-NES: M=203, SD=70.2  Eveningness type:  NES: M=7, SD=58.3 No-NES: M=61, SD=21.1 |
| Riccobono et al. (2020) | Morningness Eveningness Questionnaire (MEQ), Italian version: 19 items, divided into morning type (score 59-86), intermediate type (score 42-58), and evening type (score 16-41) | Night Eating Questionnaire (NEQ), Italian version: 15 items | The data indicated that NEQ and MEQ scores are significantly inversely correlated. The 36.7% of the participants who reached the criteria for NES, obtained low scores on the MEQ (χ2=10.41, p<.005). | Correlation:  NEQ+MEQ: r=-0.22, p<0.01  The 36.7% of the participants who reached the criteria for NES, obtained low scores on the MEQ (χ2=10.41, p<.005). |
| Richardson et al. (2024) | Children's Morningness-Eveningness Scale (MESC): 10 items  School-night sleep duration: "How many hours sleep do you usually get each night on a school night?" (pre-filled drop down from 0-12)  Pediatric Daytime Sleepiness Scale (PDSS): 8 items, higher scores reflect greater daytime sleepiness (for longitudinal analyses reverse-scored) | Children's Eating Attitude Test (ChEAT): 26 items | There were small to moderate correlations between sleep variables and psychological symptoms within each wave; the strength of the associations was strongest for daytime sleepiness.  Worse adolescent sleep was directly related to worsening eating disorder symptoms, whereas eating disorder symptoms did not predict changes in adolescent sleep over time. | Correlations:  All correlations with T0:  T0:  M/E + ED: r=-0.08, n.s. Sleep Duration + ED: r=-0.23, p<0.044 Daytime Sleepiness +ED: r=0.21, p<0.044  T1:  M/E + ED: r=-0.05, n.s. Sleep Duration + ED: r=-0.20, p<0.044 Daytime Sleepiness +ED: r=0.14, p<0.044  T2:  M/E + ED: r=-0.07, p<0.044 Sleep Duration + ED: r=-0.21, p<0.044 Daytime Sleepiness +ED: r=0.17, p<0.044  T3:  M/E + ED: r=-0.01, n.s. Sleep Duration + ED: r=-0.13, p<0.044 Daytime Sleepiness +ED: r=0.11, p<0.044  T4:  M/E + ED: r=0.05, n.s. Sleep Duration + ED: r=-0.10, p<0.044 Daytime Sleepiness +ED: r=0.10, p<0.044  T5:  M/E + ED: r=-0.06, n.s. Sleep Duration + ED: r=-0.17, p<0.044 Daytime Sleepiness +ED: r=0.16, p<0.044  All correlations with T1:  T1:  M/E + ED: r=-0.04, n.s. Sleep Duration + ED: r=-0.12, p<0.044 Daytime Sleepiness +ED: r=0.16, p<0.044  T2:  M/E + ED: r=-0.01, n.s. Sleep Duration + ED: r=-0.05, n.s. Daytime Sleepiness +ED: r=0.12, p<0.044  T3:  M/E + ED: r=0.01, n.s. Sleep Duration + ED: r=-0.10, p<0.044 Daytime Sleepiness +ED: r=0.17, p<0.044  T4:  M/E + ED: r=0.03, n.s. Sleep Duration + ED: r=-0.06, n.s. Daytime Sleepiness +ED: r=0.13, p<0.044  T5:  M/E + ED: r=-0.05, n.s. Sleep Duration + ED: r=-0.08, n.s. Daytime Sleepiness +ED: r=0.20, p<0.044  All correlations with T2:  T2:  M/E + ED: r=-0.04, p<0.044 Sleep Duration + ED: r=-0.13, p<0.044 Daytime Sleepiness +ED: r=0.26, p<0.044  T3:  M/E + ED: r=-0.06, n.s. Sleep Duration + ED: r=-0.08, n.s. Daytime Sleepiness +ED: r=0.20, p<0.044  T4:  M/E + ED: r=-0.06, n.s. Sleep Duration + ED: r=-0.10, p<0.044 Daytime Sleepiness +ED: r=0.22, p<0.044  T5:  M/E + ED: r=-0.11, p<0.044 Sleep Duration + ED: r=-0.13, p<0.044 Daytime Sleepiness +ED: r=0.25, p<0.044  All correlations with T3:  T3:  M/E + ED: r=-0.11, p<0.044 Sleep Duration + ED: r=-0.10, p<0.044 Daytime Sleepiness +ED: r=0.31, p<0.044  T4:  M/E + ED: r=-0.05, p<0.044 Sleep Duration + ED: r=-0.12, p<0.044 Daytime Sleepiness +ED: r=0.28, p<0.044  T5:  M/E + ED: r=-0.11, p<0.044 Sleep Duration + ED: r=-0.09, n.s. Daytime Sleepiness +ED: r=0.30, p<0.044  All correlations with T4:  T4:  M/E + ED: r=-0.10, p<0.044 Sleep Duration + ED: r=-0.14, p<0.044 Daytime Sleepiness +ED: r=0.34, p<0.044  T5:  M/E + ED: r=-0.13, p<0.044 Sleep Duration + ED: r=-0.17, n.s. Daytime Sleepiness +ED: r=0.31, p<0.044  All correlations with T5:  T5:  M/E + ED: r=-0.18, p<0.044 Sleep Duration + ED: r=-0.10, n.s. Daytime Sleepiness +ED: r=0.36, p<0.044  Regression (longitudinal):  T0 Sleep -> T1 ED: β=-0.07, SE=0.03, p<0.05  T1 Sleep -> T2 ED: β =-0.07, SE=0.03, p<0.05  T2 Sleep -> T3 ED: β =-0.07, SE=0.02, p<0.05  T3 Sleep -> T4 ED: β =-0.07, SE=0.02, p<0.05  T4 Sleep -> T5 ED: β =-0.07, SE=0.02, p<0.05  T0 ED -> T1 Sleep: β =-0.002, SE=0.02, n.s.  T1 ED -> T2 Sleep: β =-0.002, SE=0.02, n.s.  T2 ED -> T3 Sleep: β =-0.002, SE=0.02, n.s.  T3 ED -> T4 Sleep: β =-0.002, SE=0.03, n.s.  T4 ED -> T5 Sleep: β =-0.002, SE=0.03, n.s. |
| Rosenbaum et al. (2023) | "In the past 7 days, on average, how many hours of sleep did you get per night?" (open-ended) | Body Appreciation Scale 2 (BAS-2): 10 items  Appearance Evaluation (7 items) and Appearance Orientation (12 items) subscales of the  Multidimensional Body Self Relations Questionnaire (MBSRQ) | Hours of sleep at night were positively and significantly correlated with body appreciation and appearance evaluation, indicating that more sleep was associated with more appreciation for one’s body and with more positive feelings regarding one’s appearance. Sleep was not related to appearance orientation; therefore, appearance orientation was not included as a dependent variable in subsequent models examining sleep and body image. | Correlations:  H sleep + Body Appreciation: r=0.22, p<0.001 H sleep + Appearance Evaluation: r=0.20, p<0.001 H sleep + Appearance Orientation: r=-0.03, n.s.  Group comparison:  Body Appreciation:  Lower sleep (<6h): M=3.47, SD=0.88 Higher sleep (>6h): M=3.79, SD=0.88 t=2.36, p=0.02, d=0.36  Appearance Evaluation:  Lower sleep (<6h): M=3.14, SD=0.90 Higher sleep (>6h): M=3.51, SD=0.86 t=2.82, p=0.005, d=0.43  Appearance Orientation:  Lower sleep (<6h): M=3.60, SD=0.60 Higher sleep (>6h): M=3.63, SD=0.51 t=0.28, p=0.78, d=0.04 |
| Sahlan et al. (2023) | Farsi-Insomnia Severity Index (F-ISI) | Farsi-Eating Disorder Examination Questionnaire - 6th Edition (F-EDE-Q): combined four subscales to examine global ED symptoms over the past 28 days; an additional item on binge eating (having loss of control over eating and consuming a large amount of food); self-induced vomiting and laxative misuse merged | Overall, college students with ED symptoms (i.e., global ED symptoms, binge eating, purging) endorsed varying severity (subthreshold, moderate, and severe) of insomnia and the distribution of insomnia severity also varied across ED symptoms. All variables were significantly positively associated with one another.  Insomnia was independently related to global ED symptoms, binge eating, and purging. | Group comparison:  ED symptom group (n=268, 25.7%): 39.6% subthreshold insomnia, 30.6% moderate insomnia, 12.7% severe insomnia   Binge eating group (n=265, 25.4%): 43% subthreshold insomnia, 29.4% moderate insomnia, 9.1% severe insomnia  Purging group (n=40, 3.8%): 32.5% subthreshold insomnia, 35% moderate insomnia, 20% severe insomnia  Correlation:  Insomnia+ global ED: r=0.24, p<0.01 Insomnia+ binge eating: r=0.15, p<0.01 Insomnia+ purging: r=0.14, p<0.01  Regression (predicting ED symptoms):  Insomnia: B=0.05, SE=0.01, β=0.22, t=8.12, p<0.001 [0.04; 0.06] |
| Schmidt & Randler (2010) | Composite Scale of Morningness (CSM): 13 items; scores range from 13 (extreme eveningness) to 55 (extreme morningness)   Rising times and bedtimes during weekdays and weekends  Parental monitoring of bedtimes ("My parents set by bedtime"), during week and weekends | Eating Disorder Inventory-2 (EDI-2), German version: 91 questions, capturing 11 dimension; focus on 3 subscales (drive for thinness, bulimia, body dissatisfaction) | Concerning morningness-eveningness, the authors found negative associations between morningness-eveningness and all three scales, thus suggesting that girls scoring higher on eveningness have a more problematic eating behavior. Using the means of all three scales as one measure of eating disorder, we found significant associations between eating disorders and morningness-eveningness, after controlling for age and BMI.   Rising time during the week did not correlate with any measure of eating disorder, while rising time on the weekend and bedtimes during the week and on weekends correlated significantly with drive for thinness and body dissatisfaction. Later bedtimes were correlated with higher scores on body dissatisfaction and drive for thinness, and later rising time on weekends was also correlated with drive for thinness and body dissatisfaction. There were no correlations between rise/bedtimes and bulimic behavior, neither during the week nor on weekends.  There were significant differences between larks and owls in all three measurements. Concerning the three scales, owls had higher scores in drive for thinness, body dissatisfaction, and bulimic behavior | Correlations:  Rise week + drive for thinness: r=-0.01, n.s. Rise week + bulimic: r=0.06, n.s. Rise week + body dissatisfaction: r=-0.07, n.s.  Rise week + mean full scale: r=-0.03, n.s.  Rise weekend + drive for thinness: r=0.22, p<0.001 Rise weekend + bulimic: r=0.04, n.s. Rise weekend + body dissatisfaction: r=0.22, p<0.001 Rise weekend + mean full scale: r=0.22, p<0.001  Bedtime week + drive for thinness: r=0.16, p<0.01 Bedtime week + bulimic: r=0.06, n.s. Bedtime week + body dissatisfaction: r=0.12, p<0.05  Bedtime week + mean full scale: r=0.16, p<0.01  Bedtime weekend + drive for thinness: r=0.22, p<0.001 Bedtime weekend + bulimic: r=0.02, n.s. Bedtime weekend + body dissatisfaction: r=0.16, p<0.01 Bedtime weekend + mean full scale: r=0.19, p<0.01  CSM + drive for thinness: r=-0.12, p<0.05 CSM + bulimic: r=-0.13, p<0.05 CSM + body dissatisfaction: r=-0.24, p<0.001 CSM + mean full scale: r=-0.21, p<0.001  Group comparison:  Mean scale: Evening type: N=38, M=3.06, SD=0.85 Morning type: N=21, M=2.14, SD=0.72 T=4.14, df=57, p<0.001  Drive for thinness: Evening type: N=38, M=3.18, SD=1.29 Morning type: N=21, M=2.24, SD=1.10 T=2.80, df=57, p=0.007  Bulimic behaviour: Evening type: N=38, M=1.95, SD=0.76 Morning type: N=21, M=1.54, SD=0.55 T=2.18, df=57, p=0.03  Body dissatisfaction: Evening type: N=38, M=4.04, SD=1.10 Morning type: N=21, M=2.65, SD=1.01 T=4.77, df=57, p<0.001 |
| Seigel et al. (2004) | Items taken from the Uppsala Sleep Inventory but used measure constructed for this study:  Insomnia score: "How severe are your problems with... difficulties falling asleep after going to bed, frequent awakening during the night, awakening too early in the morning, not feeling sufficiently rested by sleep?" (no, small, moderate, severe, very severe); cut-off= "severe" | Body Image and Eating: "How many times have you attempted to reduce your weight?" (never, 1-5 times, 6-10 times, >10 times); cut-off="6-10 attempts"  "How often do you...fear gaining weight, feel overweight, feel dissatisfied with your body, eat large amounts of food with some loss of self-control, feel impulses to vomit after meals?" (never, seldom, sometimes, fairly often, very often); cut-off="rather often" | Body image problems, frequent attempts to reduce weight, suspected bingeing and impulses to vomit after meals were all significantly associated with difficulties maintaining sleep and with feeling insufficiently rested by sleep.   The combination of suspected bingeing and post-prandial impulses to vomit was associated with difficulties maintaining sleep and, to a lesser degree, early morning awakening, OR 6.9 (2.7-17.3) p<0.0001 and OR 4.6 (1.5-14.4.) p<0.01 respectively.   There were no significant associations between ED symptoms and sleep duration. | Correlations:  Body image & eating composite + insomnia composite: r=0.22, p<0.0001  Group Comparison:  Difficulties Initiating Sleep (DIS):  Recurrent weight-reducing attempts: Yes DIS=19, No DIS=15, OR=1.3 [0.6; 2.8], n.s.  Body dissatisfaction: Yes DIS=34, No DIS=28, OR=1.3 [0.7; 2.4], n.s.  Suspected bingeing: Yes DIS=28, No DIS=16, OR=2.1 [1.1; 4.0], n.s.  Postprandial impulses to vomit: Yes DIS=13, No DIS=6, OR=2.2 [0.9; 5.5], n.s.  Difficulties Maintaining Sleep (DMS):  Recurrent weight-reducing attempts: Yes DMS=33, No DMS=14, OR=3.0 [1.4; 6.3], p<0.01  Body dissatisfaction: Yes DMS=50, No DMS=28, OR=2.6 [1.3; 5.2], p<0.01  Suspected bingeing: Yes DMS=32, No DMS=15, OR=2.6 [1.3; 5.4], p<0.01  Postprandial impulses to vomit: Yes DMS=24, No DMS=5, OR=5.1 [2.2; 12.1], p<0.001  Early Morning Awakening (EMA):  Recurrent weight-reducing attempts: Yes EMA=25, No EMA=15, OR=1.9 [0.7; 4.8], n.s.  Body dissatisfaction: Yes EMA=33, No EMA=28, OR=1.3 [0.5; 3.0], n.s.  Suspected bingeing: Yes EMA=29, No EMA=16, OR=2.2 [0.9; 5.3], n.s.  Postprandial impulses to vomit: Yes EMA=21, No EMA=6, OR=4.0 [1.4; 11.3], p<0.01  Feeling Insufficiently Rested by sleep (FIR):  Recurrent weight-reducing attempts: Yes FIR=25, No FIR=13, OR=2.1 [1.3; 3.4], p<0.01  Body dissatisfaction: Yes FIR=41, No FIR=26, OR=1.9 [1.3; 2.9], p<0.01  Suspected bingeing: Yes FIR=26, No FIR=15, OR=2.0 [1.3; 3.2], p<0.01  Postprandial impulses to vomit: Yes FIR=11, No FIR=6, OR=2.1 [1.1; 4.0], p<0.05 |
| Soares et al. (2011) | Two items: "I have difficulty falling asleep" (DIS) and "I wake up many times during the night" (DMS); rated on 6-point scale (never to always); Sleep disturbance index (SDI) calculated from the sum of DIS and DMS item scores | Eating Attitudes Test-40 (EAT-40): 40 items | Positive significant correlations were observed between sleep difficulties and EAT total scores, and bulimic behaviours for males, females and the total sample. A less consistent pattern was found between sleep difficulties and diet concerns. In the total sample, DIS, DMS and SDI were associated poorly with diet concerns. In females, only DIS and SDI were related poorly with diet concerns. In males, this association was never significant.  Subjects with insomnia symptoms had more disturbed eating behaviour than good sleepers. In the total sample and females, EAT total and diet concerns mean scores and bulimic behaviour were significantly higher in subjects with insomnia symptoms than in good sleepers. Among males, subjects with insomnia symptoms also had significantly higher EAT total scores, and bulimic behaviour than good sleepers, but no significant differences were found regarding diet concerns. | Correlations (total sample):  Diet concerns+DIS: r=0.09, p<0.05 Diet concerns+DMS: r=0.09, p<0.05 Diet concerns+SDI: r=0.10, p<0.01  Bulimic Behaviour+DIS: r=0.19, p<0.001 Bulimic Behaviour+DMS: r=0.17, p<0.001 Bulimic Behaviour+SDI: r=0.21, p<0.001  EAT total+DIS: r=0.15, p<0.001 EAT total+DMS: r=0.14, p<0.001 EAT total+SDI: r=0.18, p<0.001  Group comparison:  Diet Concerns:  Good sleepers: M=18.15, SD=9.64 (N=373) Insomnia symptoms: M=21.24, SD=11.08 (N=173) p=0.002  Bulimic behaviour:  Good sleepers: M=5.18, SD=3.24 Insomnia symptoms: M=7.19, SD=4.28 p=0.001  Total EAT:  Good sleepers: M=47.72, SD=14.48 Insomnia symptoms: M=55.27, SD=17.43 p=0.001 |
| Suna & Ayaz (2022) | Pittsburgh Sleep Quality Index (PSQI), Turkish version: score <=5 considered "good" sleep quality, >5 considered "poor" sleep quality | The Eating Attitude Test (EAT-26), Turkish version: cut-off score=20 Night Eating Questionnaire (NEQ): 14 items | NES was more frequent in students with poor sleep quality than those with good sleep quality (p=0.001). Students with higher PSQI scores had significantly higher total NEQ scores (p=0.001).  The only significant correlation was between sleep disturbances among the PSQI subscales and total EAT-26 score, which indicated greater sleep disturbances with a more significant risk of disordered eating behavior.   Sleep duration, sleep disturbances, sleep latency, daytime functioning, subjective sleep quality, and total PSQI score correlated positively and significantly with NEQ score (p<0.05), suggesting that there is an association between increased total PSQI and the subscale scores mentioned above and risk of the NES.  The multivariate logistic regression analysis revealed that poor sleep quality (PSQI>5) was significantly associated with the NES when adjusted for sociodemographic and lifestyle factors. Further adjustments for depression weakened the association to non-significance.   Poor sleep quality (PSQI > 5) was not associated with disordered eating behavior defined by EAT-26. | Group differences:  Dieting: PSQI >5: n=4 (1.9%), PSQI <=5: n=10 (2.8%), total: n=14 (2.5%), p=0.78  Dieting score (>=18): PSQI >5: M=6.02, SD=4.28 PSQI <=5: M=5.64, SD=4.33,  Total: M=5.78, SD=4.31 p=0.44  Bulimic behaviour: PSQI >5: n=28 (13.6%), PSQI <=5: n=56 (15.5%), total: n=84 (14.8%), p=0.55  Bulimic behaviour score (>=4): PSQI >5: M=1.76, SD=2.65 PSQI <=5: M=1.68, SD=2.31,  Total: M=1.71, SD=2.44 p=0.78  Oral control behaviour: PSQI >5: n=78 (37.9%), PSQI <=5: n=139 (38.4%), total: n=217 (38.2%), p=0.90  Oral control score (>=5): PSQI >5: M=4.21, SD=3.83 PSQI <=5: M=4.18, SD=3.65,  Total: M=4.19, SD=3.71 p=0.86  Total EAT-26 score: PSQI >5: M=12.26, SD=7.21 PSQI <=5: M=11.72, SD=7.34,  Total: M=11.9, SD=7.29 p=0.60  Abnormal EB: PSQI >5: n=27 (13.1%), PSQI <=5: n=42 (11.6%), total: n=69 (12.1%) Normal EB: PSQI >5: n=179 (86.9%), PSQI <=5: n=320 (88.4%), total: n=499 (87.9%) p=0.60  Total NEQ score: PSQI >5: M=15.43, SD=5.30 PSQI <=5: M=12.58, SD=4.82,  Total: M=13.61, SD=5.18 p=0.001  NES: PSQI >5: n=14 (6.8%), PSQI <=5: n=10 (2.8%), total: n=24 (4.2%) Non-NES: PSQI >5: n=192 (93.2%), PSQI <=5: n=352 (97.2%), total: n=544 (95.8%) p=0.02  Correlations:  Sleep duration + EAT-26: r=-0.001, n.s. Sleep duration + NEQ: r=0.10, p<0.05  Sleep disturbance + EAT-26: r=0.11, p<0.05 Sleep disturbance + NEQ: r=0.14, p<0.001  Sleep latency + EAT-26: r=-0.006, n.s. Sleep latency + NEQ: r=0.29, p<0.001  Daytime functioning + EAT-26: r=0.08, n.s. Daytime functioning + NEQ: r=0.15, p<0.001  Habitual sleep efficiency + EAT-26: r=0.01, n.s. Habitual sleep efficiency + NEQ: r=0.07, n.s.  Subjective sleep quality + EAT-26: r=0.03, n.s. Subjective sleep quality + NEQ: r=1.67, p<0.001  Sleep medication + EAT-26: r=-0.25, n.s. Sleep medication + NEQ: r=0.07, n.s.  PSQI + EAT-26: r=0.06, n.s. PSQI + NEQ: r=0.30, p<0.001  Logistic regression (poor sleep quality PSQI>5):  Abnormal eating behaviour (n=69):  Model 1 (age, sex): OR=1.20 [0.71; 2.02], p=0.50 Model 2 (age, sex, class standing, residency, smoking, alcohol): OR=1.11 [0.65; 1.89], p=0.70 Model 3 (age, sex, class standing, residency, smoking, alcohol, depression): OR=0.86 [0.49; 1.51], p=0.60  Night Eating Syndrome (n=24):  Model 1 (age, sex): OR=2.83 [1.22; 6.54], p=0.02 Model 2 (age, sex, class standing, residency, smoking, alcohol): OR=2.54 [1.02; 6.34], p=0.046 Model 3 (age, sex, class standing, residency, smoking, alcohol, depression): OR=1.92 [0.74; 5.02], p=0.18 |
| Tăut et al. (2018) | 1 item on difficulties in getting to sleep (part of health complaints): about every day, more than once a week, almost every week, almost every month, rarely or never (absent=rarely/never, present=everything else) | Body weight dissatisfaction: "Do you think your body is..." (much too thin, too thin, about the right size, too fat, much too fat)  Unhealthy weight control: "Which of the following things have you done in the last 12 months in order to lose weight" (skipping meals, smoking, vomiting, using pills, restricting their diet to one of more foods); answered as yes/no | While BMI was not significant as a predictor, body weight dissatisfaction and the interaction between BMI x body weight dissatisfaction, respectively, were significant in accounting for the variance in self-reported sleep difficulties. Again, adolescents with lower BMIs and who considered themselves too fat had a higher risk of experiencing sleeping difficulties compared to their counterparts with higher BMI. | Regression (predicting sleep difficulties):  Body weight dissatisfaction: B=-0.63, p=0.02, OR=0.52  BMI x body weight dissatisfaction: B=0.03, p=0.03, OR=1.03 |
| Tholin et al. (2009) | Sleep-related problems: "Have you had difficulty falling asleep (during the past 3 months)?", "Have you had feelings of not having had enough sleep on awakening (during the last 6 months)?", "Have you had disturbed or restless sleep (during the last 6 months)?"; sleep problem = "usually"/"always" | Night eating (broad): awakening with food intake during the night at least once a week and/or 25% of daily food intake after the evening meal  Night eating (narrow): awakening with food intake at least once a week and/or <=50% of daily food intake after the evening meal  Night eating: "How often do you get up at night to eat?" (never, once or twice, weekly, nightly, don't know/wish not to answer) and "What proportion of your daily food intake takes place after the evening meal?" (0, 1-24, 25-49, 50-74, 75-100, don't know/wish not to answer)  Binge eating: "Have you ever had binges when you ate what most people would regard as an unusually large amount of food in a short period of time?" (yes, no, don't know/refuse) and "When you were having eating binges, did you feel that your eating was out of control?" (not at all, slightly, moderately, very much, extremely, don't know/don't wish to answer); binge eaters = "yes" and "very much"/"extremely" | Amongst responders, men and women with NE had higher frequencies of the three different aspects of sleep-related problems than those without NE. The risk of having difficulty falling asleep was 3–4 times higher in men and women with broad and narrow NE compared with individuals without NE. Risks were also elevated (about 1.5–3 times) for feeling that they needed more sleep on awakening and for disturbed or restless sleep (about 2.5–3.5 times) | Group comparison:  Men (N=2588):  Difficulties falling asleep: n=206: No NE (broad): 7.3 Yes NE (broad): 21.6 OR=3.41 [2.41; 5.41] No NE (narrow): 7.5 Yes NE (narrow): 26.2 OR=3.75 [2.04; 6.87]  Not enough sleep on awakening: n=589: No NE (broad): 22.3 Yes NE (broad): 31.2 OR=1.56 [1.05; 2.32] No NE (narrow): 22.5 Yes NE (narrow): 33.8 OR=1.67 [1.01; 2.77]  Disturbed or restless sleep: n=234: No NE (broad): 8.5 Yes NE (broad): 20.0 OR=2.76 [1.72; 4.44] No NE (narrow): 8.6 Yes NE (narrow): 23.0 OR= 3.41 [1.95; 5.96]  Women (n=3620):  Difficulties falling asleep: n=425: No NE (broad): 11.1 Yes NE (broad): 29.6 OR=3.29 [2.21; 4.91] No NE (narrow): 11.3 Yes NE (narrow): 31.6 OR=3.42 [2.06; 5.66]  Not enough sleep on awakening: n=1046: No NE (broad): 28.2 Yes NE (broad): 49.8 OR=2.51 [1.74; 3.62] No NE (narrow): 28.4 Yes NE (narrow): 54.5 OR=3.14 [1.97; 5.01]  Disturbed or restless sleep: n=603: No NE (broad): 16.2 Yes NE (broad): 33.7 OR=2.65 [1.80; 3.89] No NE (narrow): 16.3 Yes NE (narrow): 38.6 OR=3.19 [1.97; 5.16] |
| Trace et al. (2012) | Items on sleep habits and problems: "Do you usually take a nap at least every second day?" (yes, no), "Do you think you get enough sleep?" (yes, definitely enough, yes, mostly enough, no, a bit too little, no, clearly not enough, no, far from enough), "How do you think you sleep on the whole?" (very well, pretty well, neither poorly nor well, pretty poorly, very poorly), "Try to determine to what degree you are a morning person or a night person" (definitely a morning person, to some degree a morning person, to some degree a night person, definitely a night person); complaints during past 3 months: "Problem falling asleep?", "Sleepy during work or free time?"; complaints during past 6 months: "Waking up too early and not being able to sleep again?", "Feeling of not having enough sleep on awakening?", "Disturbed or uneasy sleep", both assessed with 5 categories (never, seldom, sometimes, usually, always) | Lifetime history of binge eating (based on SCID), 2 items: "Have you ever had eating binges when you ate what most people would regard as an unusually large amount of food in a short period of time?" (yes, no, do not know/refuse) and "When you were having eating binges, did you feel your eating was out of control?" (not at all, slightly, somewhat, very much, extremely, do not know/refuse); BE group defined as yes (first item) and slightly/somewhat/very much/extremely (second item) | Women who reported not getting enough sleep, sleeping poorly, problems falling asleep, feeling sleepy during work or free time, and disturbed sleep were significantly more likely to report life-time BE after accounting for age at interview, current cohabiting status, and lifetime depression diagnosis.  Women who reported waking too early and those reporting not getting enough rest were more likely to report BE; however, when these models were adjusted for lifetime depression diagnosis, the associations were no longer significant.  Adjusting for obesity status in the models did not change the findings: associations between self-reported sleep problems and lifetime BE remained significant. | Logistic regression predicting lifetime binge eating:  Analysis 1 (adjusted for age, cohabiting status):  Napping (at least every 2nd day): OR=1.01 [0.72; 1.40], X2=0.00, p=0.97  Sleeping mostly enough: OR=0.79 [0.51; 1.24] Sleeping a bit too little: OR=1.21 [0.77; 1.91] Sleeping clearly not enough: OR=1.58 [0.89; 2.79] Sleeping far from enough: OR=3.22 [1.69; 6.11] X2=19.39, p=0.001  Sleeping pretty well: OR=1.92 [1.25; 2.95] Sleeping neither poorly nor well: OR=2.64 [1.62; 4.33] Sleeping pretty poorly: OR=3.69 [2.25; 6.03] Sleeping very poorly: OR=9.01 [4.87; 16.67] X2=44.0, p=0.001  Seldom problems falling asleep: OR=1.34 [0.84; 2.14] Sometimes problems falling asleep: OR=2.23 [1.43; 3.49] Usually problems falling asleep: OR=3.82 [2.31; 6.34] Always problems falling asleep: OR=7.15 [3.84; 13.30] X2=42.85, p=0.001  Seldom sleepy during work or free time: OR=0.69 [0.31; 1.54] Sometimes sleepy: OR=1.07 [0.50; 2.29] Usually sleepy: OR=2.05 [0.94; 4.52] Always sleepy: OR=3.91 [1.67; 9.11] X2=36.54, p=0.001  Seldom waking too early: OR=1.58 [1.08; 2.30] Sometimes waking too early: OR=2.01 [1.37; 2.94] Usually waking too early: OR=2.30 [1.33; 3.97] Always waking too early: OR=4.43 [1.95; 10.08] X2=20.17, p=0.001  Seldom not enough rest: OR=1.11 [0.47; 2.60] Sometimes not enough rest: OR=1.41 [0.62; 3.22] Usually not enough rest: OR=1.67 [0.71; 3.89] Always not enough rest: OR=4.14 [1.71; 10.07] X2=17.82, p=0.002  Seldom disturbed sleep: OR=1.68 [0.90; 3.15] Sometimes disturbed sleep: OR=3.45 [1.90; 6.27] Usually disturbed sleep: OR=3.95 [2.06; 7.55] Always disturbed sleep: OR=7.72 [3.70; 16.09] X2=50.77, p=0.001 |
| Uyar et al. (2023) | Morning-Evening Scale (MEQ): 19 items, higher scores reflect more morning preference, scores 16-41 "evening type", 42-58 "intermediate type", 59-86 "morning type" | Night Eating Questionnaire (NEQ): 14 items, cut-off score >=25 and >=30 (increased specificity) | The night-eating syndrome score was higher in evening-type individuals than intermediate- and morning-types. | Group comparison:  Morning: n=64 Intermediate: n=239 Evening: n=59  NEQ (score):  Morning: 14.5 +/- 4.02 Intermediate: 16.1 +/- 5.41 Evening: 17.6 +/- 5.17 p=0.003 Post-hoc test: E>M, E>I, M=I  Yes NEQ (%):  Morning: - Intermediate: n=6 (6.3%) Evening: n=1 (1.7%) p=NA  No NEQ (%):  Morning: n=64 (100%) Intermediate: n=233 (97.5%) Evening: n=58 (98.3%) NA |
| Vrabec et al. (2022) | Social jetlag: bedtimes and waketimes on both weekends and weekdays (mid-sleep time was recorded by calculating the time at the midpoint of the sleeping period (MST); difference between MST on weekdays and weekends was calculated)  Morningness-Eveningsness Questionnaire (MEQ): 19 items, evening type = 41 or below, intermediate = 42-58, morning = 59 or above  PROMIS Pediatric Sleep-Related Impairment Short Form 8a: 8 items   Sleep quantity: calculated from self-report bedtime and waketimes on weekends and weekdays | Loss of Control over Eating Scale (LOCES): 7 items | The loss of control over eating analysis was marginally significant. | Regression with social jetlag predicting eating styles, when controlling for age, sex, chronotype:  Chronotype -> loss of control eating: β=-0.03, t=-0.51, p=0.61  Social jetlag -> loss of control eating: β=0.10, t=1.97, p=0.05, F(4, 367)=2.41, p=0.049  Regression with sleep quality predicting eating style when controlling for age and sex:  Sleep quality -> loss of control eating: β=0.30, t=6.07, p<0.001, F(3, 368)=14.15, p<0.001  Sleep quantity (weekday) -> loss of control eating: β=-0.12, t=-2.02, p=0.04  Sleep quantity (weekend) -> loss of control eating: β=-0.02, t=-0.35, p=0.09, F(4, 367)=2.83, p=0.03 |
| Walker et al. (2018) | Sleep was measured via two self-report items asking about satisfaction with current sleep pattern (i.e., sleep quality) and number of minutes it typically took to fall asleep over the past month (i.e., sleep onset latency, SOL). | Restrictive eating was measured using an item from a national youth risk survey that asked about restriction of food, calories, or fat content in food intake with the intent of weight loss over the past 30 days. | The overall models were significant for both sleep quality and sleep onset latency. Predictors accounted for 17% of the variability of sleep quality and 9% of the variability of sleep onset latency. When trait anxiety and restrictive eating were examined as predictors of sleep quality and SOL, only trait anxiety significantly predicted poorer sleep quality. | Model including gender BMI, trait anxiety and restrictive eating, sleep quality and sleep onset latency:  n.s. effect for restrictive eating |
| White et al. (2024) | Time-in-bed: typical weekday bedtimes and wake times (average school night)  Paediatric Insomnia Severity Index (PISI): 6 items, reference to past week | Body Dissatisfaction Scale (BDS): 9 computer-generated female and male body images, body dissatisfaction rated as difference between ideal and actual body ratings | Less time-in-bed was related to greater body dissatisfaction | Correlations:  PISI+BDS: r=-0.01, n.s. Time-in-bed + BDS: r=-0.23, p<0.05 |
| Wroblevski et al. (2022) | The variable "insomnia" has a value of 1 if the student answered that they always or almost always have trouble sleeping because something worries them and zero if they never, rarely or sometimes have trouble sleeping. | Body dissatisfaction: "How do you feel about your body?". The answers are: very satisfied, satisfied, indifferent, dissatisfied and very dissatisfied. The “treatment” group included students who answered that they were dissatisfied or very dissatisfied with their body.  The “treatment” group is defined by students who reported some level of dissatisfaction with their own body image and the control group is made up of students who did not report being dissatisfied with their body image. | Results indicated that body dissatisfaction is associated with higher levels of insomnia among the interviewed adolescents. Schoolchildren who reported being dissatisfied with their body image were more likely to not being able to sleep. | Average Treatment Effect of the Treated (ATT) values by gender for Insomnia as an outcome and body dissatisfaction as a predictor:  Boys:  ATT=0.06  DP=0.005, p<0.01  Girls:  ATT=0.07  DP=0.005, p<0.01 |
| Wu et al. (2021) | Pittsburgh Sleep Quality Index (PSQI) Chinese version | Eating Attitude Test-26 (EAT-26): 26 items | Sleep quality was positively associated with disordered eating behaviours.   The association between poor sleep quality and DEBs was statistically significant and a total of 15.7% of the variance was explained by the combined contribution of sleep quality and covariates. | Correlations:  Sleep quality + disordered eating: r=0.19, p<0.001 |
| Yeh & Brown (2014) | Pittsburgh Sleep Quality Index (PSQI): 19 items; score >5 indicates moderate to severe sleep difficulties (differentiates poor and good sleepers) | Binge Eating Scale (BES): 16 items, score>=27 considered to be binge eaters  Night Eating Questionnaire (NEQ): 14 items, score =25 identifies possible NES cases | Worse sleep quality was associated with night-time eating, but not binge-eating. | Correlations:  Sleep duration + BES: r=0.20, p<0.01 Sleep disturbances + BES: r=0.22, p<0.01 Sleep latency + BES: r=0.31, n.s. Daytime dysfunction + BES: r=0.39, p<0.01 Habitual sleep efficiency + BES: r=0.19, p<0.01 Subjective sleep quality + BES: r=0.31, p<0.01 Use of sleep medication + BES: r=0.22, p<0.01 PSQI total + BES: r=0.41, p<0.01  Sleep duration + NEQ: r=0.33, p<0.01 Sleep disturbances + NEQ: r=0.40, p<0.01 Sleep latency + NEQ: r=0.42, p<0.05 Daytime dysfunction + NEQ: r=0.41, p<0.01 Habitual sleep efficiency + NEQ: r=0.33, p<0.01 Subjective sleep quality + NEQ: r=0.47, p<0.01 Use of sleep medication + NEQ: r=0.25, p<0.01 PSQI total + NEQ: r=0.58, p<0.01  Hierarchical regression (predicting total sleep quality, including age, relationship status, gender, depression, BMI, night-eating and binge eating):  Night-eating: B=0.22, beta=0.40, SE=0.03, t=7.47, p<0.005  Binge eating: B=-0.02, beta=-0.05, SE=0.02, t=-0.90, n.s. |
| Yilmaz Yavuz & Altinsoy (2022) | Morningness-Eveningness Questionnaire (MEQ): 19 items, divided into morning type (score: 59-86), intermediate type (42-58) and evening type (16-41) | Night Eating Questionnaire (NEQ): sore >= 25 considered NES | As the scores of the academics' Morningness-Eveningness Questionnaire decreased (predisposition for eveningness), the scores of the Night Eating Questionnaire (which yielded a value compatible with NES) increased.   A NES compatible score showed a statistically significant difference according to the chronotype of academics. The NES compatible score was 29.2% higher in the evening type than in the other chronotypes.   The MEQ scores were significant predictors of NEQ scores. | Correlation:  MEQ + NEQ: r=-0.29, p<0.001  Group comparison:  Evening type: n=17 (70.8%) no night eating, n=7 (29.2%) night eating Intermediate type: n=139 (92.7%) no night eating, n=11 (7.3%) night eating Morning type: n=45 (95.7%) no night eating, n=2 (4.3%) night eating  X2=13.65, p=0.001  Linear regression (predicting NEQ, including predictors fear of COVID-19, MEQ)  MEQ -> NEQ:  B=-0.19, beta=-0.23, SE=0.05, t=-3.66, p<0.001 |
