# Supplementary material for: The Association Between Disordered Eating and Sleep in Non‐Clinical Populations—A Systematic Review and Meta‐Analysis
Source: J Sleep Res. 2025 Jun 30;35(2):e70117. doi: 10.1111/jsr.70117 (PMC13003291; doi:10.1111/jsr.70117)
Supplement: Supplementary file 2 — TABLE S2: Overview of quality assessments. [file JSR-35-e70117-s001.docx]

Supplementary Table 2. Overview of Quality Assessments

| Author(s) | Was the research question or objective in this paper clearly stated? | Was the study population clearly specified and defined? | Was the participation rate of eligible persons at least 50%? | Were all the subjects selected or recruited from the same or similar populations (including the same time period)? | Were inclusion and exclusion criteria for being in the study prespecified and applied uniformly to all participants? | Was a sample size justification, power description, or variance and effect estimates provided? | For the analyses in this paper, were the exposure(s) of interest measured prior to the outcome(s) being measured? | Was the timeframe sufficient so that one could reasonably expect to see an association between exposure and outcome if it existed? | For exposures that can vary in amount or level, did the study examine different levels of the exposure as related to the outcome (e.g., categories of exposure, or exposure measured as continuous variable)? | Were the exposure measures (independent variables) clearly defined, valid, reliable, and implemented consistently across all study participants? | Was the exposure(s) assessed more than once over time? | Were the outcome measures (dependent variables) clearly defined, valid, reliable, and implemented consistently across all study participants? | Were the outcome assessors blinded to the exposure status of participants? | Was loss to follow-up after baseline 20% or less? | Were key potential confounding variables measured and adjusted statistically for their impact on the relationship between exposure(s) and outcome(s)? |
| --- | --- | --- | --- | --- | --- | --- | --- | --- | --- | --- | --- | --- | --- | --- | --- |
| Ahorsu et al. (2023) | Yes | No | No | No | Yes | Yes | No | No | Yes (categories, continuous) | Clearly defined; Valid; Reliable; Implemented consistently | No | Clearly defined; Valid; Reliable; Implemented consistently | No | NA | Yes |
| Akbari et al. (2022) | Yes | No | No | Yes | No | No | No | Other: No | Yes (categories, continuous) | Clearly defined; Valid; Reliable; Implemented consistently | No | Clearly defined; Valid; Reliable; Implemented consistently | No | NA | Yes |
| Akram et al. (2021) | Yes | No | Not to be deter-mined | Not to be deter-mined | Not to be deter-mined | Yes | No | No | Yes (categories, continuous) | Clearly defined; Valid; Reliable; Implemented consistently | No | Clearly defined; Valid; Reliable; Implemented consistently | No | NA | Yes |
| al Balushi & Carciofo (2023) | Yes | No | No | No | No | Yes | No | No | Yes (categories, continuous) | Clearly defined; Valid; Reliable; Implemented consistently | No | Clearly defined; Valid; Reliable; Implemented consistently | No | NA | Yes |
| Aleksic et al. (2023) | Yes | Yes | No | Yes | Not to be deter-mined | Yes | No | No | No | Clearly defined; Valid; Reliable; Implemented consistently | No | Implemented consistently | No | NA | No |
| Aloi et al. (2017) | Yes | Yes | No | Yes | Yes | No | No | No | Yes (categories, continuous) | Clearly defined; Valid; Reliable; Implemented consistently | No | Clearly defined; Valid; Reliable; Implemented consistently | No | NA | No |
| Altan et al. (2018) | Yes | Yes | No | Yes | Yes | Yes | No | No | Not to be determined | Implemented consistently | No | Clearly defined; Valid; Reliable; Implemented consistently | No | NA | No |
| Arslan & Aydemir (2019) | Yes | No | No | Yes | Yes | No | No | No | No | Clearly defined; Valid; Reliable; Implemented consistently | No | Clearly defined; Valid; Reliable; Implemented consistently | No | NA | No |
| Aspen et al. (2014) | Yes | No | No | Yes | Yes | No | No | No | Yes (categories, continuous) | Clearly defined; Valid; Reliable; Implemented consistently | No | Clearly defined; Valid; Reliable; Implemented consistently | No | NA | Yes |
| Babayan et al. (2018) | Yes | No | No | Yes | Yes | Yes | No | No | Yes (categories, continuous) | Clearly defined; Reliable; Implemented consistently | No | Not to be determined | No | NA | Not to be deter-mined |
| Bahri et al. (2015) | Yes | No | No | Yes | Yes | No | No | No | Yes (categories, continuous) | Clearly defined; Implemented consistently | No | Clearly defined; Valid; Reliable; Implemented consistently | No | NA | Not to be determined |
| Barnes et al. (2023) | Yes | No | No | Yes | No | Yes | No | No | Yes (categories, continuous) | Clearly defined; Valid; Reliable; Implemented consistently | No | Clearly defined; Valid; Reliable; Implemented consistently | No | NA | Yes |
| Bener et al. (2006) | Yes | Yes | No | Yes | Yes | Yes | No | No | Yes (categories, continuous) | Implemented consistently | No | Implemented consistently | No | NA | Not to be deter-mined |
| Berntzen et al. (2021) | Yes | Yes | No | Not to be deter-mined | Yes | Yes | No | No | Yes (categories, continuous) | Clearly defined; Valid; Reliable; Implemented consistently | No | Clearly defined; Valid; Reliable; Implemented consistently | No | NA | Yes |
| Birkeland et al. (2012) | Yes | Yes | No | Yes | Yes | No | Yes | Yes | Yes (categories, continuous) | Clearly defined; Valid; Reliable; Implemented consistently | No | Clearly defined; Valid; Reliable; Implemented consistently | Yes | No | Yes |
| Blouchou et al. (2024) | Yes | Yes | No | Not to be deter-mined | Yes | No | No | No | No | Clearly defined; Implemented consistently | No | Clearly defined; Implemented consistently | No | NA | Yes |
| Borisenkov et al. (2020) | Yes | Yes | No | No | Yes | No | No | No | Yes (categories, continuous) | Clearly defined; Valid; Reliable; Implemented consistently | No | Clearly defined; Valid; Reliable; Implemented consistently | No | NA | Yes |
| Bos et al. (2013) | Yes | Yes | No | Yes | Yes | No | Yes | Yes | No | Clearly defined; Valid; Reliable; Implemented consistently | Not to be deter-mined | Clearly defined; Implemented consistently | No | No | Yes |
| Bruck & Astbury (2012) | Yes | Yes | No | Yes | Yes | No | No | No | No | Clearly defined; Implemented consistently | No | Clearly defined; Implemented consistently | No | NA | Yes |
| Cakir et al. (2018) | Yes | Not to be deter-mined | Not to be deter-mined | Yes | Not to be deter-mined | No | No | No | No | Implemented consistently | No | Implemented consistently | No | NA | Not to be deter-mined |
| Cecen & Guleken (2023) | Yes | No | No | No | Yes | No | No | No | Yes (categories, continuous) | Clearly defined; Valid; Reliable; Implemented consistently | No | Clearly defined; Valid; Reliable; Implemented consistently | No | NA | Not to be deter-mined |
| Ceylan et al. (2024) | Yes | No | Not to be deter-mined | Not to be deter-mined | Yes | Yes | No | No | Yes (categories, continuous) | Clearly defined; Valid; Reliable; Implemented consistently | No | Clearly defined; Valid; Reliable; Implemented consistently | No | NA | Yes |
| Chardon et al. (2016) | Yes | No | No | Yes | Yes | No | No | No | Yes (categories, continuous) | Clearly defined; Valid; Reliable; Implemented consistently | No | Clearly defined; Valid; Reliable; Implemented consistently | No | NA | Yes |
| Clark (2018) | Yes | No | No | Yes | Yes | No | No | No | Yes (categories, continuous) | Clearly defined; Valid; Reliable; Implemented consistently | No | Reliable; Implemented consistently | No | NA | No |
| Cooper (2022) | Yes | Yes | No | Yes | Yes | No | Yes | Yes | No | Clearly defined; Implemented consistently | Yes | Clearly defined; Implemented consistently | No | Yes | Yes |
| De Young et al. (2022) | Yes | No | No | Yes | Yes | No | No | No | Yes (categories, continuous) | Clearly defined; Valid; Reliable; Implemented consistently | Yes | Clearly defined; Valid; Reliable; Implemented consistently | No | NA | Not to be deter-mined |
| EE & Gan, 2022 | Yes | No | No | Not to be deter-mined | Yes | No | No | No | No | Clearly defined; Valid; Reliable; Implemented consistently | No | Clearly defined; Valid; Reliable; Implemented consistently | No | NA | Not to be deter-mined |
| Eid et al. (2022) | Yes | No | No | Yes | Yes | Yes | Yes | Yes | Yes (categories, continuous) | Clearly defined; Valid; Reliable; Implemented consistently | Yes | Clearly defined; Valid; Reliable; Implemented consistently | No | Yes | Yes |
| Farhangi (2019) | Yes | No | No | Yes | No | Yes | No | No | Yes (categories, continuous) | Clearly defined; Valid; Reliable; Implemented consistently | No | Clearly defined; Valid; Reliable; Implemented consistently | No | NA | No |
| Fernández-Argüelles et al. (2022) | Yes | No | No | Yes | Yes | No | No | No | Yes (categories, continuous) | Clearly defined; Valid; Reliable; Implemented consistently | No | Clearly defined; Reliable; Implemented consistently | No | NA | Yes |
| Figueroa et al. (2024) | Yes | Yes | No | Yes | Yes | No | No | No | Yes (categories, continuous) | Clearly defined; Valid; Reliable; Implemented consistently | No | Clearly defined; Valid; Reliable; Implemented consistently | No | NA | Yes |
| Fords-ham et al. (2019) | Yes | No | No | Not to be deter-mined | No | No | No | No | Yes | Yes | No | Clearly defined, valid, reliable, implemented consistently | No | NA | No |
| Gallant et al. (2013) | Yes | No | No | Yes | Yes | No | No | No | No | Clearly defined; Implemented consistently | No | Clearly defined; Implemented consistently | No | NA | Not to be deter-mined |
| Gundogdu & Yildirim (2023) | Yes | Yes | No | Yes | Yes | No | No | No | Yes (categories, continuous) | Clearly defined; Valid; Reliable; Implemented consistently | No | Clearly defined; Valid; Reliable; Implemented consistently | No | NA | No |
| Hafstad et al. (2013) | Yes | Yes | Yes | Yes | Not to be deter-mined | Not to be deter-mined | Yes | Yes | Yes (categories, continuous) | Clearly defined; Valid; Reliable; Implemented consistently | Yes | Clearly defined; Valid; Reliable; Implemented consistently | No | No | Yes |
| Hao et al. (2023) | Yes | No | No | Yes | No | No | No | No | Yes (categories, continuous) | Clearly defined; Valid; Reliable; Implemented consistently | No | Clearly defined; Valid; Reliable; Implemented consistently | No | NA | Yes |
| Hasan et al. (2023) | Yes | Yes | No | Yes | Yes | Yes | No | No | No | Clearly defined; Valid; Reliable; Implemented consistently | No | Clearly defined; Valid; Reliable; Implemented consistently | No | NA | Yes |
| Hirai et al. (2022) | Yes | Yes | Not to be deter-mined | Yes | Yes | Yes | No | No | No | Clearly defined; Valid; Reliable; Implemented consistently | No | Clearly defined; Valid; Reliable; Implemented consistently | No | NA | No |
| Johnson (2020) | Yes | No | No | Not to be deter-mined | Yes | No | Yes | Yes | Yes (categories, continuous) | Clearly defined; Valid; Reliable; Implemented consistently | No | Clearly defined; Valid; Reliable; Implemented consistently | No | Yes | No |
| Kandeger et al. (2018) | Yes | No | No | Not to be deter-mined | Yes | No | No | No | Yes (categories, continuous) | Clearly defined; Valid; Reliable; Implemented consistently | No | Clearly defined; Valid; Reliable; Implemented consistently | No | NA | Yes |
| Kandeger et al. (2019) | Yes | No | No | Not to be deter-mined | Yes | Yes | No | No | Yes (categories, continuous) | Clearly defined; Valid; Reliable; Implemented consistently | No | Clearly defined; Valid; Reliable; Implemented consistently | No | NA | Yes |
| Kiltie et al. (2024) | Yes | Yes | No | Yes | No | Yes | No | No | Yes (categories, continuous) | Clearly defined; Implemented consistently | No | Clearly defined; Valid; Reliable; Implemented consistently | No | NA | Not to be deter-mined |
| Krawitz (2011) | Yes | Yes | No | Yes | Yes | No | Yes | Yes | Yes (categories, continuous) | Clearly defined; Valid; Reliable; Implemented consistently | Yes | Clearly defined; Valid; Reliable; Implemented consistently | No | No | Not to be deter-mined |
| Lauer et al. (2021) | Yes | No | No | Not to be deter-mined | Yes | No | No | No | Yes (categories, continuous) | Clearly defined; Implemented consistently | No | Clearly defined; Valid; Reliable; Implemented consistently | No | NA | No |
| Lee & Suh (2018) | Yes | No | No | Not to be deter-mined | Yes | No | No | No | Yes (categories, continuous) | Clearly defined; Valid; Reliable; Implemented consistently | No | Clearly defined; Valid; Reliable; Implemented consistently | No | NA | Yes |
| Lee et al. (2021) | Yes | No | No | Not to be deter-mined | Yes | No | No | No | Yes (categories, continuous) | Clearly defined; Valid; Reliable; Implemented consistently | No | Clearly defined; Reliable; Implemented consistently | No | NA | No |
| Lew et al. (2020) | Yes | Yes | Yes | Yes | Yes | No | No | No | No | Clearly defined; Implemented consistently | No | Clearly defined; Implemented consistently | No | NA | Yes |
| Lin et al. (2020) | Yes | No | No | Yes | Yes | No | No | No | Yes (categories, continuous) | Clearly defined; Valid; Reliable; Implemented consistently | No | Clearly defined; Valid; Reliable; Implemented consistently | No | NA | Yes |
| Liu et al. (2022) | Yes | Yes | Not to be deter-mined | Yes | Yes | Yes | No | No | Yes (categories, continuous) | Clearly defined; Valid; Reliable; Implemented consistently | No | Clearly defined; Valid; Reliable; Implemented consistently | No | NA | No |
| Lo et al. (2011) | Yes | Yes | Not to be deter-mined | Yes | Yes | No | No | No | Yes (categories, continuous) | Clearly defined; Implemented consistently | No | Clearly defined; Implemented consistently | No | NA | Yes |
| Lombardo et al. (2010) | Yes | Not to be deter-mined | No | Not to be deter-mined | Not reported | No | No | No | Yes (categories, continuous) | Clearly defined; Valid; Reliable; Implemented consistently | No | Clearly defined; Valid; Reliable; Implemented consistently | No | NA | Not to be deter-mined |
| Lombardo et al. (2013) | Yes | No | No | Not to be deter-mined | Yes | Yes | No | No | Yes (categories, continuous) | Clearly defined; Valid; Reliable; Implemented consistently | No | Clearly defined; Valid; Reliable; Implemented consistently | No | NA | No |
| Lombardo et al. (2014) | Yes | No | No | Not to be deter-mined | Yes | No | No | No | Yes (categories, continuous) | Clearly defined; Valid; Reliable; Implemented consistently | No | Clearly defined; Valid; Reliable; Implemented consistently | No | NA | Yes |
| Lundgren et al. (2008) | Yes | No | No | Not to be deter-mined | Yes | No | No | No | No | Clearly defined; Valid; Reliable; Implemented consistently | No | Clearly defined; Valid; Reliable; Implemented consistently | No | NA | No |
| Manasse et al. (2022) | Yes | No | No | Not to be deter-mined | Yes | No | Yes | Yes | Yes (categories, continuous) | Clearly defined; Valid; Reliable; Implemented consistently | Yes | Clearly defined; Valid; Reliable; Implemented consistently | No | No | Yes |
| Mason & Heron (2016) | Yes | No | No | Yes | Not reported | No | Yes | Yes | Yes (categories, continuous) | Clearly defined; Implemented consistently | No | Clearly defined; Implemented consistently | No | Not to be deter-mined | Yes |
| Matias et al. (2020) | Yes | Yes | Yes | Yes | Yes | Yes | No | No | Yes (categories, continuous) | Clearly defined; Implemented consistently | No | Clearly defined; Implemented consistently | No | NA | Yes |
| Meule et al. (2014) | Yes | No | No | Not to be deter-mined | Yes | No | No | No | Yes (categories, continuous) | Clearly defined; Valid; Reliable; Implemented consistently | No | Clearly defined; Valid; Reliable; Implemented consistently | No | NA | Yes |
| Mori et al. (2009) | Yes | Yes | Not to be deter-mined | Yes | Yes | No | No | No | Yes (categories, continuous) | Clearly defined; Implemented consistently | No | Clearly defined; Implemented consistently | No | NA | Yes |
| Nagata et al. (2021) | Yes | Yes | Not to be deter-mined | Yes | Yes | No | Yes | Yes | Yes (categories, continuous) | Clearly defined; Implemented consistently | No | Clearly defined; Implemented consistently | No | Yes | Yes |
| Natale et al. (2008) | Yes | No | No | Not to be deter-mined | Yes | No | No | No | Yes (categories, continuous) | Clearly defined; Valid; Reliable; Implemented consistently | No | Clearly defined; Valid; Reliable; Implemented consistently | No | NA | No |
| Nolan & Geliebter (2016) | Yes | No | No | No | No | No | No | No | Yes (categories, continuous) | Clearly defined; Valid; Reliable; Implemented consistently | No | Clearly defined; Valid; Reliable; Implemented consistently | No | NA | Yes |
| Nolan & Geliebter (2019) | Yes | No | No | No | No | No | No | No | Yes (categories, continuous) | Clearly defined; Valid; Reliable; Implemented consistently | No | Clearly defined; Valid; Reliable; Implemented consistently | No | NA | No |
| Park et al. (2020) | Yes | Yes | No | Yes | Yes | No | No | No | Yes (categories, continuous) | Clearly defined; Implemented consistently | No | Clearly defined; Implemented consistently | No | NA | No |
| Parker et al. (2022 & 2020) | Yes | Yes | No | Not to be deter-mined | Yes | Yes | Yes | Yes | Yes (categories, continuous) | Clearly defined; Valid; Reliable; Implemented consistently | Yes | Clearly defined; Implemented consistently | No | No | Yes |
| Prieto et al. (2012) | Yes | No | No | Not to be deter-mined | Yes | No | No | No | Yes (categories, continuous) | Clearly defined; Valid; Reliable; Implemented consistently | No | Clearly defined; Implemented consistently | No | NA | Yes |
| Ramos et al. (2023) | Yes | Yes | No | Yes | Yes | Yes | No | No | Yes (categories, continuous) | Clearly defined; Implemented consistently | No | Clearly defined; Implemented consistently | No | NA | Yes |
| Reche-Garcia et al. (2018) | Yes | No | No | Not to be deter-mined | No | No | No | No | Yes (categories, continuous) | Clearly defined; Valid; Reliable; Implemented consistently | No | Clearly defined; Valid; Reliable; Implemented consistently | No | NA | No |
| Reichborn-Kjennerud et al. (2004) | Yes | Yes | No | Yes | Yes | Yes | No | No | No | Clearly defined; Implemented consistently | No | Implemented consistently | No | NA | Yes |
| Riccobono et al. (2019) | Yes | No | No | Not to be deter-mined | Yes | No | No | No | Yes (categories, continuous) | Clearly defined; Valid; Reliable; Implemented consistently | No | Clearly defined; Valid; Reliable; Implemented consistently | No | NA | No |
| Riccobono et al. (2020) | Yes | No | No | Not to be deter-mined | Yes | No | No | No | Yes (categories, continuous) | Clearly defined; Valid; Reliable; Implemented consistently | No | Clearly defined; Valid; Reliable; Implemented consistently | No | NA | No |
| Richardson et al. (2024) | Yes | No | No | Yes | No | No | Yes | Yes | Yes (categories, continuous) | Clearly defined; Valid; Reliable; Implemented consistently | Yes | Clearly defined; Valid; Reliable; Implemented consistently | No | Yes | Yes |
| Rosenbaum et al. (2023) | Yes | No | No | Not to be deter-mined | Yes | No | No | No | Yes (categories, continuous) | Clearly defined; Implemented consistently | No | Clearly defined; Valid; Reliable; Implemented consistently | No | NA | Yes |
| Sahlan et al. (2023) | Yes | Yes | No | Yes | No | No | No | No | Yes (categories, continuous) | Clearly defined; Valid; Reliable; Implemented consistently | No | Clearly defined; Valid; Reliable; Implemented consistently | No | NA | Yes |
| Schmidt & Randler (2010) | Yes | No | No | Yes | No | No | No | No | Yes (categories, continuous) | Clearly defined; Valid; Reliable; Implemented consistently | No | Clearly defined; Valid; Reliable; Implemented consistently | No | NA | Yes |
| Seigel et al. (2004) | Yes | Yes | Yes | Yes | No | No | No | No | No | Clearly defined; Implemented consistently | No | Not to be determined | No | NA | No |
| Soares et al. (2011) | Yes | No | No | Not to be deter-mined | No | No | No | No | Yes (categories, continuous) | Clearly defined; Valid; Reliable; Implemented consistently | No | Clearly defined; Implemented consistently | No | NA | Yes |
| Suna & Ayaz (2022) | Yes | Yes | No | Yes | Yes | No | No | No | No | Clearly defined; Valid; Reliable; Implemented consistently | No | Clearly defined; Valid; Reliable; Implemented consistently | No | NA | Yes |
| Tăut et al. (2018) | Yes | No | No | Yes | Yes | No | No | No | No | Clearly defined; Implemented consistently | No | Clearly defined; Implemented consistently | No | NA | No |
| Tholin et al. (2009) | Yes | Yes | Yes | Yes | Yes | No | No | No | No | Clearly defined; Implemented consistently | No | Clearly defined; Implemented consistently | No | NA | Yes |
| Trace et al. (2012) | Yes | Yes | Yes | Yes | Yes | No | No | No | Yes (categories, continuous) | Clearly defined; Implemented consistently | No | Clearly defined; Implemented consistently | No | NA | Yes |
| Uyar et al. (2023) | Yes | No | No | Yes | Yes | No | No | No | Yes (categories, continuous) | Clearly defined; Valid; Reliable; Implemented consistently | No | Clearly defined; Valid; Reliable; Implemented consistently | No | NA | Not to be deter-mined |
| Vrabec et al. (2022) | Yes | Yes | No | Not to be deter-mined | Yes | No | No | No | Yes (categories, continuous) | Clearly defined; Valid; Reliable; Implemented consistently | No | Clearly defined; Valid; Reliable; Implemented consistently | No | NA | Yes |
| Walker et al. (2018) | Yes | No | No | Not to be deter-mined | No | No | No | No | Not to be determined | Clearly defined; Implemented consistently | No | Clearly defined; Implemented consistently | No | NA | Yes |
| White et al. (2024) | Yes | Yes | Not to be deter-mined | No | Yes | Yes | No | No | Yes (categories, continuous) | Clearly defined; Valid; Reliable; Implemented consistently | No | Clearly defined; Valid; Reliable; Implemented consistently | No | NA | No |
| Wroblevski et al. (2022) | Yes | No | Yes | Not reported | Not reported | No | No | No | No | Clearly defined; Implemented consistently | No | Clearly defined; Implemented consistently | No | NA | No |
| Wu et al. (2021) | Yes | Yes | Not to be deter-mined | Yes | Yes | No | No | No | Yes (categories, continuous) | Clearly defined; Valid; Reliable; Implemented consistently | No | Clearly defined; Valid; Reliable; Implemented consistently | No | NA | Yes |
| Yeh & Brown (2014) | Yes | No | No | No | Yes | No | No | No | Yes (categories, continuous) | Clearly defined; Valid; Reliable; Implemented consistently | No | Clearly defined; Valid; Reliable; Implemented consistently | No | NA | Yes |
| Yilmaz Yavuz & Altinsoy (2022) | Yes | Yes | Not to be deter-mined | Not to be deter-mined | Yes | Yes | No | No | Yes (categories, continuous) | Clearly defined; Valid; Reliable; Implemented consistently | No | Clearly defined; Valid; Reliable; Implemented consistently | No | NA | No |
