# Supplementary material for: The Association Between Disordered Eating and Sleep in Non‐Clinical Populations—A Systematic Review and Meta‐Analysis
Source: J Sleep Res. 2025 Jun 30;35(2):e70117. doi: 10.1111/jsr.70117 (PMC13003291; doi:10.1111/jsr.70117)
Supplement: Supplementary file 3 — TABLE S3: Narrative overview of mediators and moderators identified for the association of sleep and disordered eating. [file JSR-35-e70117-s003.docx]

Supplementary Table 3. Narrative Overview of Mediators and Moderators Identified for the Association of Sleep and Disordered Eating

| **Author(s)** | **Identified Mediators and Moderators** | **Moderation/Mediation Outcomes** |
| --- | --- | --- |
| Akram et al. (2021) | Sleep-associated monitoring and body image-related distortions and coping processes as mediators for the association between body image disturbance and insomnia symptoms | Sleep-related monitoring on awakening, cognitive distortion of body image, and negative coping strategies related to body image (i.e., appearance fixing, avoidance, rationale acceptance) mediated the relationship between body image disturbance and insomnia symptoms |
| al Balushi & Carciofo (2023) | Skipping breakfast as a potential mediator in the relationship between chronotype and binge eating | Breakfast skipping significantly mediated the association between morningness-eveningness and binge-eating. |
| Ceylan et al. (2024) | Psychological pain and social jetlag (SJL) as mediators for the relationship between chronotype and food addiction | When the mediating role of psychological pain and SJL in this relationship was evaluated, these two variables explained 20.6% of the relationship between chronotype and food addiction, and a significant part of the relationship was due to the direct effect. In short, the relationship between chronotype and food addiction is pretty strong without any mediating variables. The role of SJL in this picture remains unclear. |
| De Young et al. (2022) | Timing of light exposure as a mediator for the relationship between sleep/wake time preferences and morning anorexia/evening hyperphagia; sleep efficiency as a moderator for the relationship between chronotype and morning anorexia/evening hyperphagia | The timing of light exposure mediated the link between sleep/waketime preferences and evening hyperphagia, but not morning anorexia, such that a later peak in light exposure mitigated some of the risk for evening hyperphagia that was associated with later sleep/waketime preferences. Earlier sleep/waketime preference was associated with less morning anorexia, and the strength of this relationship increased with better sleep efficiency. Thus, both a tendency to rise and feel more energetic earlier in the day, coupled with spending a larger portion of time in bed asleep, appears to be especially protective from a delayed pattern of food intake characterized by a lack of morning appetite. However, with poorer sleep efficiency, there appears to be greater risk for morning anorexia; conversely there is greater risk of morning anorexia among individuals with later sleep/waketime preferences regardless of their sleep efficiency. For evening hyperphagia, there was no evidence that the negative relation with sleep/waketime preference depended upon sleep efficiency. |
| Gallant et al. (2013) | Weight status (underweight/normal weight vs overweight/obese) moderating the relationship between repeated weight loss behaviours and short sleep duration | Current weight status moderated the relationship between repeated weight loss behaviours and short sleep duration. The adjusted odds of reporting restrictive eating tendencies, perceived stress and short sleep were significantly elevated only among under-weight/normal-weight individuals who reported repeated weight loss behaviours. |
| Gundogdu & Yildirim (2023) | Anxiety, depression and stress as mediators for the relationship between insomnia/daytime sleepiness and night eating | Higher insomnia scores were a statistically significant risk factor for elevated NES scores. When mediator influences were examined, the indirect influence of the SCOPA-insomnia on the NES scores via DASS-21-depression, and DASS-21-stress were significant, but not via DASS anxiety. SCOPA-daytime sleepiness scores had a direct influence on the NES scores. and higher daytime sleepiness scores were a statistically significant risk factor for elevated NEW scores. When mediator influences were examines, the indirect influence of the SCOPA-daytime sleepiness on the NES scores via DASS-21-depression, and DASS-21-stress were significant, but again not via DASS anxiety. |
| Hafstad et al. (2013) | Gender moderating the relationship between sleeping patterns and eating problems (n.s.) | No moderation effect was identified, suggesting that factors associated with disordered eating among boys are similar to those found with girls. |
| Kandeger et al. (2019) | Impulsivity and insomnia as mediators for the relationship between chronotype and food addiction | Being classified as evening-type increased the likelihood of food addiction significantly by increasing the chances of insomnia and impulsivity scores |
| Lee & Suh (2018) | Depression and anxiety as mediators for the association between nightmares and night eating | Anxiety, and not depression, was a significant mediator in the relationship between nightmare and night eating in female undergraduate students. |
| Lombardo et al. (2014) | Depression as a mediator for the relationship between insomnia severity and disordered eating | The mediation analysis evidenced that both the direct path linking insomnia symptoms and eating disorder symptoms are significant as well as the indirect paths related to the mediation of depression |
| Richardson et al. (2024) | Pre-sleep repetitive negative thinking (RNT)/general repetitive negatives thinking mediates the relationship between worse sleep (latent factor consisting of chronotype, sleep duration, and sleepiness) and eating disorder symptoms | Across all waves, worse adolescent sleep predicted more presleep RNT, which, in turn, predicted increases in eating-disorder symptoms. The indirect path from adolescent sleep to eating disorder symptoms was partially mediated by presleep RNT from Wave 1 to Wave 3 (β = −0.04, SE = 0.013, p = .001), Wave 2 to Wave 4 (β =−0.04, SE = 0.013, p = .001), Wave 3 to Wave 5 (β = −0.04, SE = 0.013, p = .001), and Wave 4 to Wave 6 (β = −0.04, SE = 0.013, p = .001). Across all waves, worse adolescent sleep predicted more general RNT, which, in turn, predicted increases in eating-disorder symptoms. Worse adolescent sleep was also directly related to worsening eating disorder symptoms. In contrast, eating disorder symptoms did not predict changes in adolescent sleep or general RNT over time, and general RNT did not predict changes in sleep. The indirect path from adolescent sleep to eating-disorder symptoms was partially mediated by general RNT from Wave 1 to Wave 3 (β = −0.03, SE = 0.009, p = .009), Wave 2 to Wave 4 (β = −0.03, SE = 0.009, p = .009), Wave 3 to Wave 5 (β = −0.03, SE = 0.009, p= .009), and Wave 4 to Wave 6 (β = −0.03, SE = 0.009, p = .009). |
| Rosenbaum et al. (2023) | Anxiety, stress, depression as mediators for the association between sleep duration and body appreciation/appearance evaluation | For the relationship between sleep duration and body appreciation, there were significant indirect effects through both depression and stress, but not through anxiety. For the relationship between sleep duration and body appreciation, there was a significant indirect effect through depression, but not through stress or anxiety. |
| Wu et al. (2021) | Problematic smartphone use, depression and anxiety as mediators for the relationship between sleep quality and disordered eating | Results of serial multiple mediation analyses indicated that problematic smartphone use and psychological distress (i.e., anxiety and depression) fully mediated the relationship between sleep quality and disordered eating behaviours |
